# Design, synthesis and *in vitro* biological studies of novel triazoles with potent and broad-spectrum antifungal activity

**DOI:** 10.1080/14756366.2023.2244696

**Published:** 2023-08-08

**Authors:** Junhe Bao, Yumeng Hao, Tingjunhong Ni, Ruina Wang, Jiacun Liu, Xiaochen Chi, Ting Wang, Shichong Yu, Yongsheng Jin, Lan Yan, Xiaomei Li, Dazhi Zhang, Fei Xie

**Affiliations:** a Department of Organic Chemistry, School of Pharmacy, Naval Medical University, Shanghai, China; b Department of Pharmacy, Shanghai Tenth People’s Hospital, School of Medicine, Tongji University, Shanghai, China; c Center of New Drug Research, School of Pharmacy, Naval Medical University, Shanghai, China; d School of Traditional Chinese Materia Medica, Shenyang Pharmaceutical University, Shenyang, China; e Department of Stomatology, Changhai Hospital, Naval Medical University, Shanghai, China

**Keywords:** Triazole derivatives, antifungal activity, structure-activity relationships, Cyp51

## Abstract

A series of novel triazole derivatives containing aryl-propanamide side chains was designed and synthesised. *In vitro* antifungal activity studies demonstrated that most of the compounds inhibited the growth of six human pathogenic fungi. In particular, parts of phenyl-propionamide-containing compounds had excellent, broad-spectrum antifungal activity against *Candida albicans* SC5314, *Cryptococcus neoformans* 22-21, *Candida glabrata* 537 and *Candida parapsilosis* 22-20 with MIC values in the range of ≤0.125 µg/mL–4.0 µg/mL. In addition, compounds **A1**, **A2**, **A6**, **A12** and **A15** showed inhibitory activities against fluconazole-resistant *Candida albicans* and *Candida auris*. Preliminary structure-activity relationships (SARs) are also summarised. Moreover, GC-MS analysis demonstrated that **A1**, **A3**, and **A9** interfered with the *C. albicans* ergosterol biosynthesis pathway by inhibiting Cyp51. Molecular docking studies elucidated the binding modes of **A3** and **A9** with Cyp51. These compounds with low haemolytic activity and favourable ADME/T properties are promising for the development of novel antifungal agents.

## Introduction

**Figure 1.**
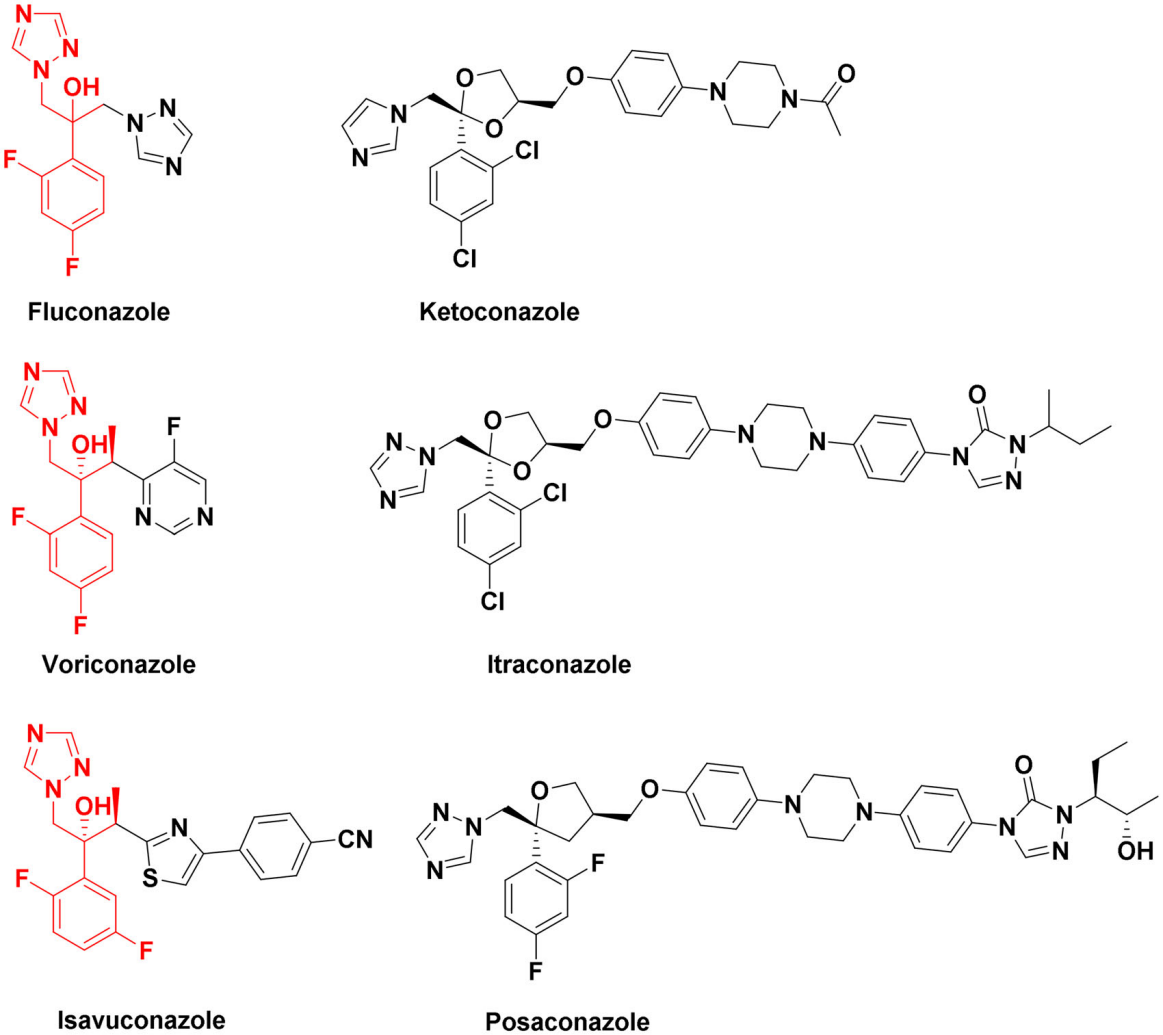
Structures of the triazole antifungal agents and novel triazole compounds.

In recent years, invasive fungal infections (IFIs), such as invasive candidiasis and aspergillosis, pose an increasingly serious threat to human health^1^^,^^2^. A retrospective study based on single-centre autopsy data from China (3,447 cases) showed that the prevalence of IFIs has been steadily increasing for decades^3^. The mortality rates of IFIs in China are also on the rise with estimates ranging from 36.6% to 60%^4^. On one hand, long-term use of fluconazole has caused the widespread emergence of fluconazole resistance in *Candida albicans* which usually results in clinical treatment failure in settings where alternative treatment options are not available. On the other hand, COVID-19-associated fungal infections continue to increase, especially in critically ill, hospitalised, or immunocompromised patients^5^. Azoles, such as fluconazole, ketoconazole, voriconazole and itraconazole, etc. (Figure 1), are frequently used antifungal agents in clinical practice. Azoles primarily target fungal Cyp51 (lanosterol 14α-demethylase). The pharmacophore of antifungal triazoles contains a triazole ring, a hydroxyl group and a dihalophenyl linked to a two-carbon chain as shown in Figure 1. The major difference between the structures of novel triazole analogues is on the side chains attached to the pharmacophore^6–8^.

**Figure 2.**
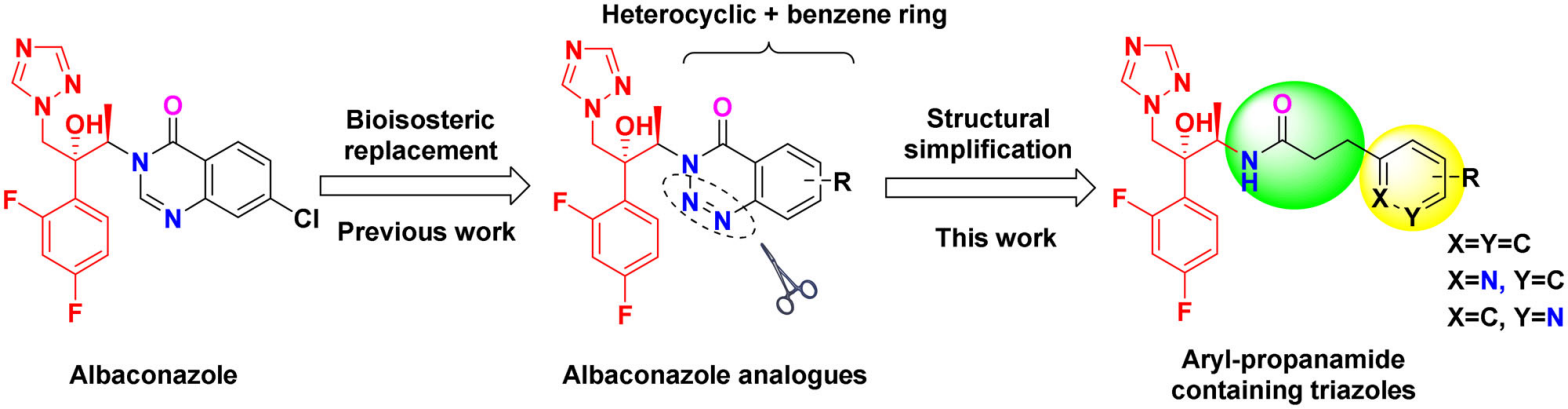
Design strategy of target compounds.

In our previous work, we designed and synthesised a series of albaconazole analogues by replacing the quinazolinone unit with its bioisostere 1,2,3-benzotriazin-4-one (Figure 2). Most analogues exhibited improved *in vitro* antifungal activities compared to fluconazole. Inspired by the amide moiety that existed in the quinazolinone and 1,2,3-benzotriazin-4-one, we conceived that if we further simplified the *N*-heterocycle at the same time as keeping the amide linker with various aromatic rings, it would give novel, potent triazoles. Hence, in this study, a series of aryl-propanamide side chains containing triazole derivatives were rationally designed, synthesised and their *in vitro* antifungal activities were further evaluated.

## Results and discussion

### Chemistry

**Scheme 1.**
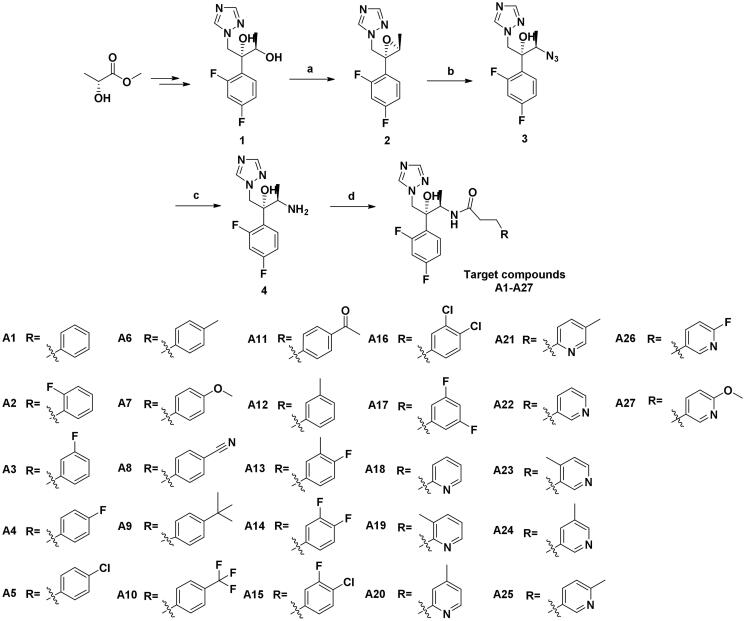
Synthesis of the target compounds. (a) (i) Et_3_N, MsCl, DCM, 0 °C, 1 h; (ii) NaOH, H_2_O, 0 °C, 4 h; (b) NH_4_Cl, NaN_3_, DMF, 80 °C, 10 h; (c) Pd/C, H_2_, MeOH, r.t., 8 h; (d) Substituted propionic acid, PyBOP, DIEA, DMF, r.t., 5 h.

**Figure 3.**
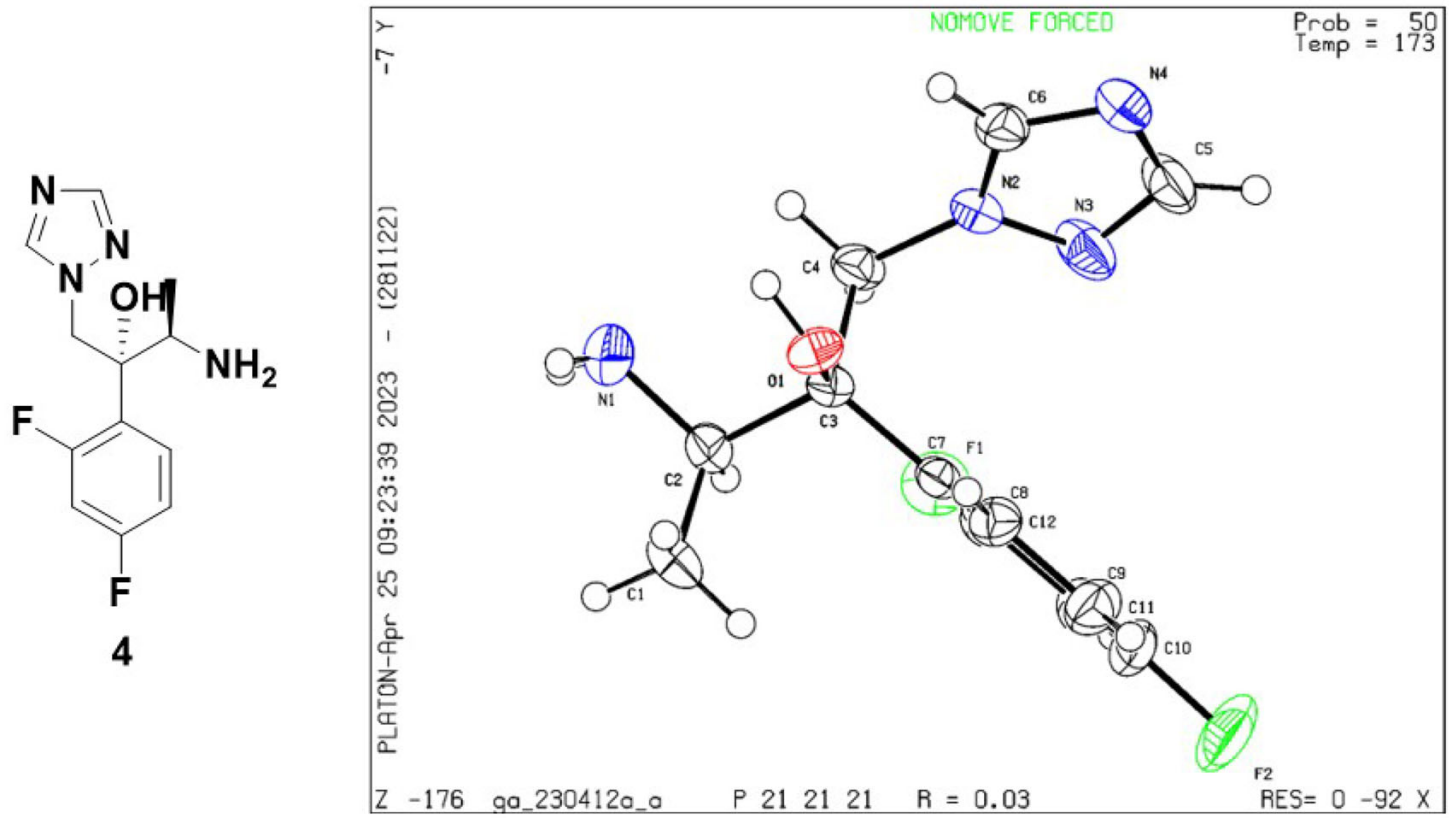
The single-crystal structure of amine **4**.

According to Scheme 1, twenty-seven novel triazole derivatives were synthesised. The key intermediate (2 *R*,3*R*)-2-(2,4-difluorophenyl)-1-(1*H*-1,2,4-triazol-1-yl)butane-2,3-diol (**1**) was prepared as in our previous work^9^. In the next step, triazolyl-diol **1** was converted into methane sulphonate and then eliminated to give epoxide **2**. Epoxide **2** was reacted with NaN_3_ to form ring-opening azide compound **3**. Next, **3** was subjected to catalytic hydrogenation to afford amine compound **4**, which was followed by a condensation reaction with various commercially available substituted propionic acids to obtain target compounds **A1-A27**. The chiral structure of key intermediates amine **4** was clearly confirmed by X-ray crystallographic analysis. The atom labelling and thermal ellipsoids of amine **4** are shown in Figure 3. It crystallised in the orthorhombic crystal system and P2**_1_**2**_1_**2**_1_** space group with *a* = 8.9511(3) Å, *b* = 10.4014(4) Å, *c* = 13.8084(4) Å, *α* = 90°, *β* = 90° and *δ* = 90° (Table S1 in the supplementary material). Based on the results, the absolute configuration at C-2 and C-3 in amine **4** was determined to be *R*. All the newly synthesised compounds were characterised and confirmed by ^1^H NMR, ^13^C NMR, and LC-MS as described in Supporting Information.

### In vitro antifungal activity and structure-activity relationships

According to the Clinical Laboratory Standards Institute (CLSI, M27-A3 and M60) protocols^10^^,^^11^, the target compounds were evaluated for their *in vitro* antifungal activity by the broth microdilution method. The fungi strains used in this study were obtained from the American Type Culture Collection (ATCC) and Changhai Hospital (Shanghai, China). The MIC was defined as the minimum concentration of drugs to inhibit ≥80% growth of fungal cells compared with drug-free control. Fluconazole (FCZ) and ketoconazole (KCZ) were used as positive controls.

As shown in Table 1, the majority of the 17 phenyl-propionamide-containing compounds (**A1-A17**), except for **A8** and **A11**, showed strong antifungal activity against *Candida albicans* SC5314 and *Candida glabrata* 537 with MIC values ranging from 0.125 µg/mL to 0.5 µg/mL, which were comparable to or greater than FCZ and KCZ. Good inhibitory activity was exhibited against *Candida parapsilosis* 22–20 and *Cryptococcus neoformans* 22–21 with MIC ranging from 0.5 µg/mL to 4.0 µg/mL and ≤0.125 µg/mL to 1.0 µg/mL, respectively. FCZ had no inhibitory effect (MIC > 64.0 µg/mL) on either *Candida tropicalis* 8915 or *Aspergillus fumigatus* 7544. Nine compounds (**A2**, **A3**, **A10**, **A12–A17)** were at least 2-fold more effective than FCZ with MIC 8.0 µg/mL–64.0 µg/mL. Of note, compounds **A2**, **A3**, **A10**, **A12–A17** exhibited broad-spectrum antifungal against six fungal strains. However, compounds containing pyridinyl-propionamide (**A18–A27**) showed weaker activity *in vitro* and had a narrow spectrum compared with phenyl-propionamide compounds. For example, all of the pyridinyl-propionamide derivatives had no activity against either *Candida tropicalis* 8915 and *Aspergillus fumigatus* 7544. Similar to the above results, the introduction of a pyridine ring also resulted in the loss of antifungal activity against *C. albicans*, *C. glabrata*, *C. parapsilosis* and *C. neoformans*.

**Table 1.** *In vitro* antifungal activity of the target compounds against tested fungi (MIC, μg/mL).

Compd.	*C. alb* SC5314	*C. gla* 537	*C. par* 22-20	*C. neo* 22-21	*C. tro* 8915	*A. fum* 7544
**A1**	≤0.125	≤0.125	0.5	0.25	>64.0	64.0
**A2**	≤0.125	≤0.125	0.5	0.25	16.0	32.0
**A3**	≤0.125	≤0.125	0.25	0.25	8.0	16.0
**A4**	0.25	≤0.125	0.5	0.25	>64.0	64.0
**A5**	≤0.125	≤0.125	0.5	≤0.125	>64.0	32.0
**A6**	≤0.125	≤0.125	1.0	0.5	>64.0	64.0
**A7**	0.5	0.5	1.0	0.25	>64.0	64.0
**A8**	1.0	1.0	4.0	1.0	>64.0	>64.0
**A9**	≤0.125	≤0.125	0.25	≤0.125	>64.0	32.0
**A10**	1.0	≤0.125	2.0	0.25	16.0	64.0
**A11**	2.0	1.0	4.0	1.0	>64.0	>64.0
**A12**	≤0.125	≤0.125	0.5	0.25	32.0	32.0
**A13**	≤0.125	≤0.125	0.5	0.25	32.0	64.0
**A14**	≤0.125	≤0.125	1.0	1.0	64.0	64.0
**A15**	≤0.125	≤0.125	0.5	0.5	8.0	32.0
**A16**	≤0.125	≤0.125	0.5	≤0.125	8.0	64.0
**A17**	≤0.125	≤0.125	0.5	0.5	16.0	32.0
**A18**	2.0	2.0	8.0	16.0	>64.0	>64.0
**A19**	1.0	1.0	2.0	2.0	>64.0	>64.0
**A20**	1.0	2.0	4.0	4.0	>64.0	>64.0
**A21**	2.0	2.0	4.0	4.0	>64.0	>64.0
**A22**	16.0	8.0	64.0	16.0	>64.0	>64.0
**A23**	64.0	1.0	16.0	8.0	>64.0	>64.0
**A24**	16.0	4.0	32.0	32.0	>64.0	>64.0
**A25**	16.0	2.0	64.0	16.0	>64.0	>64.0
**A26**	2.0	1.0	8.0	8.0	>64.0	>64.0
**A27**	4.0	2.0	8.0	8.0	>64.0	>64.0
**KCZ**	4.0	1.0	0.5	0.125	16.0	4.0
**FCZ**	0.5	1.0	4.0	1.0	>64.0	>64.0

C. alb: Candida albicans; C. par: Candida parapsilosis; C. gla: Candida glabrata; C. tro: Candida tropicalis; C. neo: Cryptococcus neoformans; A. fum: Aspergillus fumigatus; KCZ: ketoconazole; FCZ: fluconazole.

Preliminary structure-activity relationships (SARs) show a correlation between *in vitro* antifungal activities and chemical structures. Overall, the phenyl-propanamide containing triazoles inhibited fungi better than the corresponding pyridinyl analogues. Moreover, the antifungal effect of *meta*-F-substituted compound (**A3**) on the terminal phenyl is better than that of the *ortho* and *para*-F-substituted (**A2** and **A4**) against *C. tropicalis* and *A. fumigatus*. Interestingly, among the *para*-substituted compounds (**A4–A11**), almost all compounds had no activity against *C. tropicalis*, except for **A10**. Furthermore, compounds with electron-withdrawing groups in *para*- position of cyan (**A8**), trifluoromethyl (**A10**), and acetyl group (**A11**) resulted decreased activity against *C. albicans* and *C. parapsilosis*. Replacement of *meta*-F with CH_3_ yielded **A12** which had a similar potency to **A3** against tested fungi. The introduction of another F or Cl atom to the terminal phenyl, whilst keeping the *meta*-substituted position, yielded the di-substituted compounds **A13–A17**. These displayed excellent and broader-spectrum *in vitro* activity against all of the tested strains. For example, **A13–A17** had the best MIC values (≤ 0.125 µg/mL) against *C. albicans* and *C. glabrata*. When compared with 2-pyridinyl containing series **A18–A21** (H or different CH_3_ substituted position) and related 3-pyridinyl analogues **A22**-**A25**, **A18–A21** had relatively greater activity against *C. glabrata*, *C. parapsilosis* and *C. neoformans*, and was even more efficacious against *C. albicans*.

### Evaluation of MIC against FCZ-resistant strains

The alarming impact of drug resistance amongst *Candida* spp. is an emerging problem globally^12^. Rising rates of azole-resistant *Candida* spp. are associated with an increase in treatment failures. *Candida auris* is characterised by a high level of multi-drug resistance. It has been reported in more than 45 countries and has caused serious hospital outbreaks with crude mortality rates as high as 72%^13^^,^^14^. Therefore, we further evaluated the *in vitro* antifungal activity of our compounds against fluconazole-resistant strains by the microdilution method assay.

As shown in Table 2, the MIC values of FCZ against *C. albicans* 901 and *C. auris* 918 and 919 were greater than 256.0 µg/mL, indicating that these isolates were highly FCZ-resistant. These triazole derivatives exhibited moderate inhibitory effects against *C. albicans* 901. **A1** and **A5** showed the best activity with MIC of 1.0 µg/mL against *C. alb 901*. **A1**, **A2**, **A6**, **A12** and **A15** demonstrated inhibitory effects against the two *C. auris* strains with MIC of 32.0 µg/mL or 64.0 µg/mL. The above results demonstrated that with appropriate structural modifications on the triazole compound side chains, the problem of drug resistance could be overcome.

**Table 2.** *In vitro* antifungal activity of the potent compounds against FCZ-resistant *C. albicans* and *C. auris* isolates measured by MIC (μg/mL).

Compd.	*C.alb* 901	*C.auris* 918	*C.auris* 919
**A1**	1.0	64.0	32.0
**A2**	16.0	32.0	32.0
**A3**	16.0	>64.0	64.0
**A4**	16.0	>64.0	>64.0
**A5**	1.0	>64.0	>64.0
**A6**	64.0	64.0	32.0
**A9**	32.0	>64.0	32.0
**A10**	16.0	>64.0	>64.0
**A12**	8.0	64.0	64.0
**A13**	2.0	>64.0	>64.0
**A14**	4.0	>64.0	>64.0
**A15**	8.0	64.0	64.0
**A16**	2.0	>64.0	>64.0
**A17**	4.0	>64.0	>64.0
**FCZ**	>256.0	>256.0	>256.0

*C. alb*: *Candida albicans*; *C. auris*: *Candida auris*; FCZ: fluconazole.

### GC-MS analysis of sterol composition in C. albicans

**Figure 4.**
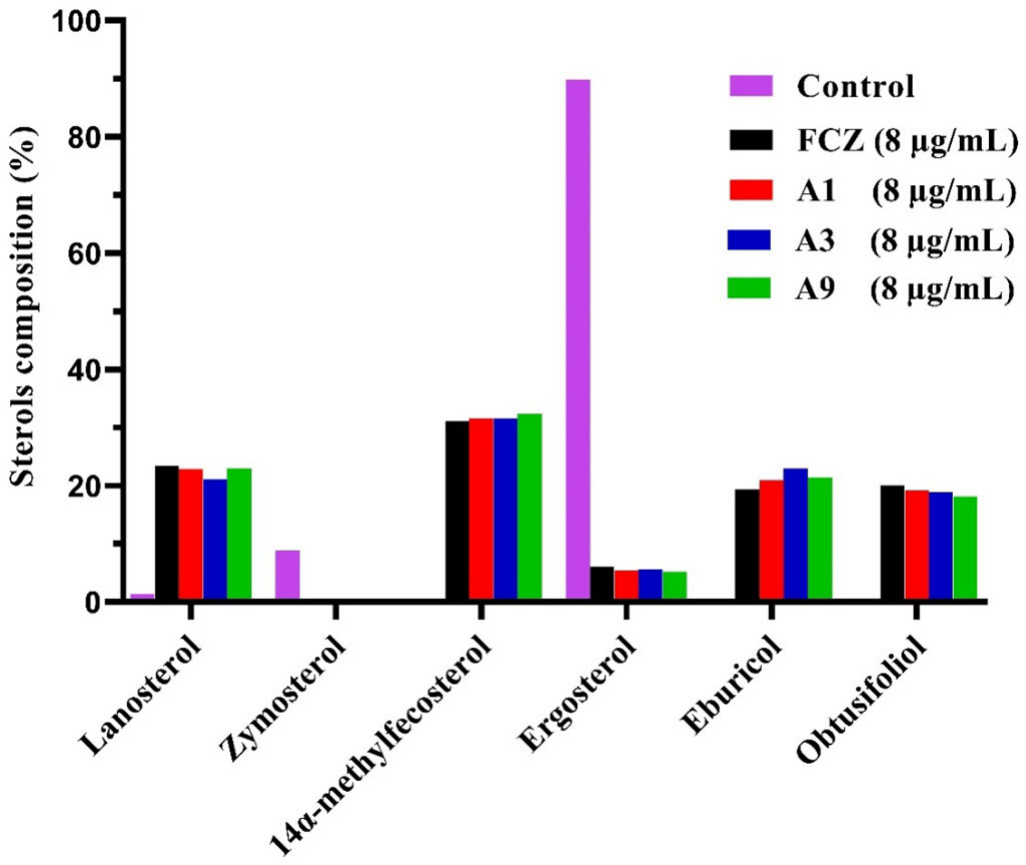
GC-MS analysis of sterols in *C. albicans* cells. The fungal strain was treated with DMSO (Control), FCZ, compounds **A1**, **A3** or **A9** at 8 μg/mL for 8 h.

Gas chromatography-mass spectrometry (GC-MS) was used to examine the composition of sterols in fungal cells as a preliminary method to verify whether our target compounds inhibit Cyp51^15^. Therefore, compounds **A1**, **A3**, **A9** and FCZ at 8 µg/mL were used to treat *C. albicans* SC5314, and their sterol profiles were analysed as shown in Figure 4. As the major fungal sterol, ergosterol accounted for 89.85% of the total sterols in the control group, zymosterol accounted for 8.80%, in addition to several other sterols. When treated with either FCZ, **A1**, **A3**, or **A9** the ergosterol content of the fungal cells decreased to 6.08%, 5.44%, 5.63% and 5.17%, respectively. In contrast, the content of other sterols in the bypass pathway increased. For instance, treatment with **A1** resulted in the following: the proportion of lanosterol, 14α-methylfecosterol, eburicol and obtusifoliol increased to 23.42%, 31.11%, 19.29% and 20.09%, respectively. Our target compounds had a consistent effect and the same mechanism of action as FCZ by inhibiting the production of ergosterol on Cyp51.

### Evaluation of hemolysis

**Figure 5.**
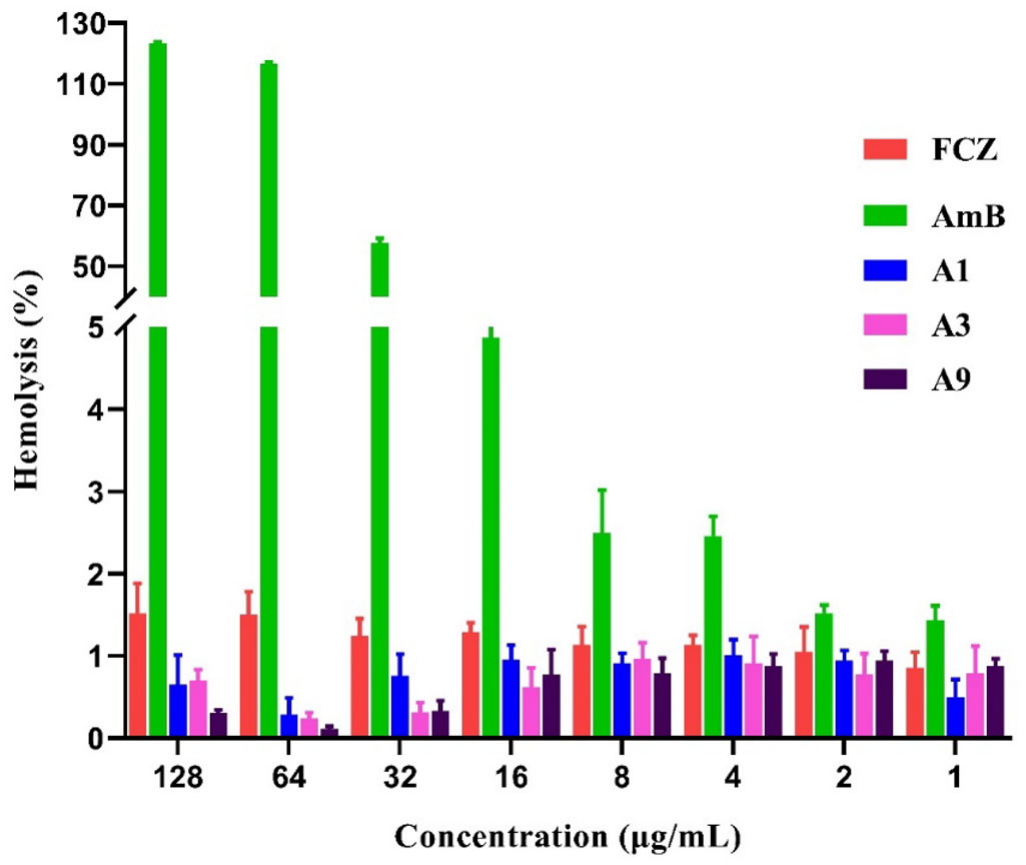
Haemolytic effect of **A1**, **A3** and **A9** against rabbit red blood cells at different indicated concentrations.

Haemolysis rate is an important indicator of drug safety^16^. We performed rabbit haemolysis experiments to evaluate the safety of our synthesised compounds. At a concentration of 16 µg/mL, AmB haemolysis averaged 4.87%, while FCZ, **A1**, **A3** and **A9** haemolysis averaged 1.30%, 0.95%, 0.61%, and 0.78%, respectively, demonstrating minimal effect to the rabbit red cells (Figure 5). When AmB was given at 32 µg/mL, it caused significant haemolysis (57.70%), and at 64 µg/mL, it resulted in 100% haemolysis. In contrast, even at the highest tested concentration (256 µg/mL), FCZ, **A1**, **A3** and **A9** only induced 1.52%, 0.64%, 0.71% and 0.31% haemolysis, respectively, indicating that they did not cause significant haemolysis and that our compounds have a similar safety profile as FCZ in rabbit red blood cells.

### Molecular docking study

**Figure 6.**
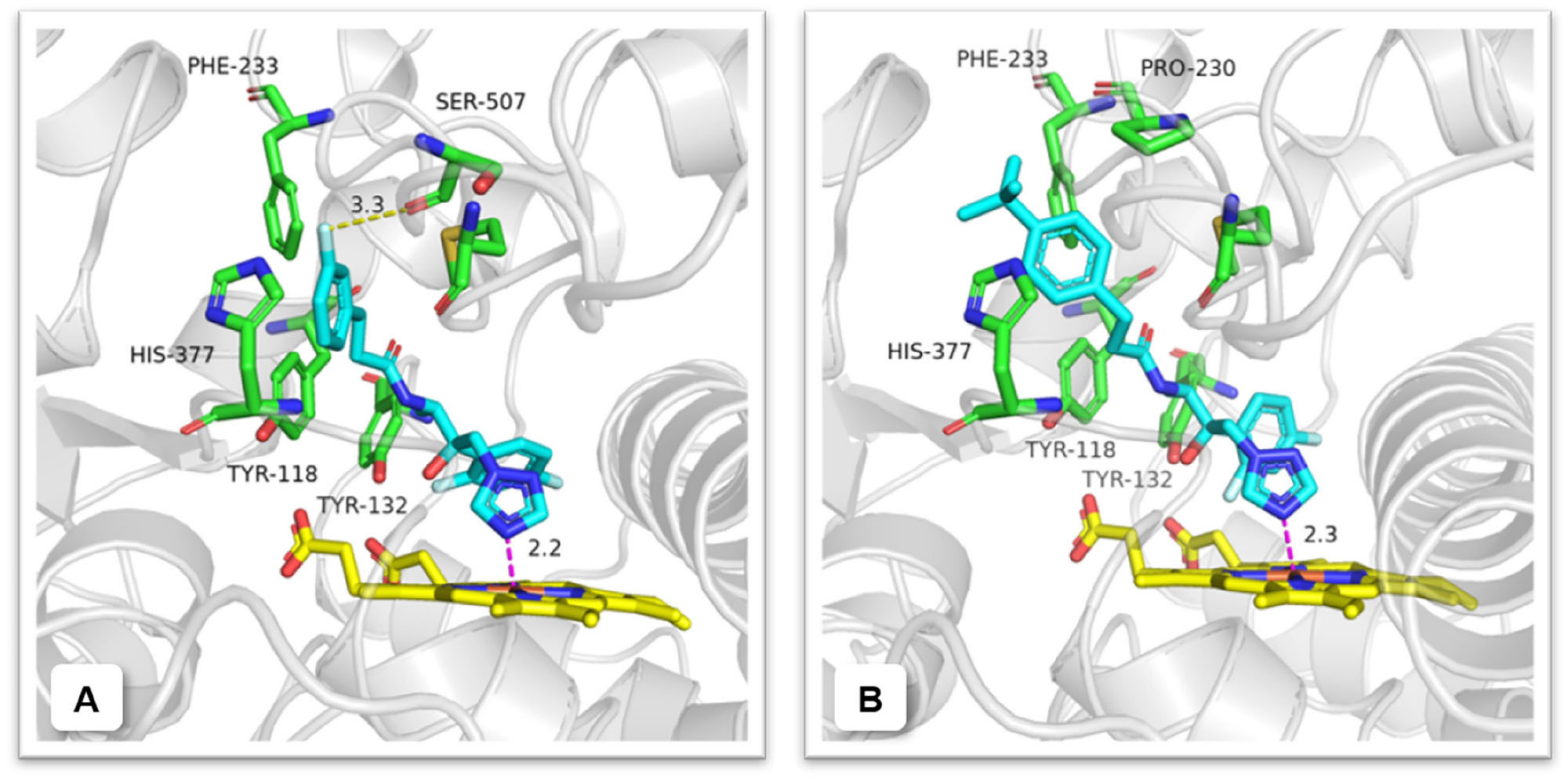
Predicted binding mode of compounds **A3** and **A9** (blue) in the active site of CYP51. Yellow dashed lines represent the halogen bond interactions and magenta dashed lines represent the coordination bond. The image was generated using PyMol.

To investigate the binding mode of target compounds, a molecular docking study was carried out using the Surflex-Dock module in the SYBYL-X 2.0. Representative compounds **A3** and **A9** (MIC ≤0.125 µg/mL against *C. albicans*) were docked into *C. albicans* Cyp51 (PDB ID: 5TZ1) active site. As depicted in Figure 6(A), the N(4) atom of the 1,2,4-triazole formed a coordination bond with the haem Fe-atom, and the hydroxyl group and difluorophenyl group were considered as forming nonbonding interactions like Tyr132. The phenyl-propanamide side chain stretched into the Cyp51 channel to form hydrophobic and van der Waals interactions with surrounding residues such as Tyr118, Phe233 and His377. Notably, the 3-fluorine atom on the terminal phenyl of compound **A3** was found to form an additional halogen bond with Ser507 in the distance of 3.3 Å, while compound **A9** (Figure 6(B)), shared a similar binding mode with **A3**, mainly interacted with surrounding residues by hydrophobic and van der Waals. In addition, the docking of two selected compounds **A3** and **A9** with proper fitting into the binding site exhibited binding free energies (Δ*G*_b_) of −169.50 and −142.78 kcal/mol, respectively, corresponding to the reference drug fluconazole (Δ*G*_b_: −123.85 kcal/mol). This result was correlated to their antifungal potency against *C. albicans*.

### Theoretical evaluation of ADME/T properties

**Figure 7.**
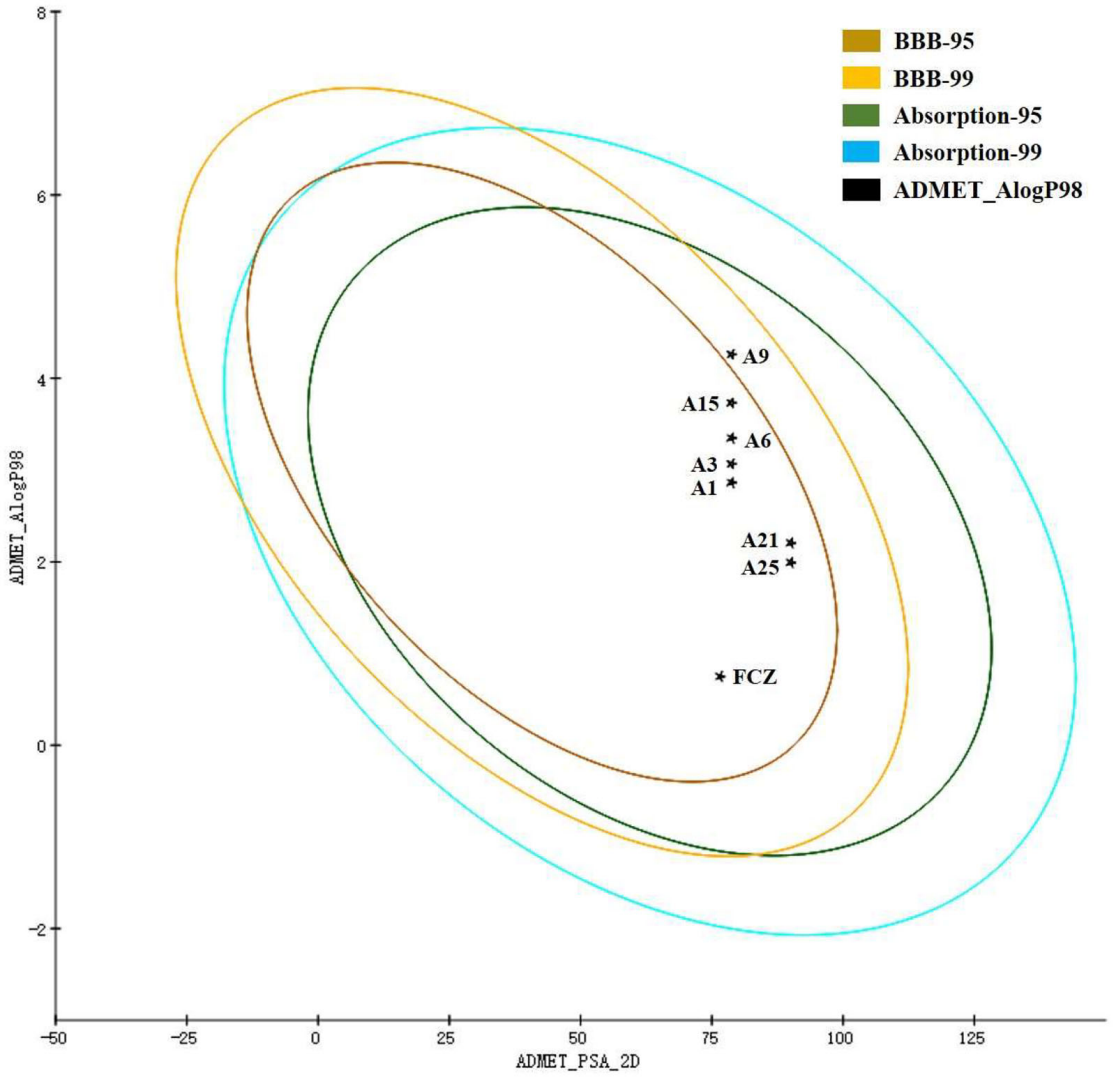
The evaluation egg-chart of PSA and Alog P of tested compounds. The 95% and 99% confidence limit ellipses of blood-brain barrier and intestinal absorption models.

ADMET properties refer to the absorption, distribution, metabolism, excretion and toxicity properties of the molecule in the organism. ADMET properties prove useful in improving the success rate of drug development^17^^,^^18^. Hence, to further investigate our synthesised triazole drugs, compounds **A1**, **A3**, **A6**, **A9**, **A15**, **A21** and **A25** were predicted for *in silico* DS-ADMET and DS-TOPKAT modules. As shown in Figure 7, nearly all compounds are positioned in the 95% and 99% confidence ellipses for the blood-brain barrier (BBB) and human intestinal absorption (HIA), indicating that these compounds have proper aqueous liposolubility and might enter the blood through HIA to exert their antifungal effect *in vivo*, as well as penetrate through BBB to treat fungal infections of the nervous system.

The detailed predicated data of ADME/T were summarised in Table S2. Similar to FCZ, these triazole derivatives had proper absorption or permeation with PSA < 140, −2.0 ≤ A log*P* ≤ 7.0. For toxicity evaluation, these compounds were non-mutagenic, noncarcinogenic, and non-irritating. Although part of the compounds showed a certain degree of skin sensitisation, ocular irritancy or development toxicity potential (DTP) like FCZ, there were still subtle differences between the compounds. For example, **A15** had the characteristics of weak skin sensitisation, **A3** showed mild ocular irritancy, and **A6**, **A21** and **A25** had non-toxic DTP, which reflected the different kinds of substituents and may influence the drug safety profile.

## Conclusion

In summary, based on our previous work, we simplified the quinazoline ring of albaconazole to obtain a series of novel triazoles containing aryl-propanamide side chains. *In vitro,* antifungal activity of the target compounds demonstrated that parts of them exhibited potent and broad-spectrum antifungal activity against tested fungi. SARs study indicated that phenyl-propanamide moiety was good for exerting antifungal activity than pyridinyl. Moreover, compounds **A1**, **A2**, **A6**, **A12** and **A15** not only had excellent antifungal activity against *C. albicans* and *C. glabrata* with MIC ≤ 0.125 µg/mL but also exhibited inhibitory activity against FCZ-resistant strains including *C.auris* isolates. GC-MS mechanism studies showed that selected compounds **A1**, **A3**, and **A9** influenced the ergosterol biosynthesis in a manner similar to that of fluconazole via Cyp51 inhibition. In addition, **A1**, **A3** and **A9** were further evaluated for their ability to induce haemolysis of rabbit red cells. **A1**, **A3** and **A9** induced a similar level of haemolysis to that of FCZ. Molecular docking studies showed that the propanamide moiety side chains extended into the hydrophobic pocket of the CYP51 channel and *m*-F substituted on the terminal phenyl could form halogen interactions with Ser507. Overall, *in silico* evaluation of ADME/T properties of our compounds suggested that these novel triazole analogues are promising drugs. Further structure optimisation and pharmacokinetic evaluation work are currently being undertaken.

## Experiment section

### Synthesis

The solvents and reagents were purchased from Bidepharm (Shanghai, China) or Aladdin (Shanghai, China) without further purification unless otherwise indicated. The column chromatography was performed on silica gel (200–300 mesh). ^1^H and ^13^C NMR spectra were recorded in DMSO-*d_6_* using a Bruker AC-300/400P spectrometer, and TMS as a standard. Chemical shifts (δ values) and coupling constants (*J* values) are measured in ppm and Hz, respectively. ESI mass spectra were performed using an Agilent Technologies 6120 quadrupole LC-MS.

#### General procedure for the synthesis of 1-(((2 R,3S)-2–(2,4-difluorophenyl)-3-methyloxiran-2-yl)methyl)-1H-1,2,4-triazole (2)

Compound **1** (10.0 mmol) was dissolved in DCM (15 ml), and Et_3_N (11.0 mmol) was added at 0 °C, and then methanesulfonyl chloride (MsCl) (11.0 mmol) was slowly added. The mixture was stirred at room temperature for 1 h. After the reaction was complete, 3 M sodium hydroxide solution (approximately 6 ml) was poured into the mixture, then stirred at 0 °C for 4 h, and the reaction was monitored by TLC. After the reaction had finished (monitored by TLC), the organic phase was washed with saturated sodium chloride aqueous solution (3 × 20 ml), dried with anhydrous sodium sulphate and evaporated under vacuum to obtain the crude product of compound **2**, which was recrystallized under the condition of ethyl acetate/petroleum ether as a white solid (yield 60%).

#### General procedure for the synthesis of (2 R,3R)-3-azido-2–(2,4-difluorophenyl)-1-(1H-1,2,4-triazol-1-yl)butan-2-ol (3)

Compound **2** (24.0 mmol) was dissolved in DMF (20 ml) followed by the addition of NaN_3_ (28.8 mmol) and NH_4_Cl (36.0 mmol). The reaction mixture was stirred at 80 °C for 10 h. The reaction was monitored by TLC. After the reaction was completed, the reaction mixture was cooled to room temperature, poured into ice water, and then stirred overnight to produce a large amount of white solid. The crude compound **3** was obtained by filtration and dried, which was directly used in the next step without further purification.

#### General procedure for the synthesis of (2 R,3R)-3-amino-2–(2,4-difluorophenyl)-1-(1H-1,2,4-triazol-1-yl)butan-2-ol (4)

Pd/C was slowly added to a solution of compound **3** in MeOH (20 ml). The mixture was stirred overnight in a hydrogen atmosphere at room temperature. The reaction was monitored by TLC. After the reaction was completed, Pd/C was filtered and the organic solvent was evaporated under reduced pressure to obtain compound **4** as a white solid (yield 68%).

#### General procedure for the synthesis of target compounds (A1–A27)

DIEA (2 mmol) and PyBOP (1.1 mmol) were added to a solution of compound **4** (1.0 mmol) and various substituted aromatic carboxylic acid (1.0 mmol) in DMF (5 ml) at room temperature. The mixture was stirred for 2 h. The reaction was monitored by TLC. After the reaction had finished, the mixture was poured into water, and then the mixture was extracted with ethyl acetate (2 × 10 ml). After washing with brine (2 × 10 ml) and drying over anhydrous Na_2_SO_4_, the organic phase was evaporated in a vacuum. The crude product was purified by silica gel column chromatography using ethyl acetate/petroleum ether (1:1) as the eluent to give target compounds **A1–A27**.

## Biological activity

### Strains and culture conditions

All fungal strains used in this study were obtained from the American Type Culture Collection (ATCC) and Changhai Hospital (Shanghai, China). All strains were cultivated by shaking (200 rpm) at 30 °C in YEPD medium (1% yeast extract, 2% peptone, and 2% dextrose) to an exponential growth stage. Ketoconazole (KCZ), fluconazole (FCZ) and amphotericin B (AmB) were purchased from Sigma and dissolved in dimethyl sulfoxide (DMSO) at a concentration of 6.4 mg/mL as stock solutions.

### Broth microdilution assay

Fungal cells at the exponential growth stage were collected and resuspended in RPMI 1640 medium, then diluted to 1 × 10^3^ cells/mL. The suspension of fungal cells was inoculated into 96-well plates. Antifungal compounds were serially added and diluted in a 2-fold series to a final concentration range of 64.0–0.125 µg/mL. RPMI 1640 medium with no fungal cells served as a blank control. The fungal suspensions containing DMSO and FCZ served as the negative control and the positive control, respectively. *C. albicans*, *C. glabrata*, *C. parapsilosis*, *C. tropicalis*, and *C. auris* cells were incubated at 30 °C for 24 h, *C. neoformans* and *A. fumigatus* cells were incubated at 30 °C for 48 h. The optical density (OD_630_) at 630 nm was measured by a spectrophotometer, which was used to calculate the MIC_80_ values of each compound.

### GC-MS analysis of sterol composition

Overnight cultured *C. albicans* SC5314 fungal cells were collected and diluted to 5 × 10^6^ cells/mL in YEPD medium. The fungal suspension was transferred to 50 ml conical flasks, then 8.0 µg/mL of compounds or fluconazole were added. The fungal cells were cultured by shaking (200 rpm) at 30 °C. After incubation for 8 h, the fungal suspension was centrifuged (3000 rpm, 1 min), harvested and washed twice with distilled water carefully. The saponifying agent (6 ml of 15% NaOH resolved in 90% ethanol) was added to the precipitate. After 1 h of saponification at 80 °C, sterols were extracted with hexane (3 × 5 ml). The combined extracts were evaporated in a water bath and washed once with water. The residue was dried in a 60 °C water bath to obtain the total sterols. Finally, the total sterol was analysed by GC-MS and the structures of each sterol content were identified and compared with the National Institute of Standards and Technology (NIST) database.

### Haemolytic assays

Briefly, 100 µL of fresh rabbit red cell (RBCs) suspension in 96-well plates was mixed with 100 µL of a solution of compounds, FCZ or AmB to the final concentrations of 128–1 µg/mL. RBCs incubated with PBS (containing an equal volume of DMSO) were used as the negative control, and those incubated with distilled water were used as the positive control (100% haemolysis). After incubation for 1 h at 37 °C, the supernatants were centrifuged, collected, and the absorbance (Abs) was measured by using a spectrophotometer at 405 nm. The percentage of haemolysis was calculated using the following equation: haemolysis % = (Abs_S_ − Abs_0_)/(Abs_100_ − Abs_0_) × 100%, where Abs_S_ represented the average absorbance of the sample, Abs_0_ was the average absorbance of the negative control, and Abs_100_ mean the average absorbance of the positive control.

### Computational methodology

The crystal structures of *C. albicans* CYP51 (PDB ID: 5TZ1) were downloaded from the protein database bank. The docking studies were carried out with SYBYL-X 2.0 software. Protein preparation using the standard Tripos molecular mechanics force field with a Gasteiger–Hückel charge, and other staged minimisation was set as default. Then, the ligands and water were removed, and hydrogens were added to the amino acid residues. The binding site was defined as whole residues within a 10 Å radius around the original ligand. Next, the active pocket was generated, and the compound was docked through the Surface Dock module. Finally, the best conformation was chosen to analyse the ligand-protein interaction. The image was further plotted by Pymol.

### ADME/T prediction

Tested compounds were predicted using DS-ADMET and DS-TOPKAT modules^19^^,^^20^. The operation process was performed as follows: the structures of tested compounds were prepared by Chemdraw, and then imported into the program. Two modules ADMET descriptors and Toxicity Prediction were selected. The prediction items, including aqueous solubility, plasma protein binding, intestinal absorption, blood-brain barrier penetration, Ames mutagenicity, FDA rodent carcinogenicity, Skin_Irritancy, Skin_sensitisation, Ocular_Irritancy and DTP (development toxicity potential) were set as research objects. Subsequently, running the program obtains the corresponding prediction results.

### X-ray crystallography

Single crystals of amine **4** were obtained by slow evaporation of dichloromethane solution amine **4** at room temperature. The X-ray single crystal diffraction data for amine **4** were collected on Bruker APEX DUO diffractometers with Mο Kα radiation (*λ* = 1.34139 Å) at 173 ± 2 K in the *ω-2θ* scanning mode. The structures were solved by direct methods using the SHELXS-97A program and refined by full-matrix least-squares techniques (SHELXL-97) on *F*^2^. Anisotropic thermal parameters were assigned to all non-hydrogen atoms. The organic hydrogen atoms were generated geometrically CCDC-2259250 (amine **4**) contains the supplementary crystallographic data for this paper. These data can be obtained free of charge from The Cambridge Crystallographic Data Centre via www.ccdc.cam.ac.uk/data_request/cif.

## Supplementary Material

Supplemental Material
