# Supplementary material for: Design, synthesis and in vitro biological studies of novel triazoles with potent and broad-spectrum antifungal activity
Source: J Enzyme Inhib Med Chem. 2023 Aug 8;38(1):2244696. doi: 10.1080/14756366.2023.2244696 (PMC10413920; doi:10.1080/14756366.2023.2244696)
Supplement: Supplemental Material [file IENZ_A_2244696_SM0383.pdf]

Supporting information

Design, synthesis and *in vitro* biological studies of novel triazoles with potent and broad-spectrum antifungal activity

Junhe Bao[a,†], Yumeng Hao[a,†], Tingjunhong Ni[b,†], Ruina Wang[c], Jiacun Liu[c], Xiaochen Chi[d], Ting Wang[a], Shichong Yu[a], Yongsheng Jin[a], Lan Yan[c], Xiaomei Li[e,*], Dazhi Zhang[a,*] and Fei Xie[a,*]

[a] *Department of Organic Chemistry, School of Pharmacy, Naval Medical University, 325 Guohe Road, Shanghai 200433(China);* [b] *Department of Pharmacy, Shanghai Tenth People's Hospital, School of Medicine, Tongji University, No.1239 Siping Road, Shanghai 200072(China);*[c] *Center of New Drug research, School of Pharmacy, Naval Medical University, 325 Guohe Road, Shanghai 200433(China);*[d] *School of Traditional Chinese Materia Medica, Shenyang Pharmaceutical University, 103 Wenhua Road, Shenyang 110016(China);* [e] *Department of Stomatology, Changhai Hospital, Naval Medical University, No. 168 Changhai Road, Shanghai 200433(China)*

†These authors contributed equally to this work.

The characterization data of all synthesized compounds are given below:

*N-((2R,3R)-3-(2,4-difluorophenyl)-3-hydroxy-4-(1H-1,2,4-triazol-1-yl)butan-2-yl)-3-phenylpropanamide (**A1**)*

White solid, yield 68%, ^{1}H NMR (300 MHz, DMSO-d_6) δ 8.18 (s, 1H, triazole ring), 7.84 (d, J = 8.9 Hz, 1H, –CONH–), 7.63 (s, 1H, triazole ring), 7.28 – 7.07 (m, 7H, Ar-H), 6.87 (td, J = 8.5, 2.7 Hz, 1H, Ar-H), 5.72 (s, 1H, OH), 4.59 (dd, J = 15.5, 6.4 Hz, 2H, –CH_2–), 4.12 (d, J = 14.5 Hz, 1H, –CH–), 2.96 – 2.81 (m, 2H, –CH_2–), 2.57 – 2.50 (m, 2H, –CH_2–), 0.74 (d, J = 6.9 Hz, 3H, CH_3). ^{13}C NMR (75 MHz, DMSO-d_6) δ 172.04, 163.89 – 160.63 (J_{CF} = 244 Hz), 160.53 – 157.42 (J_{CF} = 241 Hz), 157.26, 150.86, 141.63, 130.56 – 130.43 (J_{CF} = 9Hz), 128.80 (×2), 128.66 (×2), 126.31, 125.00 – 124.83 (J_{CF} = 13Hz), 111.36 – 111.08 (J_{CF} = 21 Hz), 104.66 – 104.29 (J_{CF} = 28 Hz), 77.75 – 77.67 (J_{CF} = 6 Hz), 55.54, 48.82 – 48.74 (J_{CF} =6 Hz), 37.39, 31.50, 15.74. LC-MS (ESI), m/z: $[M+1]^+$400.9, $C_{21}H_{22}F_2N_4O_2$.

*N-((2R,3R)-3-(2,4-difluorophenyl)-3-hydroxy-4-(1H-1,2,4-triazol-1-yl)butan-2-yl)-3-(2-fluorophenyl)propenamide (**A2**)*

White solid, yield 50%, ^{1}H NMR (300 MHz, DMSO-d_6) δ 8.17 (s, 1H, triazole ring), 7.87 (d, J = 8.7 Hz, 1H, –CONH–), 7.63 (s, 1H, triazole ring), 7.39 – 7.02 (m, 6H, Ar-H), 6.87 (td, J = 8.6, 2.8 Hz, 1H, Ar-H), 5.72 (s, 1H, OH), 4.71 – 4.51 (m, 2H, –CH_2–), 4.16 (d, J = 14.6 Hz, 1H, –CH–), 2.91 (td, J = 7.6, 3.7 Hz, 2H, –CH_2–), 2.65 – 2.51 (m, 2H, –CH_2–), 0.75 (d, J = 6.9 Hz, 3H, CH_3). ^{13}C NMR (75 MHz, DMSO-d_6) δ 171.71, 163.90 – 160.64 (J_{CF} = 245 Hz), 162.62 – 159.40 (J_{CF} = 241 Hz), 160.64 – 157.43 (J_{CF} = 241 Hz), 150.83, 144.90, 131.27 – 131.21 (J_{CF} = 5 Hz), 130.56 – 130.43 (J_{CF} = 10 Hz), 128.52 – 128.29 (J_{CF} = 17 Hz), 128.41 – 128.09 (J_{CF} = 24 Hz), 124.99 – 124.81 (J_{CF} = 14 Hz), 124.68 – 124.63 (J_{CF} = 4 Hz), 115.65 – 115.37 (J_{CF} = 21 Hz), 111.40 – 111.13 (J_{CF} = 21 Hz), 104.66 – 104.31 (J_{CF} = 26 Hz), 77.76 – 77.69 (J_{CF} = 5 Hz), 55.54 – 55.49 (J_{CF} = 4 Hz), 48.88 – 48.81 (J_{CF} = 5 Hz), 35.82, 24.77 – 24.73 (J_{CF} = 3 Hz), 15.73.

LC-MS (ESI), m/z: $[M+1]^+$418.9, $C_{21}H_{21}F_3N_4O_2$.

*N-((2R,3R)-3-(2,4-difluorophenyl)-3-hydroxy-4-(1H-1,2,4-triazol-1-yl)butan-2-yl)-3-(3-fluorophenyl)propenamide (**A3**)*

White solid, yield 70%, ^{1}H NMR (400 MHz, DMSO-d_6) δ 8.17 (s, 1H, triazole ring), 7.82 (d, J = 9.0 Hz, 1H, –CONH–), 7.63 (s, 1H, triazole ring), 7.25 (ddd, J = 14.6, 10.0, 6.9 Hz, 2H, Ar-H), 7.19 – 7.05 (m, 3H, Ar-H), 6.90 (m, 2H, Ar-H), 5.71 (s, 1H, OH), 4.62 (dd, J = 11.8, 5.7 Hz, 2H, –CH_2–), 4.17 (d, J = 14.4 Hz, 1H, –CH–), 2.92 (td, J = 7.4, 2.8 Hz, 2H, –CH_2–), 2.56 (td, J = 7.5, 5.0 Hz, 2H, –CH_2–), 0.75 (d, J = 6.9 Hz, 3H, CH_3).

^{13}C NMR (101 MHz, DMSO-d_6) δ 171.83, 163.86 – 161.44 (J_{CF} = 245 Hz),163.49 – 161.04 (J_{CF} = 247 Hz), 160.15 – 157.70 (J_{CF} = 247 Hz), 150.86, 144.88, 144.70 – 144.63 (J_{CF} = 7 Hz), 130.51 – 130.43 (J_{CF} =8 Hz), 124.99 – 124.96 (J_{CF} = 3 Hz), 124.92 – 124.79 (J_{CF} = 13 Hz), 115.59 – 115.38 (J_{CF} = 21 Hz), 113.18 – 112.97 (J_{CF} = 21 Hz), 111.3 – 111.16 (J_{CF} = 21 Hz), 111.13, 104.58 – 104.30 (J_{CF} = 28 Hz), 77.77 – 77.71 (J_{CF} = 6 Hz), 55.54 – 55.49 (J_C

$_F$ = 5 Hz), 48.85 – 48.79 (J_{CF} = 6 Hz), 36.95, 31.11 – 31.09 (J_{CF} = 2 Hz), 15.75. LC-MS (ESI), m/z: $[M+1]^+$418.9, $C_{21}H_{21}F_3N_4O_2$.

*N-((2R,3R)-3-(2,4-difluorophenyl)-3-hydroxy-4-(1H-1,2,4-triazol-1-yl)butan-2-yl)-3-(4-fluorophenyl)propenamide (**A4**)*

White solid, yield 68%,^{1}H NMR (400 MHz, DMSO-d_6) δ 8.17 (s, 1H, triazole ring), 7.81 (d, J = 9.0 Hz, 1H, –CONH–), 7.63 (s, 1H, triazole ring), 7.26 (ddd, J = 20.1, 8.9, 6.1 Hz, 3H, Ar-H), 7.14 (ddd, J = 12.0, 9.1, 2.7 Hz, 1H, Ar-H), 7.09 – 7.00 (m, 2H, Ar-H), 6.88 (td, J = 8.5, 2.8 Hz, 1H, Ar-H), 5.70 (s, 1H, OH), 4.60 (dd, J = 15.3, 5.8 Hz, 2H, –CH_2–), 4.12 (d, J = 14.5 Hz, 1H, –CH–), 2.96 – 2.80 (m, 2H, –CH_2–), 2.55 (dd, J = 14.6, 7.2 Hz, 2H, –CH_2–), 0.75 (d, J = 6.9 Hz, 3H, CH_3). ^{13}C NMR (101 MHz, DMSO-d_6) δ 171.91, 163.35 – 160.91 (J_{CF} = 246 Hz), 162.34 – 159.94 (J_{CF} = 242 Hz), 160.14 – 157.69 (J_{CF} = 247 Hz), 150.86, 144.89, 137.73 – 137.70 (J_{CF} = 3 Hz), 130.62 – 130.54 (J_{CF} = 8 Hz) (×2), 130.48 – 130.39 (J_{CF} = 9 Hz), 124.96 – 124.83 (J_{CF} = 13 Hz), 115.40 – 115.19 (J_{CF} = 21 Hz) (×2), 111.36 – 111.16 (J_{CF} = 20 Hz), 104.57 – 104.31 (J_{CF} = 26 Hz), 77.75 – 77.70 (J_{CF} = 5 Hz), 55.53 – 55.48 (J_{CF} = 5 Hz), 48.82 – 48.76 (J_{CF} = 6 Hz), 37.43, 30.64, 15.75. LC-MS (ESI), m/z: $[M+1]^+$418.9, $C_{21}H_{21}F_3N_4O_2$.

*3-(4-chlorophenyl)-N-((2R,3R)-3-(2,4-difluorophenyl)-3-hydroxy-4-(1H-1,2,4-triazol-1-yl)butan-2-yl)propenamide (**A5**)*

White solid, yield 72%,^{1}H NMR (300 MHz, DMSO-d_6) δ 8.12 (s, 1H, triazole ring), 7.79 (d, J = 9.2 Hz, 1H, –CONH–), 7.59 (s, 1H, triazole ring), 7.27 – 7.05 (m, 6H, Ar-H), 6.83 (td, J = 8.5, 2.8 Hz, 1H, Ar-H), 5.66 (d, J = 1.1 Hz, 1H, OH), 4.55 (dd, J = 15.3, 6.0 Hz, 2H, –CH_2–), 4.07 (d, J = 14.6 Hz, 1H, –CH–), 2.83 (t, J = 7.1 Hz, 2H, –CH_2–), 2.52 (dd, J = 11.4, 7.3 Hz, 2H, –CH_2–), 0.70 (d, J = 6.8 Hz, 3H, CH_3). ^{13}C NMR (75 MHz, DMSO-d_6) δ 171.81, 163.91 – 163.74 (J_{CF} = 13 Hz), 160.64 – 160.48 (J_{CF} = 12 Hz), 157.42 – 157.25 (J_{CF} = 12 Hz), 150.88, 140.64, 131.02, 130.72 (×2), 130.49 – 130.36 (J_{CF} = 10 Hz), 128.56 (×2), 124.98 – 124.80 (J_{CF} = 14 Hz), 111.39 – 111.12 (J_{CF} = 20 Hz),104.66 – 104.31 (J_{CF} = 26 Hz), 77.77 – 77.69 (J_{CF} = 6 Hz), 55.53 – 55.47 (J_{CF} = 5 Hz), 48.84 – 48.77 (J_{CF} = 5 Hz), 37.13, 30.76, 15.76. LC-MS (ESI), m/z: $[M+1]^+$434.8, $C_{21}H_{21}F_2N_4O_2$.

*N-((2R,3R)-3-(2,4-difluorophenyl)-3-hydroxy-4-(1H-1,2,4-triazol-1-yl)butan-2-yl)-3-(p-tolyl)propenamide (**A6**)*

White solid, yield 75%, ^{1}H NMR (400 MHz, DMSO-d_6) δ 8.13 (s, 1H, triazole ring), 7.79 (d, J = 9.0 Hz, 1H, –CONH–), 7.63 (s, 1H, triazole ring), 7.24 (td, J = 9.1, 6.8 Hz, 1H, Ar-H), 7.18-7.08 (m, 3H, Ar-H), 7.03 (d, J = 8.0 Hz, 2H, Ar-H), 6.87 (td, J =8.5, 2.8 Hz, 1H, Ar-H), 5.68 (s, 1H, OH), 4.66-4.48 (m, 2H, –CH_2–), 4.00 (d, J = 14.5 Hz, 1H, –CH–), 2.84 (td, J = 7.4, 7.0, 3.0 Hz, 2H, –CH_2–), 2.54-2.46 (m, 2H, –CH_2–), 2.12 (s, 3H, CH_3), 0.75 (d, J = 7.0 Hz, 3H, CH_3). ^{13}C NMR (101 MHz, DMSO-d_6) δ 172.02, 163.47 – 161.02 (J_{CF} = 247 Hz), 160.25 – 157.79 (J_{CF} = 248 Hz), 150.82, 144.86, 138.41, 135.25, 130.55 – 130.45 (J_{CF} = 10 Hz), 129.19 (×2), 128.75 (×2), 124.96 – 124.83 (J_{CF} = 13 Hz), 111.34 – 111.14 (J_{CF} = 20 Hz), 104.57 – 104.29 (J_{CF} = 28

Hz), 77.69 – 77.64 (J_{CF} = 5 Hz), 55.45 – 55.40 (J_{CF} = 5 Hz), 48.78 – 48.72 (J_{CF} = 6 Hz), 37.52, 31.13, 20.88, 15.73. LC-MS (ESI), m/z: $[M+1]^+$414.9, $C_{22}H_{24}F_2N_4O_2$.

*N-((2R,3R)-3-(2,4-difluorophenyl)-3-hydroxy-4-(1H-1,2,4-triazol-1-yl)butan-2-yl)-3-(4-methoxy-phenyl)propenamide (**A7**)*

White solid, yield 75%,^{1}H NMR (300 MHz, DMSO-d_6) δ 8.14 (s, 1H, triazole ring), 7.80 (d, *J* = 9.0 Hz, 1H, –CONH–), 7.62 (s, 1H, triazole ring), 7.29 – 7.08 (m, 4H, Ar-H), 6.87 (td, *J* = 8.4, 2.7 Hz, 1H, Ar-H), 6.81 – 6.74 (m, 2 H, Ar-H), 5.69 (s, 1H, OH), 4.67 – 4.49 (m, 2H, –CH_2–), 4.03 (d, *J* = 14.5 Hz, 1H, –CH–), 3.59 (s, 3H, OCH_3), 2.93 – 2.74 (m, 2H, –CH_2–), 2.51 (t, *J* = 6.7 Hz, 2H, –CH_2–), 0.75 (d, *J* = 6.9 Hz, 3H, CH_3). ^{13}C NMR (75 MHz, D MSO-d_6) δ 172.08, 163.71 – 160.46 (J_{CF} = 244 Hz), 157.97, 160.46 – 157.25 (J_{CF} = 241 Hz), 150.82, 144.85, 133.38, 130.56 – 130.44 (J_{CF} = 9 Hz), 129.79 (×2), 124.95 – 124.78 (J_{CF} = 13 Hz), 114.03 (×2), 111.34 – 111.11 (J_{CF} = 18 Hz), 104.65 – 104.30 (J_{CF} = 26 Hz), 77.73 – 77.66 (J_{CF} = 5 Hz), 55.52 – 55. 46 (J_{CF} = 5 Hz), 55.27, 48.79 – 48.72 (J_{CF} = 5 Hz), 37.73, 30.68, 15.73. LC-MS (ESI), m/z: $[M+1]^+$430.9, $C_{21}H_{22}F_2N_4O_2$.

*3-(4-cyanophenyl)-N-((2R,3R)-3-(2,4-difluorophenyl)-3-hydroxy-4-(1H-1,2,4-triazol-1-yl)butan-2-yl)propenamide (**A8**)*

White solid, yield 80%,^{1}H NMR (400 MHz, DMSO-d_6) δ 8.17 (s, 1H, triazole ring), 7.84 (d, *J* = 9.0 Hz, 1H, –CONH–), 7.75 – 7.69 (m, 2H, Ar-H), 7.64 (s, 1H, triazole ring), 7.47 (d, *J* = 8.4 Hz, 2H, Ar-H), 7.25 (td, *J* = 9.1, 6.9 Hz, 1H, Ar-H), 7.14 (ddd, *J* = 11.9, 9.1, 2.6 Hz, 1H, Ar-H), 6.88 (td, *J* = 8.5, 2. 8 Hz, 1H, Ar-H), 5.70 (s, 1H, OH), 4.60 (dd, *J* = 15.5, 5.9 Hz, 2H, –CH_2–), 4.13 (d, *J* = 14.4 Hz, 1H, –CH–), 2.97 (d, *J* = 7.4 Hz, 2H, –CH_2–), 2.59 (td, *J* = 7.5, 5.2 Hz, 2H, –CH_2–), 0.75 (d, *J* = 6.9 Hz, 3H, CH_3). ^{13}C NMR (101 MHz, DMSO-d_6) δ 171.59, 163.49 – 161.04 (J_{CF} = 247 Hz), 160.25 – 157.80 (J_{CF} = 247 Hz), 150.89, 147.81, 144.85, 132.56 (×2), 130.54 – 130.45 (J_{CF} = 9 Hz), 130.00 (×2), 124.93 – 124.80 (J_{CF} = 13 Hz), 119.31, 111.38 – 111.17 (J_{CF} = 21 Hz), 109.29, 104.58 – 104.30 (J_{CF} = 28 Hz), 77.76 – 77.70 (J_{CF} = 6 Hz), 55.50 – 55.46 (J_{CF} = 4 Hz), 48.84 – 48.78 (J_{CF} = 36 Hz), 36.55, 31.47, 15.76. LC-MS (ESI), m/z: $[M+1]^+$425.9, $C_{22}H_{21}F_2N_5O_2$.

*N-((2R,3R)-3-(2,4-difluorophenyl)-3-hydroxy-4-(1H-1,2,4-triazol-1-yl)butan-2-yl)-3-(4-(tert-bu-tyl)phenyl)-propanamide (**A9**)*

White solid, yield 68%, ^{1}H NMR (400 MHz, DMSO-d_6) δ 8.16 (s, 1H, triazol e ring), 7.80 (d, *J* = 9.0 Hz, 1H, –CONH–), 7.62 (s, 1H, triazole ring), 7.25 (d, *J* = 8.1 Hz, 3H, Ar-H), 7.20 – 7.08 (m, 3H, Ar-H), 6.87 (td, *J* = 8.4, 2.6 Hz, 1H, Ar-H), 5.73 (s, 1H, OH), 4.66 – 4.53 (m, 2H, –CH_2–), 4.12 (d, *J* = 14.5 Hz, 1H, –CH–), 2.85 (d, *J* = 7.1, 6.6 Hz, 2H, –CH_2–), 2.62 – 2.51 (m, 2H, –CH_2–), 1.17 (s, 9H, -$C(CH_3)_3$), 0.75 (d, *J* = 6.9 Hz, 3H, CH_3). ^{13}C NMR (101 MHz, DMSO-d_6) δ 172.13, 163.47 – 161.03 (J_{CF} = 247 Hz), 160.13 – 1 57.68 (J_{CF} = 247 Hz), 150.81, 148.62, 144.86, 138.48, 130.56 – 130.47 (J_{CF} = 9 Hz), 128.47 (×2), 125.35 (×2), 124.96 – 124.83 (J_{CF} = 13 Hz), 111.33 – 11 1.12 (J_{CF} = 21 Hz), 104.57 – 104.31 (J_{CF} = 26 Hz), 77.73 – 77.68 (J_{CF} = 5 H

z), 55.58 – 55.54 (J_{CF} = 4 Hz), 48.87 – 48.81 (J_{CF} = 6 Hz), 37.42, 34.41, 31.53 (×3), 30.97, 15.72.LC-MS (ESI), m/z: $[M+1]^+$456.9, $C_{25}H_{30}F_2N_4O_2$.

*N-((2R,3R)-3-(2,4-difluorophenyl)-3-hydroxy-4-(1H-1,2,4-triazol-1-yl)butan-2-yl)-3-(4-(trifluoromethyl)phenyl)propenamide (**A10**)*

White solid, yield 60%, ^{1}H NMR (300 MHz, DMSO-d_6) δ 8.16 (s, 1H, triazole ring), 7.86 (d, J = 8.9 Hz, 1H, –CONH–), 7.61 (d, J = 8.9 Hz, 3H, Ar-H), 7.48 (d, J = 8.3 Hz, 2H, Ar-H), 7.30 – 7.07 (m, 2H, Ar-H), 6.87 (td, J = 8.5, 2.7 Hz, 1H, Ar-H), 5.70 (s, 1H, OH), 4.59 (dd, J = 15.4, 6.7 Hz, 2H, –CH_2–), 4.12 (d, J = 14.5 Hz, 1H, –CH–), 3.06 – 2.90 (m, 2H, –CH_2–), 2.59 (td, J = 7.5, 3.3 Hz, 2H, –CH_2–), 0.74 (d, J = 6.9 Hz, 3H, CH_3). ^{13}C NMR (75 MHz, DMSO-d_6) δ 171.69, 171.10, 163.74 – 160.64 (J_{CF} = 233 Hz), 160.48 – 157.41 (J_{CF} = 230 Hz), 157.24, 150.83, 146.67, 144.82, 130.47 – 130.34 (J_{CF} = 10 Hz), 129.68, 127.43 – 126.62 (J_{CF} = 61 Hz), 125.54 – 125.45 (J_{CF} = 7 Hz), 124.96 – 124.78 (J_{CF} = 14 Hz), 123.01, 111.40 – 111.13 (J_{CF} = 20 Hz), 104.66 – 104.31(J_{CF} = 26 Hz), 77.77-77.69 (J_{CF} = 6 Hz), 55.46, 48.85 – 48.77 (J_{CF} = 6 Hz), 36.82, 31.21, 15.74. LC-MS (ESI), m/z: $[M+1]^+$468.9, $C_{22}H_{21}F_5N_4O_2$.

*N-((2R,3R)-3-(2,4-difluorophenyl)-3-hydroxy-4-(1H-1,2,4-triazol-1-yl)butan-2-yl)-3-(4-acetylphenyl)-propanamide (**A11**)*

White solid, yield 80%, ^{1}H NMR (400 MHz, DMSO-d_6) δ 8.18 (s, 1H, triazole ring), 7.90 (d, J = 9.0 Hz, 1H, –CONH–), 7.84 (d, J = 8.3 Hz, 2H, Ar-H), 7.64 (s, 1H, triazole ring), 7.41 (d, J = 8.3 Hz, 2H, Ar-H), 7.23 (td, J = 9.1, 6.8 Hz, 1H, Ar-H), 7.13 (ddd, J = 12.0, 9.1, 2.7 Hz, 1H, Ar-H), 6.87 (td, J = 8.4, 2.8 Hz, 1H, Ar-H), 5.74 (s, 1H, OH), 4.66 – 4.50 (m, 2H, –CH_2–), 4.07 (d, J = 14.5 Hz, 1H, –CH–), 2.97 (dt, J = 9.9, 5.5 Hz, 2H, –CH_2–), 2.69 – 2.52 (m, 2H, –CH_2–), 2.44 (s, 3H, $COCH_3$), 0.75 (d, J = 6.9 Hz, 3H, CH_3). ^{13}C NMR (101 MHz, DMSO-d_6) δ 197.71, 171.69, 163.48 – 161.03 (J_{CF} = 247 Hz), 160.24 – 157.79 (J_{CF} = 247 Hz), 150.65, 147.43, 144.78, 135.29, 130.48 – 130.38 (J_{CF} = 10 Hz), 129.21 (×2), 128.67 (×2), 124.91 – 124.78 (J_{CF} = 13 Hz), 111.33 – 111.13 (J_{CF} = 20 Hz), 104.3 – 104.04 (J_{CF} = 26 Hz), 77.65 – 77.60 (J_{CF} = 5 Hz), 55.48 – 55.44 (J_{CF} = 4 Hz), 48.82 – 48.77 (J_{CF} = 5 Hz), 36.87, 31.44, 26.90, 15.75. LC-MS (ESI), m/z: $[M+1]^+$442.9, $C_{23}H_{24}F_2N_4O_3$.

*N-((2R,3R)-3-(2,4-difluorophenyl)-3-hydroxy-4-(1H-1,2,4-triazol-1-yl)butan-2-yl)-3-(m-tolyl)propenamide (**A12**)*

White solid, yield 68%, ^{1}H NMR (300 MHz, DMSO-d_6) δ 8.14 (s, 1H, triazole ring), 7.81 (d, J = 9.0 Hz, 1H, –CONH–), 7.63 (s, 1H, triazole ring), 7.31 – 6.99 (m, 5H, Ar-H), 6.87 (dd, J = 10.2, 7.2 Hz, 2H, Ar-H), 5.69 (s, 1H, OH), 4.69 – 4.52 (m, 2H, –CH_2–), 4.08 (d, J = 14.5 Hz, 1H, –CH–), 2.86 (dt, J = 10.5, 7.1, 3.3 Hz, 2H, –CH_2–), 2.56 – 2.48 (m, 2H, –CH_2–), 2.19 (s, 3H, CH_3), 0.75 (d, J = 6.9 Hz, 3H, CH_3). ^{13}C NMR (75 MHz, DMSO-d_6) δ 172.04, 163.74 – 160.47 (J_{CF} = 245 Hz), 160.64 – 157.26 (J_{CF} = 254 Hz), 150.82, 144.87, 141.52, 137.63, 130.57 – 130.49 (J_{CF} = 6 Hz), 129.52, 128.56, 126.94, 125.87, 124.99 – 124.82 (J_{CF} = 13 Hz), 111.39 – 111.08 (J_{CF} = 23 Hz), 104.66 – 104.29 (J_{CF} = 28 Hz), 77.74 – 77.67 (J_{CF} = 5 Hz), 55.47, 48.81 – 48.73

(J_{CF} = 6 Hz), 37.44, 31.43, 21.35, 15.73. LC-MS (ESI), m/z:, [M+1]$^+$414.9, $C_{22}H_{24}F_2N_4O_2$.

*N-((2R,3R)-3-(2,4-difluorophenyl)-3-hydroxy-4-(1H-1,2,4-triazol-1-yl)butan-2-yl)-3-(4-fluoro-3-methylphenyl)propenamide (**A13**)*

White solid, yield 80%, ^{1}H NMR (300 MHz, DMSO-d_6) δ 8.14 (s, 1H, triazole ring), 7.81 (d, J = 9.1 Hz, 1H, –CONH–), 7.63 (s, 1H, triazole ring), 7.30 – 7.20 (m, 1H, Ar-H), 7.15 (ddd, J = 9.6, 7.0, 3.9 Hz, 2H, Ar-H), 7.08 (ddd, J = 7.9, 5.1, 2.5 Hz, 1H, Ar-H), 6.97 (t, J = 9.1 Hz, 1H, Ar-H), 6.87 (td, J = 8.5, 2.8 Hz, 1H, Ar-H), 5.69 (s, 1H, OH), 4.68 – 4.51 (m, 2H, –CH_2–), 4.05 (d, J = 14.5 Hz, 1H, –CH–), 2.94 – 2.70 (m, 2H, –CH_2–), 2.55 – 2.48 (m, 2H, –CH_2–), 2.10 (d, J = 2.1 Hz, 3H, CH_3), 0.74 (d, J = 6.9 Hz, 3H, CH_3). ^{13}C NMR (75 MHz, DMSO-d_6) δ 171.93, 163.90 – 160.66 (J_{CF} = 243 Hz), 161.23 – 158.04 (J_{CF} = 240 Hz), 160.49 – 157.24 (J_{CF} = 244 Hz), 150.84, 144.82, 137.37 – 137.33 (J_{CF} = 3 Hz), 131.99 – 131.92 (J_{CF} = 5 Hz), 130.57 – 130.44 (J_{CF} = 10 Hz), 127.89 – 127.79 (J_{CF} = 8 Hz), 124.95 – 124.77 (J_{CF} = 14 Hz), 124.19 – 123.96 (J_{CF} = 17 Hz), 115.10 – 114.82 (J_{CF} = 21 Hz), 111.40 – 111.12 (J_{CF} = 21 Hz), 104.66 – 104.32 (J_{CF} = 26 Hz), 77.74 – 77.66 (J_{CF} = 6 Hz), 55.50 – 55.44 (J_{CF} = 5 Hz), 48.79 – 48.71 (J_{CF} = 6 Hz), 37.48, 30.64, 15.72, 14.47 – 14.43 (J_{CF} = 3 Hz). LC-MS (ESI), m/z:[M+1]$^+$432.9, $C_{22}H_{23}F_3N_4O_2$.

*N-((2R,3R)-3-(2,4-difluorophenyl)-3-hydroxy-4-(1H-1,2,4-triazol-1-yl)butan-2-yl)-3-(3,4-difluoro-phenyl)-propanamide (**A14**)*

White solid, yield 85%, ^{1}H NMR (300 MHz, DMSO-d_6) δ 8.16 (s, 1H, triazole ring), 7.83 (d, J = 9.0 Hz, 1H, –CONH–), 7.63 (s, 1H, triazole ring), 7.38 – 7.03 (m, 5H, Ar-H), 6.87 (td, J = 8.5, 2.8 Hz, 1H, Ar-H), 5.71 (s, 1H, OH), 4.69 – 4.54 (m, 2H, –CH_2–), 4.13 (d, J = 14.5 Hz, 1H, –CH–), 2.99 – 2.83 (m, 2H, –CH_2–), 2.63 – 2.52 (m, 2H, –CH_2–), 0.74 (d, J = 6.9 Hz, 3H, CH_3). ^{13}C NMR (75 MHz, DMSO-d_6) δ 171.71, 163.91 – 160.68 (J_{CF} = 242 Hz), 160.49 – 157.25 (J_{CF} = 243 Hz), 151.31 – 148.07 (J_{CF} = 243 Hz), 150.87, 150.02 – 146.79 (J_{CF} = 242 Hz), 146.63, 144.83, 139.53 – 139.45 (J_{CF} = 6 Hz), 130.48 – 130.36 (J_{CF} = 9 Hz), 125.62 – 125.54 (J_{CF} = 6 Hz), 124.95 – 124.77 (J_{CF} = 14 Hz),117.79 – 117.57 (J_{CF} = 17 Hz), 111.37 – 111.14 (J_{CF} = 17 Hz), 104.67 – 104.33 (J_{CF} = 26 Hz), 77.76 – 77.69 (J_{CF} = 5 Hz), 55.51, 48.82 – 48.75 (J_{CF} = 5 Hz), 36.99, 30.54, 15.74. LC-MS (ESI), m/z: [M+1]$^+$436.9, $C_{21}H_{20}F_4N_4O_2$.

*N-((2R,3R)-3-(2,4-difluorophenyl)-3-hydroxy-4-(1H-1,2,4-triazol-1-yl)butan-2-yl)-3-(4-chloro-3-fluorophenyl)-propanamide (**A15**)*

White solid, yield 75%, ^{1}H NMR (300 MHz, DMSO-d_6) δ 8.16 (s, 1H, triazole ring), 7.84 (d, J = 8.9 Hz, 1H, –CONH–), 7.63 (s, 1H, triazole ring), 7.43 (t, J = 8.1 Hz, 1H, Ar-H), 7.36 – 7.07 (m, 4H, Ar-H), 6.87 (td, J = 8.5, 2.8 Hz, 1H, Ar-H), 5.71 (s, 1H, OH), 4.60 (dd, J = 15.0, 5.4 Hz, 2H, –CH_2–), 4.12 (d, J = 14.5 Hz, 1H, –CH–), 2.90 (t, J = 7.0 Hz, 2H, –CH_2–), 2.56 (dt, J = 7.4, 3.6 Hz, 2H, –CH_2–), 0.74 (d, J = 6.9 Hz, 3H, CH_3). ^{13}C NMR (75 MHz, DMSO-d_6) δ 171.64, 163.91 – 160.65 (J_{CF} = 245 Hz), 159.09 – 155.83 (J

$_{CF}$ = 245 Hz), 160.65 – 157.41 (J_{CF} = 243 Hz), 150.87, 144.83, 143.66 – 143.57 (J_{CF} = 7 Hz), 130.64, 130.56 – 130.43 (J_{CF} = 10 Hz), 126.31 – 126.26 (J_{CF} = 4 Hz), 124.94 – 124.76 (J_{CF} = 14 Hz), 117.36 – 117.13 (J_{CF} = 18 Hz), 117.32 – 117.05 (J_{CF} = 20 Hz), 111.36 – 111.09 (J_{CF} = 20 Hz), 104.67 – 104.33 (J_{CF} = 26 Hz), 77.76 – 77.69 (J_{CF} = 5 Hz), 55.50 – 55.45 (J_{CF} = 4 Hz), 48.83 – 48.76 (J_{CF} = 5 Hz), 36.74, 30.68, 15.75. LC-MS (ESI), m/z: $[M+1]^+$452.8, $C_{21}H_{20}ClF_3N_4O_2$.

*N-((2R,3R)-3-(2,4-difluorophenyl)-3-hydroxy-4-(1H-1,2,4-triazol-1-yl)butan-2-yl)-3-(3,4-dichlorophenyl)-propanamide (**A16**)*

White solid, yield 75%, ^{1}H NMR (300 MHz, DMSO-d_6) δ 8.15 (s, 1H, triazole ring), 7.86 (d, J = 8.7 Hz, 1H, –CONH–), 7.63 (s, 1H, triazole ring), 7.56 – 7.45 (m, 2H, Ar-H), 7.30 – 7.18 (m, 2H, Ar-H), 7.18 – 7.08 (m, 1H, Ar-H), 6.87 (td, J = 8.6, 2.8 Hz, 1H, Ar-H), 5.72 (s, 1H, OH), 4.59 (dd, J = 15.1, 8.5 Hz, 2H, –CH_2–), 4.10 (d, J = 14.5 Hz, 1H, –CH–), 2.89 (td, J = 7.2, 6.8, 2.4 Hz, 2H, –CH_2–), 2.63 – 2.50 (m, 2H, –CH_2–), 0.74 (d, J = 6.9 Hz, 3H, CH_3). ^{13}C NMR (75 MHz, DMSO-d_6) δ 171.60, 163.74 – 160.64 (J_{CF} = 233 Hz), 160.48 – 157.24 (J_{CF} = 243 Hz), 150.86, 144.83, 142.94, 131.17, 130.88, 130.72, 130.54 – 130.43 (J_{CF} = 8 Hz), 129.44, 128.98, 124.93 – 124.76 (J_{CF} = 13 Hz), 111.40 – 111.13 (J_{CF} = 20 Hz), 104.68 – 104.33 (J_{CF} = 26 Hz), 77.73 – 77.66 (J_{CF} = 5 Hz), 55.47, 48.79 – 48.72 (J_{CF} = 5 Hz), 36.77, 30.41, 15.77. LC-MS (ESI), m/z: $[M+1]^+$470.1, $C_{21}H_{20}Cl_2F_2N_4O_2$.

*3-(3,5-difluorophenyl)-N-((2R,3R)-3-(2,4-difluorophenyl)-3-hydroxy-4-(1H-1,2,4-triazol-1-yl)butan-2-yl)propenamide (**A17**)*

White solid, yield 68%, ^{1}H NMR (300 MHz, DMSO-d_6) δ 8.18 (s, 1H, triazole ring), 7.85 (d, J = 9.0 Hz, 1H, –CONH–), 7.63 (s, 1H, triazole ring), 7.32 – 7.07 (m, 2H, Ar-H), 7.06 – 6.81 (m, 4H, Ar-H), 5.73 (s, 1H, OH), 4.69 – 4.53 (m, 2H, –CH_2–), 4.20 (d, J = 14.5 Hz, 1H, –CH–), 3.00 – 2.84 (m, 2H, –CH_2–), 2.57 (t, J = 7.8 Hz, 2H, –CH_2–), 0.75 (d, J = 6.9 Hz, 3H, CH_3). ^{13}C NMR (75 MHz, DMSO-d_6) δ 171.63, 164.45 – 161.20 (J_{CF} = 244 Hz) (×2), 163.92 – 160.66 (J_{CF} = 245 Hz), 160.54 – 157.27 (J_{CF} = 245 Hz), 150.86, 146.55 – 146.43 (J_{CF} = 9 Hz), 144.85, 130.56 – 130.43 (J_{CF} = 10 Hz), 124.95 – 124.77 (J_{CF} = 14 Hz), 112.09 – 111.77 (J_{CF} = 24 Hz) (×2), 111.41 – 111.14 (J_{CF} = 20 Hz), 104.67 – 104.30 (J_{CF} = 28 Hz), 102.11 – 101.76 (J_{CF} = 26 Hz), 77.77 – 77.70 (J_{CF} = 5 Hz), 55.54 – 55.47 (J_{CF} = 5 Hz), 48.85 – 48.77 (J_{CF} = 6 Hz), 36.51, 31.01, 15.75. LC-MS (ESI), m/z: $[M+1]^+$436.8, $C_{21}H_{20}F_4N_4O_2$.

*N-((2R,3R)-3-(2,4-difluorophenyl)-3-hydroxy-4-(1H-1,2,4-triazol-1-yl)butan-2-yl)-3-(pyridin-2-yl)propenamide (**A18**)*

White solid, yield 55%, ^{1}H NMR (400 MHz, DMSO-d_6) δ 8.49 – 8.41 (m, 1H, pyridine ring), 8.20 (s, 1H, triazole ring), 7.87 (d, J = 8.9 Hz, 1H, –CONH–), 7.67 (td, J = 7.6, 1.9 Hz, 1H, pyridine ring), 7.63 (s, 1H, triazole ring), 7.33 – 7.20 (m, 2H, Ar-H), 7.20 – 7.09 (m, 2H, pyridine ring), 6.88 (td, J = 8.4, 2.6 Hz, 1H, Ar-H), 5.75 (s, 1H, OH), 4.68 – 4.56 (m, 2H, –CH_2–), 4.29 (d, J = 14.5 Hz, 1H, –CH–), 3.02 (t, J = 3.3 Hz, 2H, –CH_2–), 2.77 – 2.57 (m, 2H, –CH_2–), 0.77 (d, J = 6.9 Hz, 3H, CH_3). ^{13}C NMR (101 MHz, DMSO

-d_6) δ 172.19, 163.46 – 161.02(J_{CF} = 246 Hz), 160.96, 160.29 – 157.83 (J_{CF} = 248 Hz), 150.84, 149.28, 144.90, 136.83, 130.55 – 130.45 (J_{CF} = 10 Hz), 125.00 – 124.87 (J_{CF} = 13 Hz), 123.34, 121.77, 111.35 – 111.14 (J_{CF} = 21 Hz), 104.58 – 104.30 (J_{CF} = 28 Hz), 77.85 – 77.79 (J_{CF} = 6 Hz), 55.59, 48.81 – 48.75 (J_{CF} = 6 Hz), 35.12, 33.50, 15.73. LC-MS (ESI), m/z: $[M+1]^+$401.9, $C_{20}H_{21}F_2N_5O_2$.

*N-((2R,3R)-3-(2,4-difluorophenyl)-3-hydroxy-4-(1H-1,2,4-triazol-1-yl)butan-2-yl)-3-(3-methylpyridin-2-yl)propenamide (**A19**)*

White solid, yield 60%, ^{1}H NMR (400 MHz, DMSO-d_6) δ 8.25 (dd, J = 4.9, 1.9 Hz, 1H, pyridine ring), 8.21 (s, 1H, triazole ring), 7.89 (d, J = 9.0 Hz, 1H, –CONH–), 7.64 (s, 1H, triazole ring), 7.49 (dd, J = 7.6, 2.0 Hz, 1H, pyridine ring), 7.27 (td, J = 9.1, 6.9 Hz, 1H, Ar-H), 7.15 (ddd, J = 11.9, 9.1, 2.6 Hz, 1H, Ar-H), 7.07 (dd, J = 7.5, 4.8 Hz, 1H, pyridine ring), 6.89 (td, J = 8.5, 2.7 Hz, 1H, Ar-H), 5.77 (s, 1H, OH), 4.69 – 4.57 (m, 2H, –CH_2–), 4.39 (d, J = 14.5 Hz, 1H, –CH–), 3.13 – 2.92 (m, 2H, –CH_2–), 2.75 (dt, J = 14.9, 7.5 Hz, 1H, –CH_2–), 2.64 (ddd, J = 14.6, 7.8, 6.4 Hz, 1H, –CH_2–), 2.30 (s, 3H, CH_3), 0.79 (d, J = 6.9 Hz, 3H, CH_3). ^{13}C NMR (101 MHz, DMSO-d_6) δ 172.64, 163.46 – 161.02 (J_{CF} = 246 Hz), 160.30 – 157.84 (J_{CF} = 248 Hz), 159.02, 150.85, 146.41, 144.90, 137.57, 131.50, 130.51 – 130.41 (J_{CF} = 10 Hz), 125.05 – 124.92 (J_{CF} = 13 Hz), 121.71, 111.38 – 111.17 (J_{CF} = 21 Hz), 104.59 – 104.32 (J_{CF} = 27 Hz), 77.94 – 77.88 (J_{CF} = 6 Hz), 55.64 – 55.59 (J_{CF} = 5 Hz), 48.81 – 48.75 (J_{CF} = 6 Hz), 33.59, 30.14, 18.66, 15.71. LC-MS (ESI), m/z: $[M+1]^+$415.9, $C_{21}H_{23}F_2N_5O_2$.

*N-((2R,3R)-3-(2,4-difluorophenyl)-3-hydroxy-4-(1H-1,2,4-triazol-1-yl)butan-2-yl)-3-(4-methylpyridin-2-yl)propenamide (**A20**)*

White solid, yield 53%, ^{1}H NMR (400 MHz, DMSO-d_6) δ 8.30 (d, J = 5.1 Hz, 1H, pyridine ring), 8.19 (s, 1H, triazole ring), 7.87 (d, J = 9.0 Hz, 1H, –CONH–), 7.64 (s, 1H, triazole ring), 7.25 (td, J = 9.0, 6.8 Hz, 1H, Ar-H), 7.20 – 7.13 (m, 1H, Ar-H), 7.11 (d, J = 2.1 Hz, 1H, pyridine ring), 6.96 (dd, J = 5.1, 1.7 Hz, 1H, pyridine ring), 6.88 (td, J = 8.5, 2.8 Hz, 1H, Ar-H), 5.74 (s, 1H, OH), 4.76 – 4.56 (m, 2H, –CH_2–), 4.23 (d, J = 14.4 Hz, 1H, –CH–), 3.00 (m, 2H, –CH_2–), 2.75 – 2.55 (m, 2H, –CH_2–), 2.20 (s, 3H, CH_3), 0.76 (d, J = 6.9 Hz, 3H, CH_3). ^{13}C NMR (101 MHz, DMSO-d_6) δ 172.19, 163.46 – 161.02 (J_{CF} = 246 Hz), 160.73, 160.28 – 157.83 (J_{CF} = 47 Hz), 150.84, 149.07, 147.31, 144.89, 130.49 – 130.39 (J_{CF} = 10 Hz), 124.98 – 124.85 (J_{CF} = 13 Hz), 124.05, 122.62, 111.37 – 111.17 (J_{CF} = 20 Hz), 104.58 – 104.31 (J_{CF} = 27 Hz), 77.81 – 77.76 (J_{CF} = 5 Hz), 55.57 – 55.53 (J_{CF} = 4 Hz), 48.78 – 48.72 (J_{CF}=6Hz), 35.20, 33.40, 20.76, 15.71. LC-MS (ESI), m/z: $[M+1]^+$415.9, $C_{21}H_{23}F_2N_5O_2$.

*N-((2R,3R)-3-(2,4-difluorophenyl)-3-hydroxy-4-(1H-1,2,4-triazol-1-yl)butan-2-yl)-3-(5-methylpyridin-2-yl)propenamide (**A21**)*

White solid, yield 60%, ^{1}H NMR (400 MHz, DMSO-d_6) δ 8.27 (d, J = 2.5 Hz, 1H, pyridine ring), 8.21 (s, 1H, triazole ring), 8.03 (d, J = 9.0 Hz, 1H, –CONH–), 7.62 (s, 1H, triazole ring), 7.45 (dd, J = 8.1, 2.7 Hz, 1H, pyridine rin

g), 7.25 (td, J = 9.0, 6.8 Hz, 1H, pyridine ring), 7.20 – 7.08 (m, 2H, Ar-H), 6.87 (td, J = 8.5, 2.8 Hz, 1H, Ar-H), 6.05 (s, 1H, OH), 4.67 – 4.52 (m, 2H, $–CH_2–$), 4.20 (d, J = 14.5 Hz, 1H, –CH–), 3.08 – 2.90 (m, 2H, $–CH_2–$), 2.75 – 2.53 (m, 2H, $–CH_2–$), 2.15 (s, 3H, CH_3), 0.76 (d, J = 6.9 Hz, 3H, CH_3). ^{13}C NMR (101 MHz, DMSO-d_6) δ 172.14, 163.43 – 160.99 (J_{CF} = 246 Hz), 160.28 – 157.83 (J_{CF} = 247 Hz), 157.98, 150.79, 149.46, 144.91, 137.17, 130.59, 130.52 – 130.43 (J_{CF} = 9 Hz), 125.08 – 124.95 (J_{CF} = 13 Hz), 122.80, 111.33 – 111.13 (J_{CF} = 20 Hz), 104.29 – 104.01 (J_{CF} = 28 Hz), 77.66 – 77.61 (J_{CF} = 5 Hz), 55.47 – 55.43 (J_{CF} = 4 Hz), 48.77 – 48.71 (J_{CF} = 6 Hz), 35.34, 33.16, 17.84, 15.74. LC-MS (ESI), m/z: $[M+1]^+$415.9, $C_{21}H_{23}F_2N_5O_2$.

*N-((2R,3R)-3-(2,4-difluorophenyl)-3-hydroxy-4-(1H-1,2,4-triazol-1-yl)butan-2-yl)-3-(pyridin-3-yl)propenamide (**A22**)*

White solid, yield 55%, ^{1}H NMR (400 MHz, DMSO-d_6) δ 8.49 (d, J = 2.6 Hz, 1H, pyridine ring), 8.34 (dd, J = 4.8, 1.7 Hz, 1H, pyridine ring), 8.18 (s, 1H, triazole ring), 7.85 (d, J = 8.7 Hz, 1H, –CONH–), 7.68 (dt, J = 7.8, 2.0 Hz, 1H, pyridine ring), 7.63 (s, 1H, triazole ring), 7.32 – 7.11 (m, 3H, Ar-H), 6.88 (td, J = 8.6, 2.8 Hz, 1H, Ar-H), 5.72 (s, 1H, OH), 4.67 – 4.55 (m, 2H, $–CH_2–$), 4.15 (d, J = 14.4 Hz, 1H, –CH–), 2.99 – 2.84 (m, 2H, $–CH_2–$), 2.58 (td, J = 7.5, 3.9 Hz, 2H, $–CH_2–$), 0.74 (d, J = 7.0 Hz, 3H, CH_3). ^{13}C NMR (101 MHz, DMSO-d_6) δ 171.73, 163.48 – 161.03 (J_{CF} = 247 Hz), 160.27 – 157.82 (J_{CF} = 247 Hz), 150.86, 150.10, 147.65, 144.92, 137.05, 136.42, 130.52 – 130.42 (J_{CF} = 10 Hz), 124.95 – 124.82 (J_{CF} = 13 Hz),123.79, 111.38 – 111.17 (J_{CF} = 21 Hz), 104.59 – 104.31 (J_{CF} = 28 Hz), 77.75 – 77.69 (J_{CF} = 6 Hz), 55.52 – 55.47 (J_{CF} = 5 Hz), 48.82 – 48.77 (J_{CF} = 5 Hz), 36.85, 28.57, 15.77. LC-MS (ESI), m/z: $[M+1]^+$401.9, $C_{20}H_{21}F_2N_5O_2$.

*N-((2R,3R)-3-(2,4-difluorophenyl)-3-hydroxy-4-(1H-1,2,4-triazol-1-yl)butan-2-yl)-3-(4-methylpyridin-3-yl)propenamide (**A23**)*

White solid, yield 58%, ^{1}H NMR (400 MHz, DMSO-d_6) δ 8.34 (s, 1H, triazole ring), 8.19 (d, J = 5.9 Hz, 2H, pyridine ring), 7.87 (d, J = 8.9 Hz, 1H, –CONH–), 7.64 (s, 1H, triazole ring), 7.24 (td, J = 9.0, 6.8 Hz, 1H, pyridine ring), 7.19 – 7.10 (m, 2H, Ar-H), 6.88 (td, J = 8.4, 2.6 Hz, 1H, Ar-H), 5.73 (s, 1H, OH), 4.67 – 4.55 (m, 2H, $–CH_2–$), 4.17 (d, J = 14.4 Hz, 1H, –CH–), 2.90 (td, J = 7.4, 4.9 Hz, 2H, $–CH_2–$), 2.59 – 2.52 (m, 2H, $–CH_2–$), 2.33 (s, 3H, CH_3), 0.75 (d, J = 6.9 Hz, 3H, CH_3). ^{13}C NMR (101 MHz, DMSO-d_6) δ 171.84, 163.48 – 161.03 (J_{CF} = 247 Hz), 160.28 – 157.82 (J_{CF} = 248 Hz), 150.86, 149.92, 147.66, 145.49, 144.96, 135.56, 130.53 – 130.45 (J_{CF} = 8 Hz), 125.44, 124.98 – 124.85 (J_{CF} = 13 Hz), 111.34 – 111.14 (J_{CF} = 20 Hz), 104.59 – 104.31 (J_{CF} = 28 Hz), 77.76 – 77.70 (J_{CF} = 6 Hz), 55.51 – 55.47 (J_{CF} = 4 Hz), 48.88 – 48.82 (J_{CF} = 6 Hz), 35.69, 26.05, 18.69, 15.77. LC-MS (ESI), m/z: $[M+1]^+$415.9, $C_{21}H_{23}F_2N_5O_2$.

*N-((2R,3R)-3-(2,4-difluorophenyl)-3-hydroxy-4-(1H-1,2,4-triazol-1-yl)butan-2-yl)-3-(5-methylpyridin-3-yl)propenamide (**A24**)*

White solid, yield 55%, ^{1}H NMR (400 MHz, DMSO-d_6) δ 8.28 (d, J = 2.3 Hz, 1H, pyridine ring), 8.18 (s, 1H, triazole ring), 8.15 (d, J = 2.6 Hz, 1H, pyr

idine ring), 7.96 (d, J = 9.1 Hz, 1H, –CONH–), 7.63 (s, 1H, triazole ring), 7. 47 (t, J = 2.4 Hz, 1H, pyridine ring), 7.24 (td, J = 9.1, 6.9 Hz, 1H, Ar-H), 7. 14 (ddd, J = 11.9, 9.1, 2.6 Hz, 1H, Ar-H), 6.87 (td, J = 8.6, 2.8 Hz, 1H, Ar-H), 5.88 (s, 1H, OH), 4.65 – 4.53 (m, 2H, $–CH_2–$), 4.11 (d, J = 14.4 Hz, 1H, –CH–), 4.03 (q, J = 7.1 Hz, 3H, CH_3), 2.87 (td, J = 7.4, 4.6 Hz, 2H, $–CH_2–$), 2.57 (td, J = 7.4, 5.6 Hz, 2H, $–CH_2–$), 0.74 (d, J = 6.9 Hz, 3H, CH_3). ^{13}C NMR (101 MHz, DMSO-d_6) δ 171.70, 163.47 – 161.02(J_{CF} = 247 Hz), 160.27 – 157.81 (J_{CF} = 248 Hz), 150.82, 150.71, 148.00, 147.33, 144.89, 136.81, 136.43, 132.75, 124.98 – 124.85 (J_{CF} = 13 Hz), 111.34 – 111.13 (J_{CF} = 21 Hz), 104.55 – 104.27 (J_{CF} = 28 Hz), 77.63 – 77.58 (J_{CF} = 5 Hz), 55.46 – 55.41 (J_{CF} = 5 Hz), 48.79 – 48.74 (J_{CF} = 5 Hz), 36.89, 28.41, 21.19, 14.52. LC-MS (ESI), m/z: $[M+1]^+$415.9, $C_{21}H_{23}F_2N_5O_2$.

*N-((2R,3R)-3-(2,4-difluorophenyl)-3-hydroxy-4-(1H-1,2,4-triazol-1-yl)butan-2-yl)-3-(6-methylpyridin-3-yl)propenamide (**A25**)*

White solid, yield 53%, ^1H NMR (400 MHz, DMSO-d_6) δ 8.33 (d, J = 2.8 Hz, 1H, pyridine ring), 8.17 (s, 1H, triazole ring), 7.91 (d, J = 8.8 Hz, 1H, –CONH–), 7.63 (s, 1H, triazole ring), 7.55 (dd, J = 7.9, 2.3 Hz, 1H, pyridine ring), 7.23 (td, J = 9.0, 6.9 Hz, 1H, pyridine ring), 7.18 – 7.08 (m, 2H, Ar-H), 6.87 (td, J = 8.7, 2.9 Hz, 1H, Ar-H), 4.72 – 4.57 (m, 1H, OH), 4.53 (d, J = 15.3 Hz, 1H, –CH–), 4.03 (q, J = 7.1 Hz, 3H, CH_3), 2.86 (td, J = 7.3, 2.8 Hz, 2H, $–CH_2–$), 2.57 (dd, J = 14.8, 7.5 Hz, 2H, $–CH_2–$), 2.28 (s, 2H, $–CH_2–$), 0.74 (d, J = 6.8 Hz, 3H, CH_3). ^{13}C NMR (101 MHz, DMSO-d_6) δ 171.71, 163.46 – 161.02 (J_{CF} = 246 Hz), 160.26 – 157.81 (J_{CF} = 247 Hz), 155.79, 150.82, 149.33, 144.90, 136.72, 133.71, 130.53 – 130.44 (J_{CF} = 9 Hz),124.97 – 124.84 (J_{CF} = 15 Hz), 122.97, 111.30 – 111.10 (J_{CF} = 20 Hz), 104.30 – 104.02 (J_{CF} = 28 Hz), 77.62 – 77.57 (J_{CF} = 5 Hz), 55.40 – 55.36 (J_{CF} = 4 Hz), 48.77 – 48.71 (J_{CF} = 6 Hz), 37.02, 28.25, 23.83, 14.53. LC-MS (ESI), m/z: $[M+1]^+$415.9, $C_{21}H_{23}F_2N_5O_2$.

*N-((2R,3R)-3-(2,4-difluorophenyl)-3-hydroxy-4-(1H-1,2,4-triazol-1-yl)butan-2-yl)-3-(6-fluoropyridin-3-yl)propenamide (**A26**)*

White solid, yield 55%, ^1H NMR (400 MHz, DMSO-d_6) δ 8.18 (s, 1H, triazole ring), 8.11 (d, J = 2.9 Hz, 1H, pyridine ring), 7.88 (m, 2H, –CONH– and pyridine ring), 7.63 (s, 1H, triazole ring), 7.25 – 7.20 (m, 1H, Ar-H), 7.15 (ddd, J = 11.9, 9.1, 2.6 Hz, 1H, Ar-H), 7.07 (dd, J = 8.4, 2.9 Hz, 1H, pyridine ring), 6.88 (td, J = 8.6, 2.8 Hz, 1H, Ar-H), 5.76 (s, 1H, OH), 4.63 – 4.57 (m, 2H, $–CH_2–$), 4.15 (d, J = 14.5 Hz, 1H, –CH–), 2.91 (td, J = 7.4, 2.3 Hz, 2H, $–CH_2–$), 2.57 (td, J = 7.4, 2.9 Hz, 2H, $–CH_2–$), 0.74 (d, J = 6.9 Hz, 3H, CH_3). ^{13}C NMR (101 MHz, DMSO-d_6) δ 171.61, 163.34 – 161.02 (J_{CF} = 234 Hz), 163.34 – 160.91 (J_{CF} = 245 Hz), 160.28 – 157.69 (J_{CF} = 262 Hz), 150.86, 147.45 – 147.30 (J_{CF} = 15 Hz), 144.89, 142.56 – 142.48 (J_{CF} = 8 Hz), 135.26 – 135.22 (J_{CF} = 4 Hz), 130.45 – 130.36 (J_{CF} = 9 Hz), 124.91 – 124.78 (J_{CF} = 13 Hz), 111.38 – 111.17 (J_{CF} = 21 Hz), 109.58 – 109.21 (J_{CF} = 37 Hz), 104.33 – 104.05 (J_{CF} = 28 Hz), 77.71 – 77.66 (J_{CF} = 5 Hz), 60.22, 48.81 – 48.7

5 (J_{CF} = 6 Hz), 36.86, 27.56, 15.78. LC-MS (ESI), m/z: $[M+1]^+$419.9, $C_{20}H_{20}F_3N_5O_2$.

*N-((2R,3R)-3-(2,4-difluorophenyl)-3-hydroxy-4-(1H-1,2,4-triazol-1-yl)butan-2-yl)-3-(6-methoxy-pyridin-3-yl)propenamide (**A27**)*

White solid, yield 60%, ^{1}H NMR (400 MHz, DMSO-d_6) δ 8.15 (s, 1H, triazole ring), 8.02 (dd, J = 2.6, 0.7 Hz, 1H, pyridine ring), 7.84 (d, J = 8.6 Hz, 1H, –CONH–), 7.65 – 7.57 (m, 2H, triazole ring and pyridine ring), 7.28 – 7.09 (m, 2H, Ar-H), 6.92 – 6.83 (m, 1H, Ar-H), 6.72 (dd, J = 8.4, 0.7 Hz, 1H, pyridine ring), 5.73 (s, 1H, OH), 4.67 – 4.52 (m, 2H, $–CH_2–$), 4.05 (d, J = 14.5 Hz, 1H, –CH–), 3.69 (s, 3H, CH_3), 2.92 – 2.75 (m, 2H, $–CH_2–$), 2.52 – 2.49 (m, 2H, $–CH_2–$), 0.74 (d, J = 6.8 Hz, 3H, CH_3). ^{13}C NMR (101 MHz, DMSO-d_6) δ 171.80, 163.47 – 161.02 (J_{CF} = 247 Hz), 162.57, 160.14 – 157.69 (J_{CF} = 247 Hz), 150.84, 146.52, 144.86, 139.87, 130.52 – 130.42 (J_{CF} = 10 Hz), 129.74, 124.95 – 124.82 (J_{CF} = 13 Hz), 111.35 – 111.15 (J_{CF} = 20 Hz), 110.45, 104.59 – 104.31 (J_{CF} = 27 Hz), 77.69 – 77.64 (J_{CF} = 5 Hz), 55.47, 53.26, 48.77 – 48.71 (J_{CF} = 6 Hz), 37.27, 27.73, 15.75. LC-MS (ESI), m/z: $[M+1]^+$432.9, $C_{21}H_{23}F_2N_5O_3$.

Table S1. Crystal data and structure refinement for amine **4**.

Identification code	**4**	
Empirical formula	C12 H14 F2 N4 O	
Formula weight	268.27	
Temperature	173(2) K	
Wavelength	1.34139 Å	
Crystal system	Orthorhombic	
Space group	$P2_12_12_1$	
Unit cell dimensions	a = 8.9511(3) Å	α= 90°
	b = 10.4014(4) Å	β= 90°
	c = 13.8084(4) Å	δ= 90°
Volume	1285.62(8) Å^3	
Z	4	
Density (calculated)	1.386 Mg/m^3	
Absorption coefficient	0.610 mm^{-1}	
F(000)	560	
Crystal size	0.400 x 0.200 x 0.200 mm^3	
Theta range for data collection	4.630 to 59.991°	
Index ranges	-11<=h<=11, -13<=k<=13, -17<=l<=17	
Reflections collected	14128	
Independent reflections	2901 [R(int) = 0.0284]	
Completeness to theta = 53.594°	99.8 %	
Absorption correction	Semi-empirical from equivalents	
Max. and min. transmission	0.752 and 0.656	
Refinement method	Full-matrix least-squares on F^2	
Data / restraints / parameters	2901 / 0 / 186	
Goodness-of-fit on F^2	1.044	
Final R indices [I>2sigma(I)]	R1 = 0.0266, wR2 = 0.0663	
R indices (all data)	R1 = 0.0291, wR2 = 0.0679	
Absolute structure parameter	-0.09(5)	
Extinction coefficient	0.0043(7)	
Largest diff. peak and hole	0.163 and -0.125 e.Å^{-3}	

Table S2. *In silico* ADME/T prediction of the tested compounds.

ADME/T parameters	**A1**	**A3**	**A6**	**A9**	**A15**	**A21**	**A25**	**FCZ**[g]
Aqueous solubility[a]	3	2	2	2	2	3	3	4
BBB penetration[b]	2	2	2	2	2	3	3	3
A log P98[c]	2.861	3.066	3.347	4.261	3.731	2.203	1.993	0.75
PSA[d]	78.796	78.796	78.796	78.796	78.796	90.057	90.057	76.556
PPB[e]	13.2859	13.3719	13.9601	13.0498	14.5907	14.7315	14.7501	10.2619
Ames mutagenicity	Non-mutagen	Non-mutagen	Non-mutagen	Non-mutagen	Non-mutagen	Non-mutagen	Non-mutagen	Non-mutagen
Rat_Female_FDA[f] carcinogenicity	Non-carcinogen	Non-carcinogen	Non-carcinogen	Non-carcinogen	Non-carcinogen	Non-carcinogen	Non-carcinogen	Non-carcinogen
Mouse_FDA carcinogenicity	Non-carcinogen	Non-carcinogen	Non-carcinogen	Non-carcinogen	Non-carcinogen	Non-carcinogen	Non-carcinogen	Non-carcinogen
Skin_Irritancy	None	None	None	None	None	None	None	None
Skin_Sensitization	Strong	Strong	Strong	Strong	Weak	Strong	Strong	Strong
Ocular_Irritancy	Moderate	Mild	Moderate	Moderate	Moderate	Moderate	Severe	Moderate
DTP[h]	Toxic	Toxic	Non-Toxic	Toxic	Toxic	Non-Toxic	Non-Toxic	Toxic

a. Level of aqueous solubility predicted: 0 (extremely low), 1 (very low, but possible), 2 (low), 3 (good), 4 (optimal), 5 (too soluble), 6 (warning: molecules with one or more unknown A log P calculations).

b. BBB (blood brain barrier), level blood brain barrier penetration prediction: 0 (very high penetration), 1 (high), 2 (medium), 3 (low), 4 (undefined).

c. A log P98 (atom-based log P) ($\leqslant$-2.0 or $\geqslant$7.0: very low absorption).

d. PSA (polar surface area) (>150: very low absorption).

e. PPB, plasma protein binding.

f. FDA, food and drug administration.

g. FCZ, fluconazole.

h. DTP, development toxicity potential.

^{1}H NMR, ^{13}C NMR, HPLC purity and ESI-MS of All Compounds

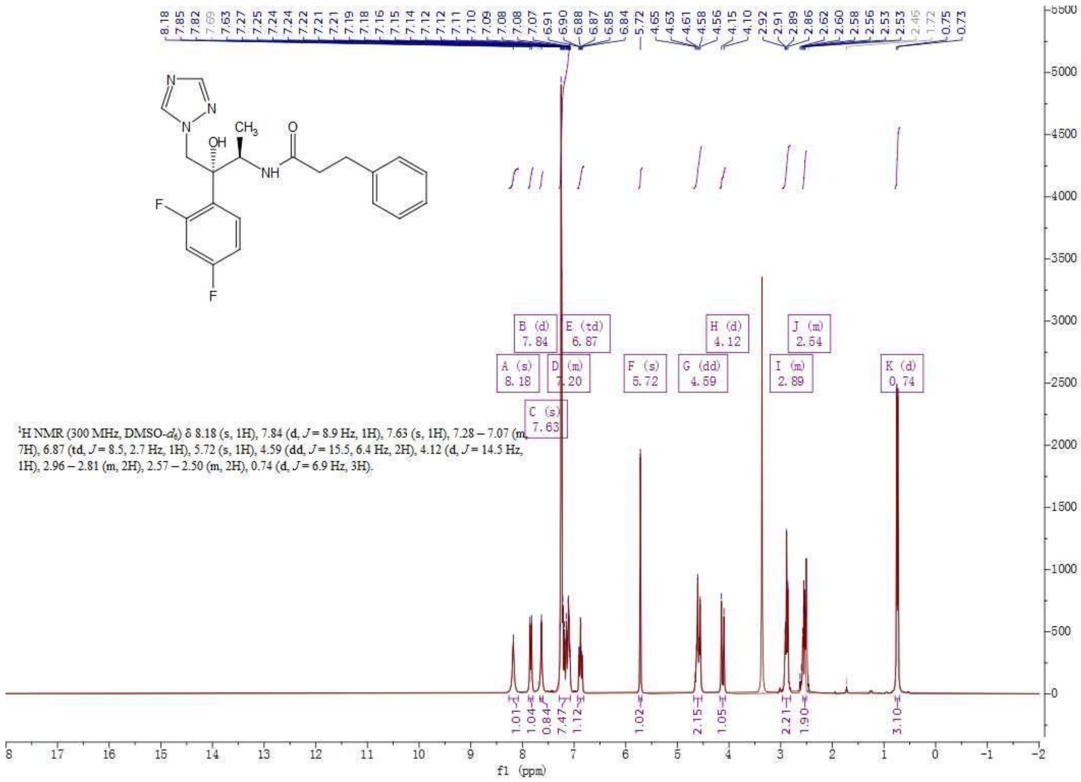

Figure S1. ^{1}H-NMR of A1

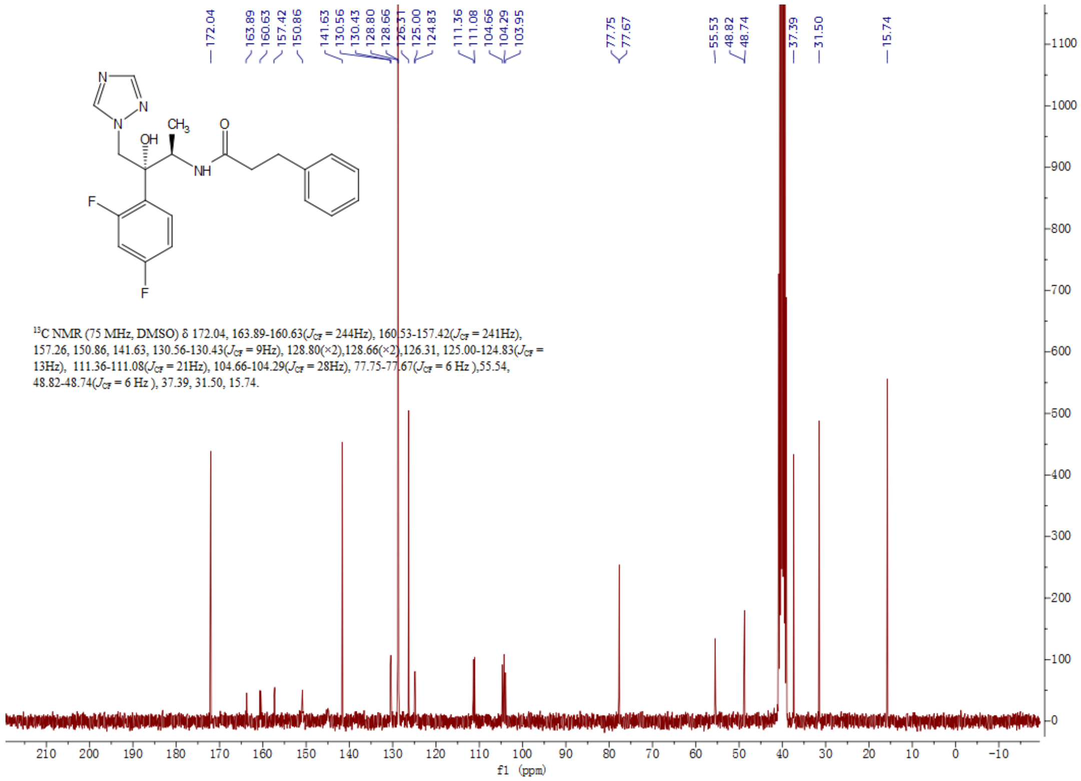

Figure S2. ^{13}C-NMR of A1

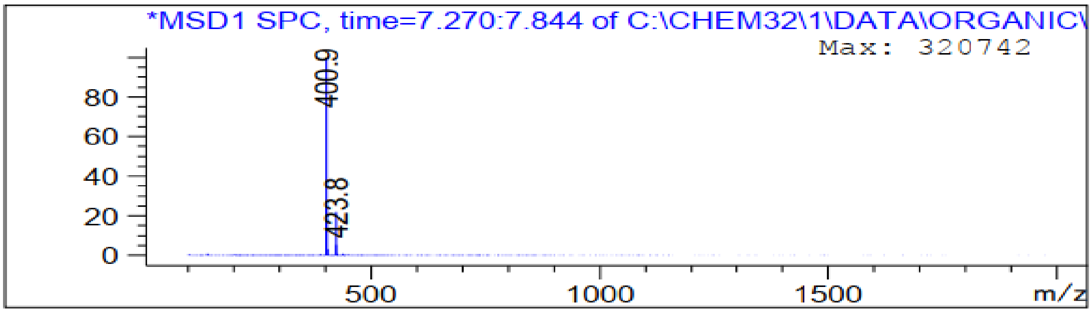

Figure S3. ESI-MS of A1

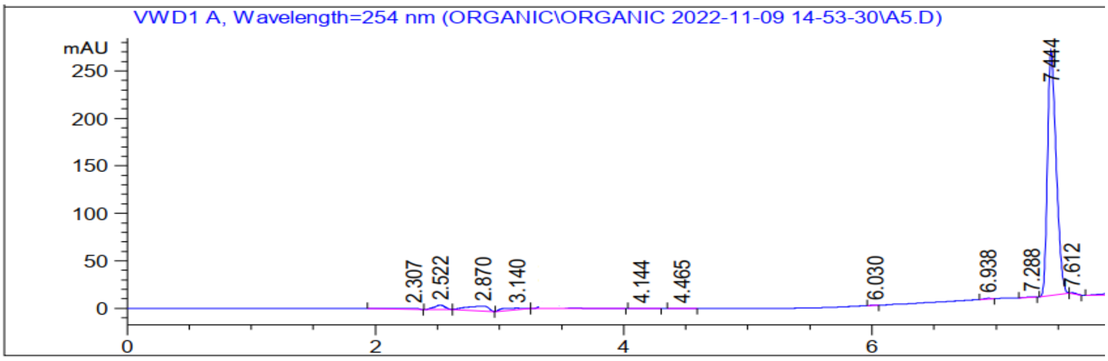

Figure S4. HPLC purity of A1

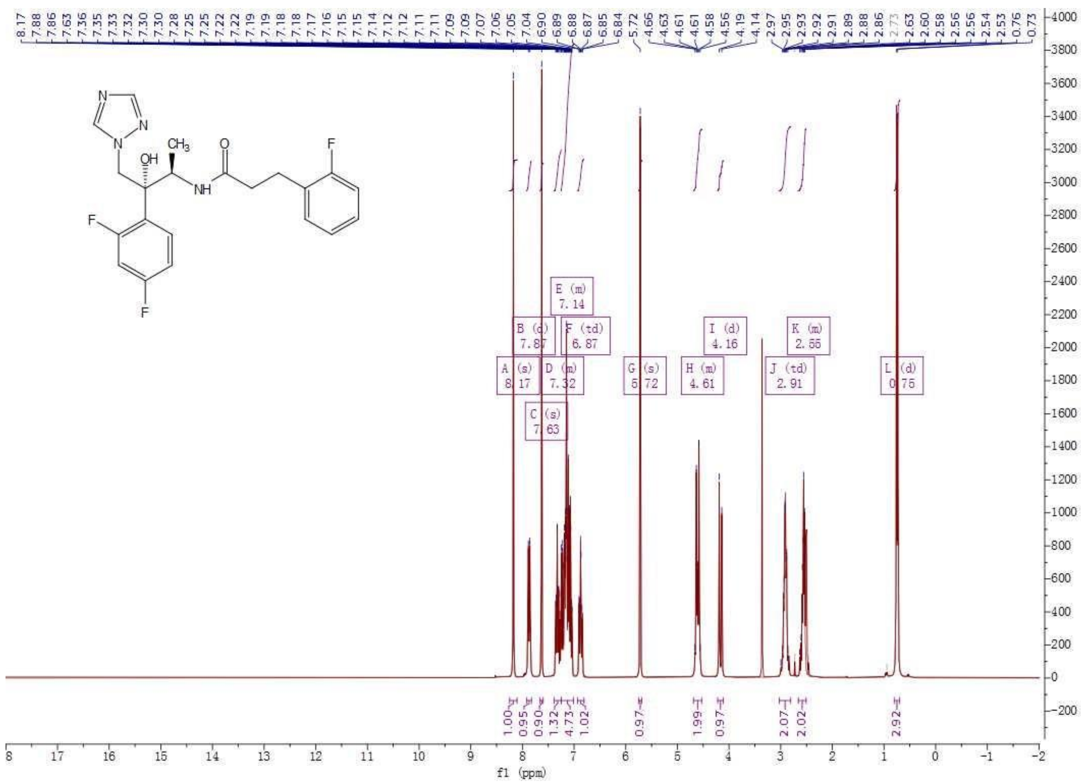

Figure S5. ^{1}H-NMR of A2

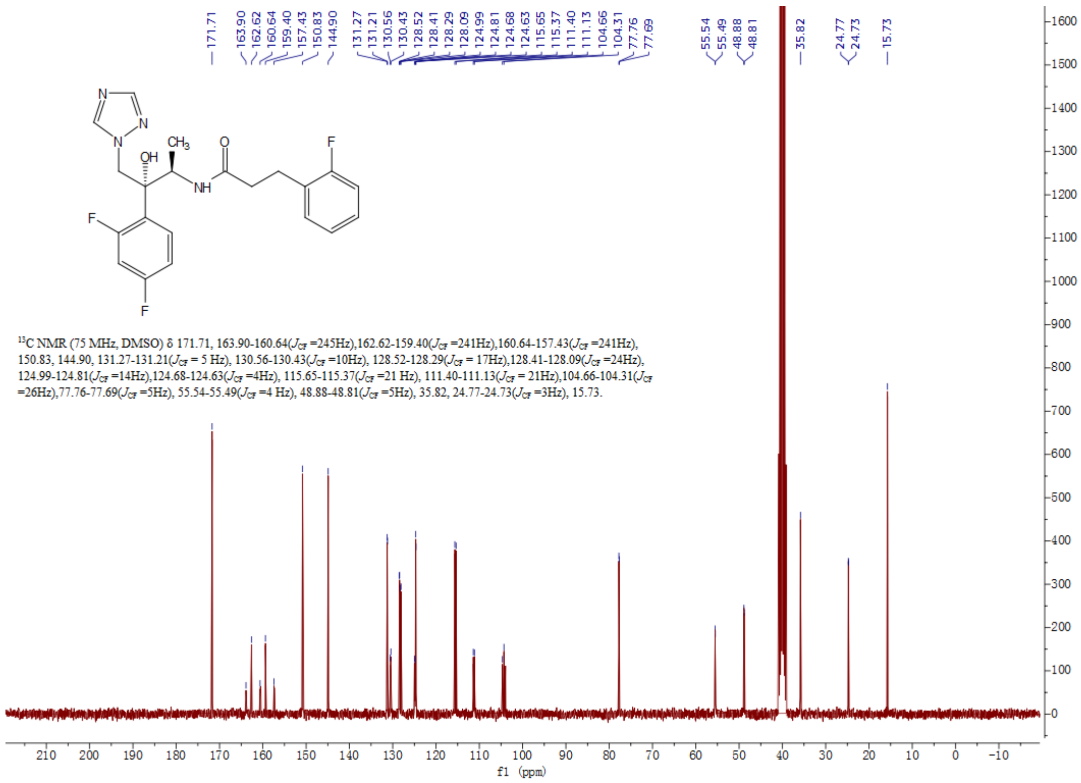

Figure S6. ^{13}C-NMR of A2

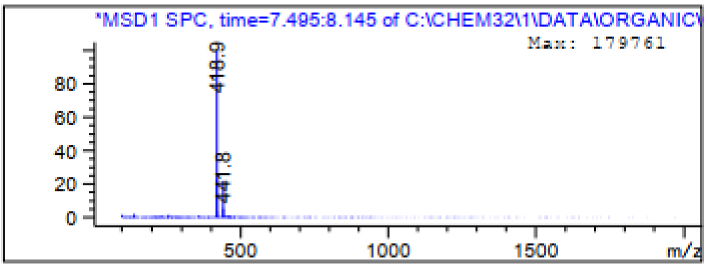

Figure S7. ESI-MS of A2

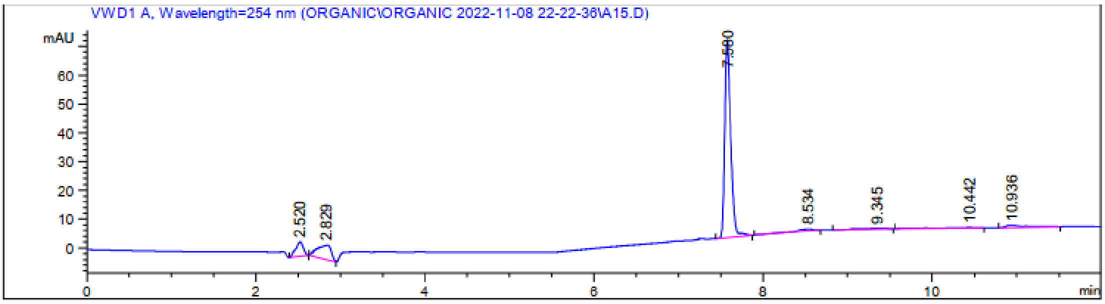

Figure S8. HPLC purity of A2

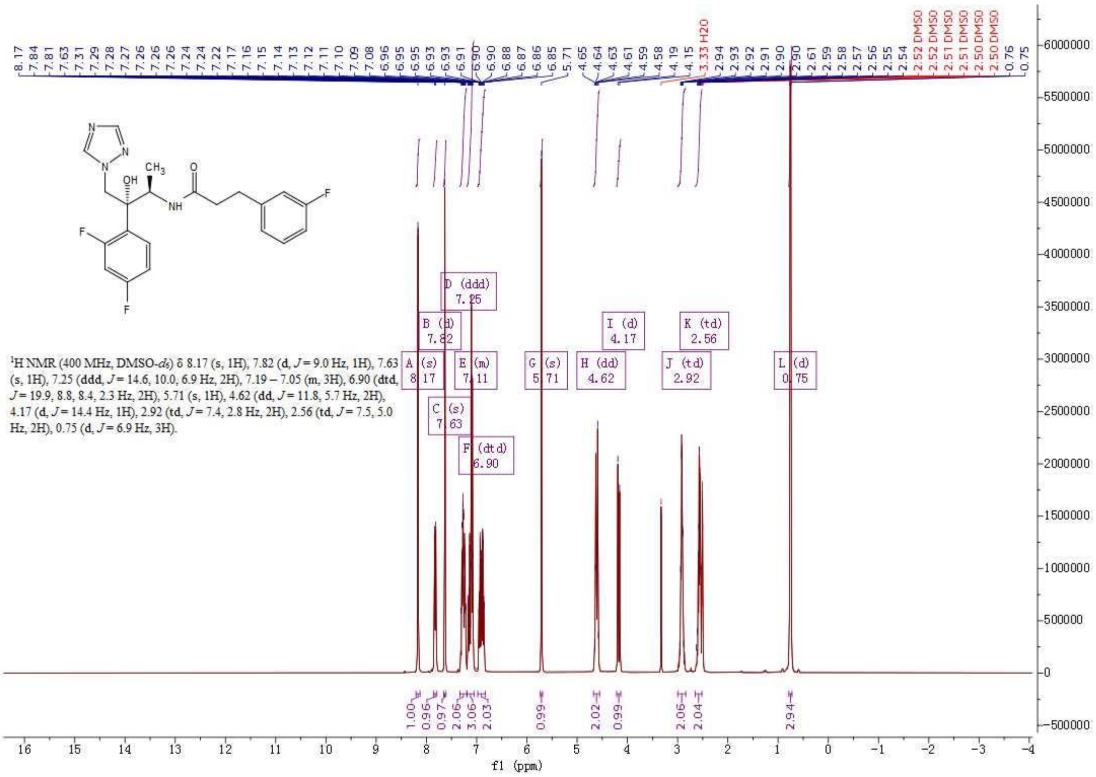

Figure S9. ^{1}H-NMR of A3

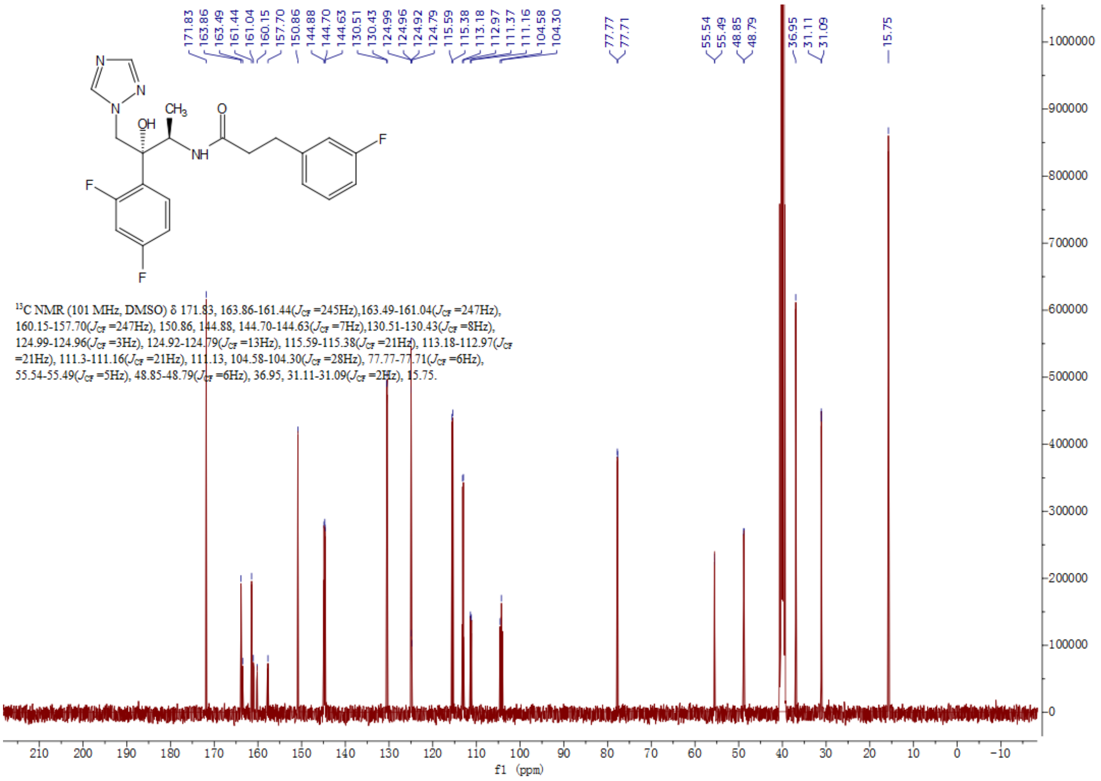

Figure S10. ^{13}C-NMR of A3

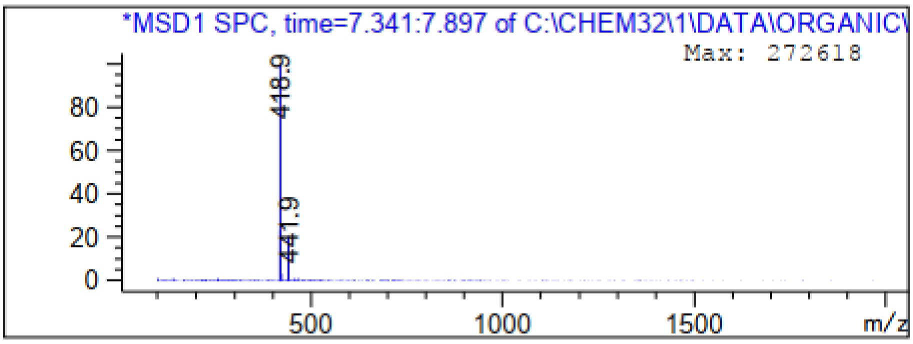

Figure S11. ESI-MS of A3

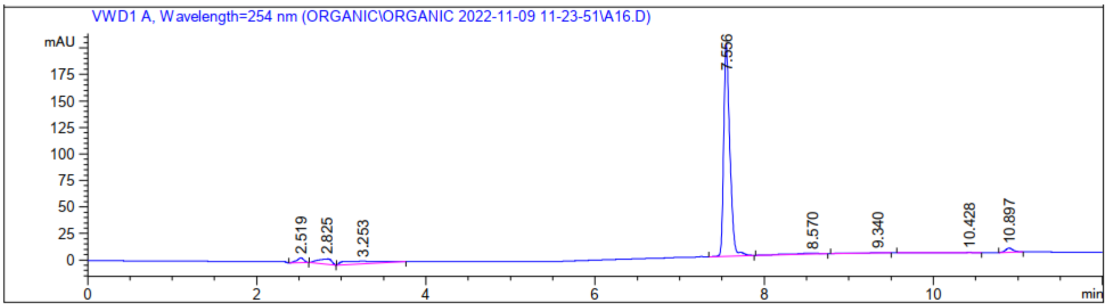

Figure S12. HPLC purity of A3

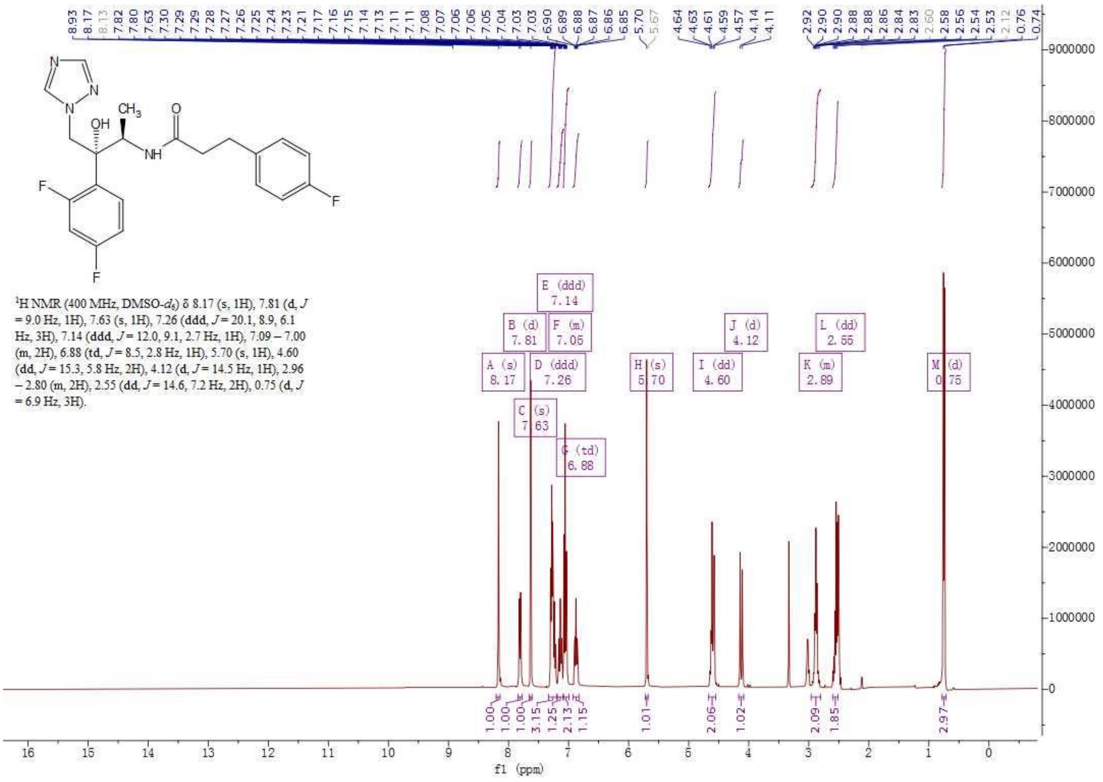

Figure S13. ¹H-NMR of A4

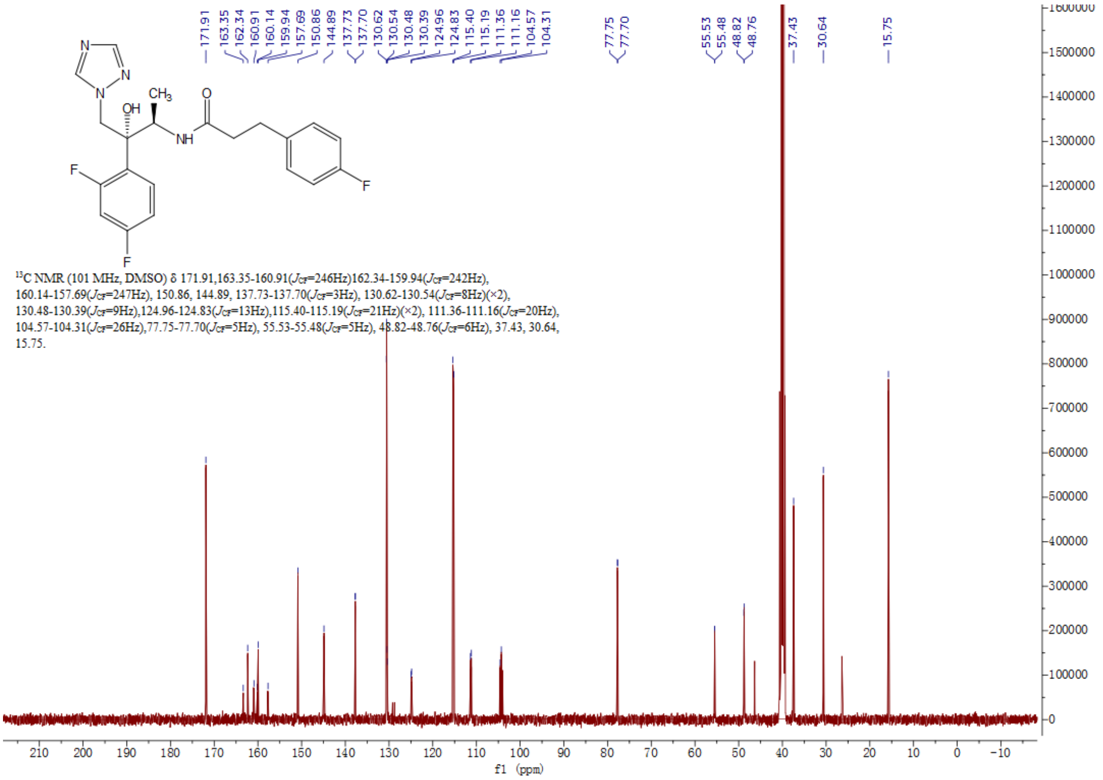

Figure S14. ¹³C-NMR of A4

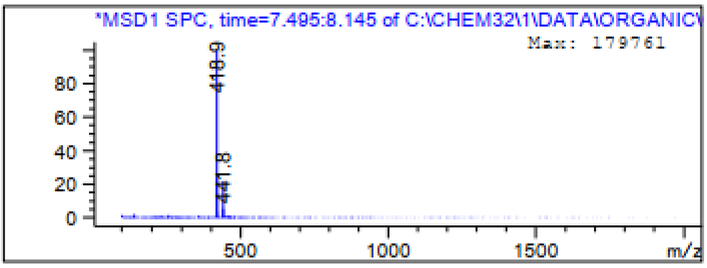

Figure S15. ESI-MS of A4

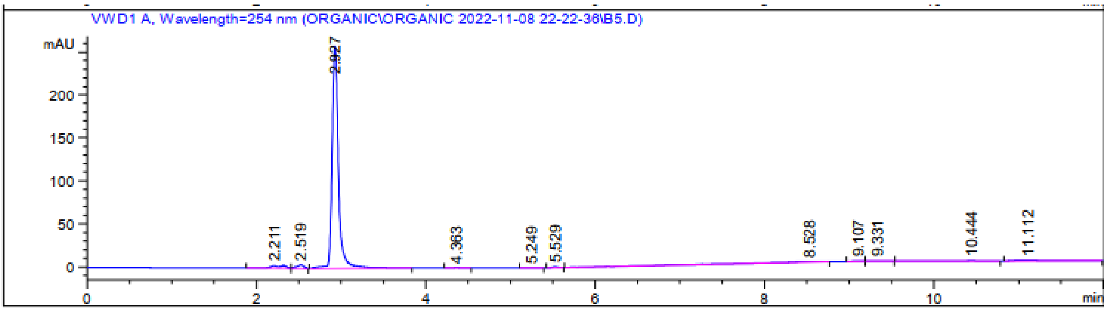

Figure S16. HPLC purity of A4

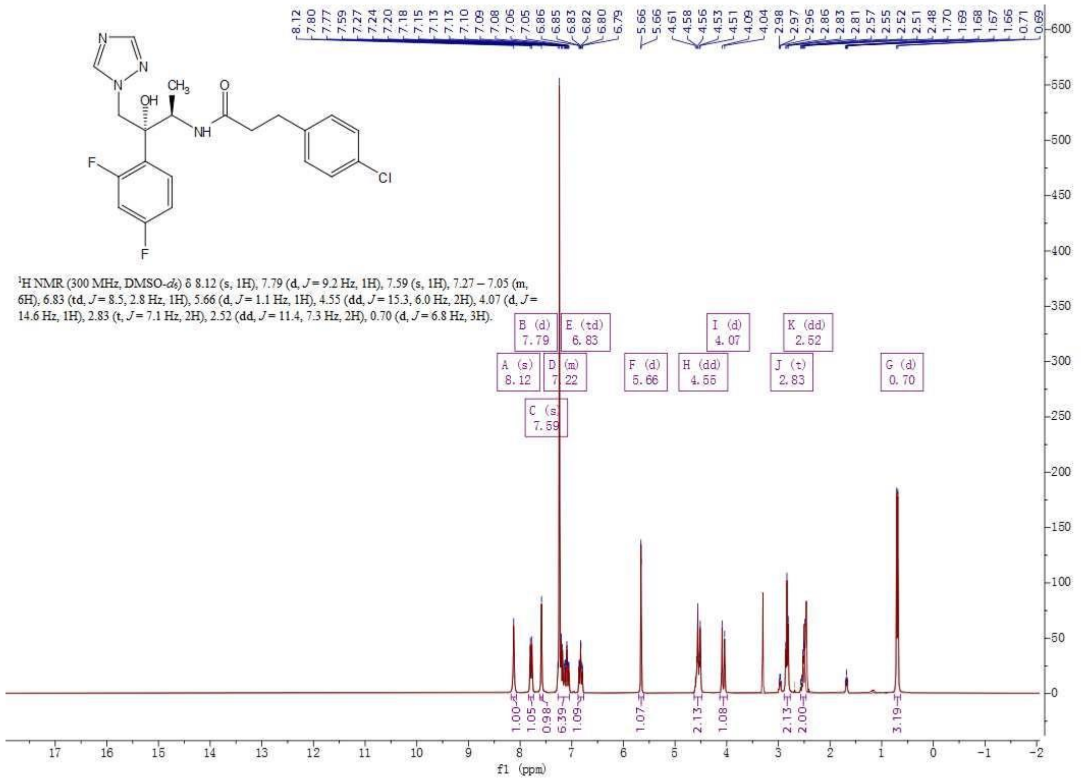

Figure S17. ^{1}H-NMR of A5

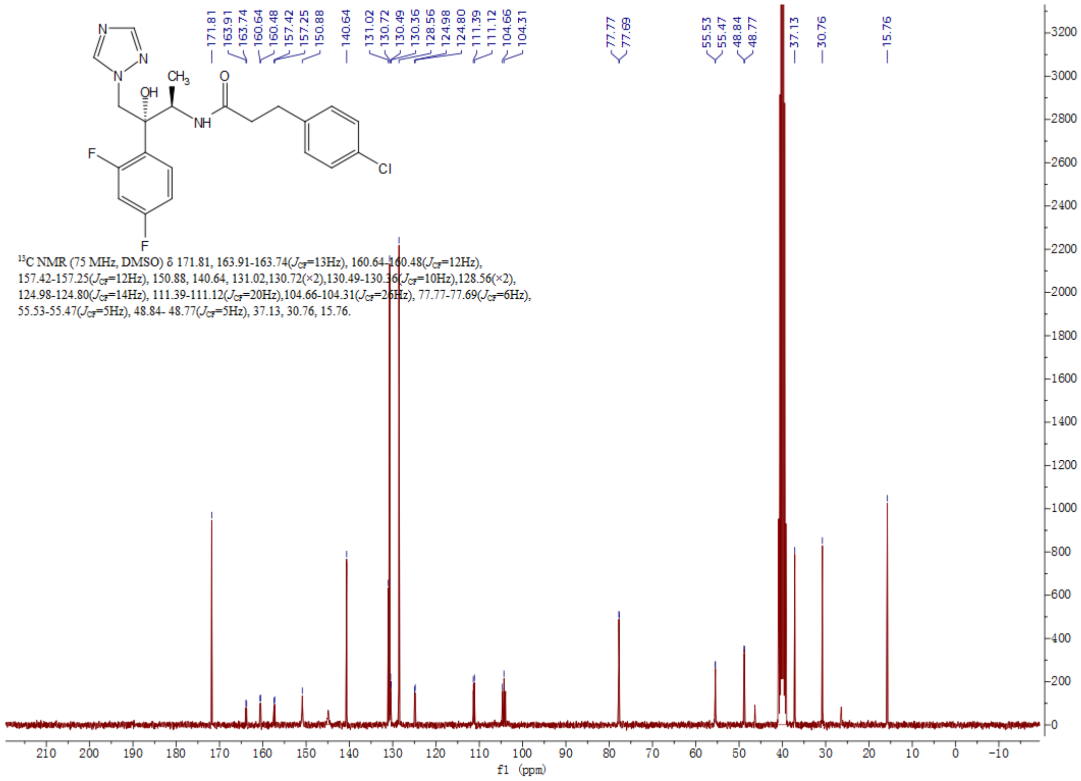

Figure S18. ^{13}C-NMR of A5

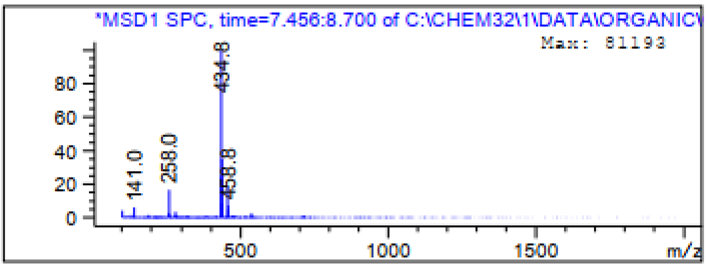

Figure S19. ESI-MS of A5

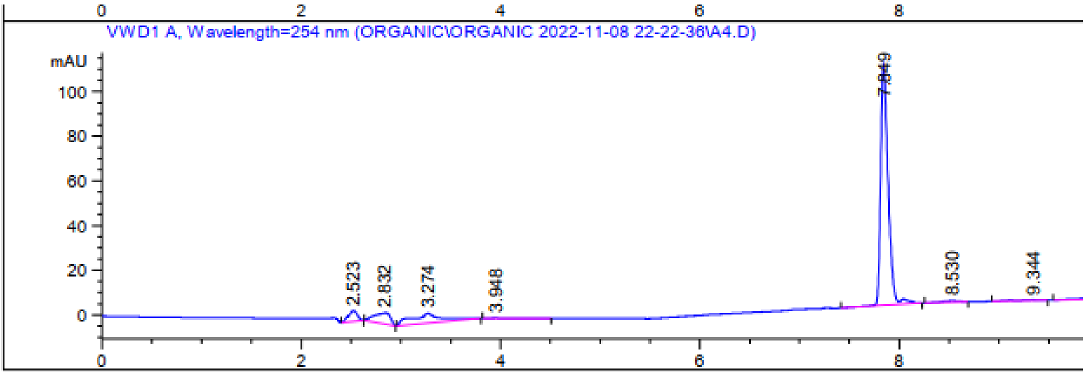

Figure S20. HPLC purity of A5

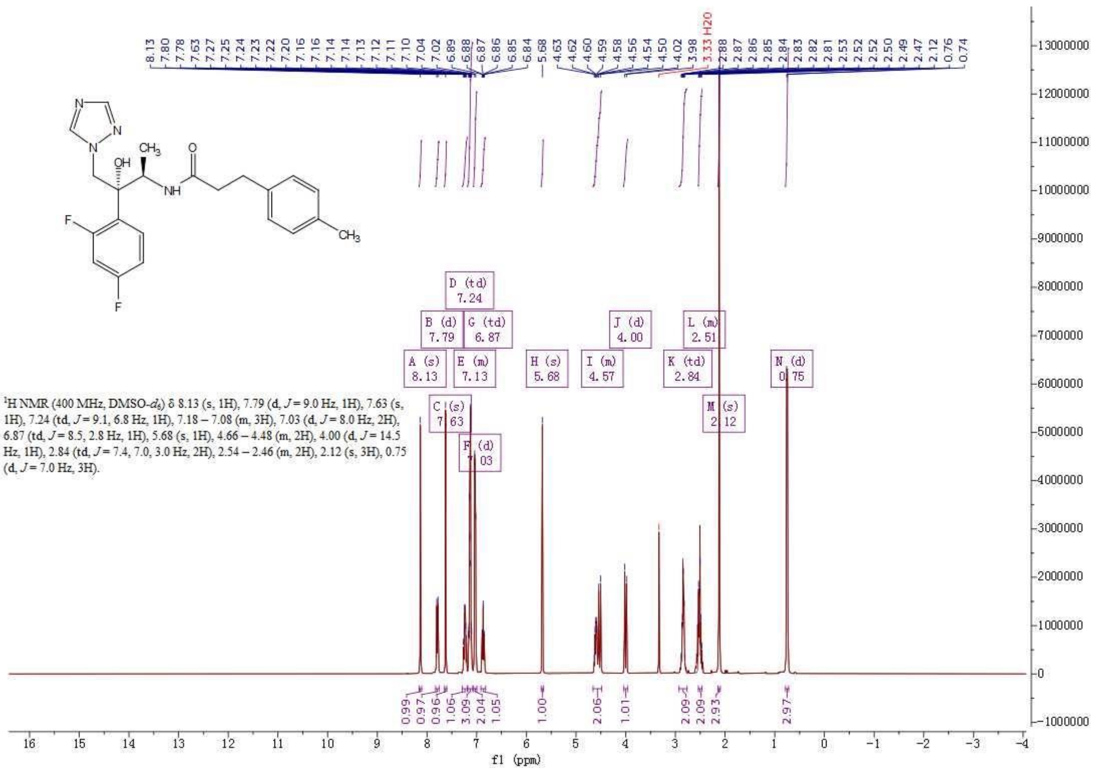

Figure S21. ^{1}H-NMR of A6

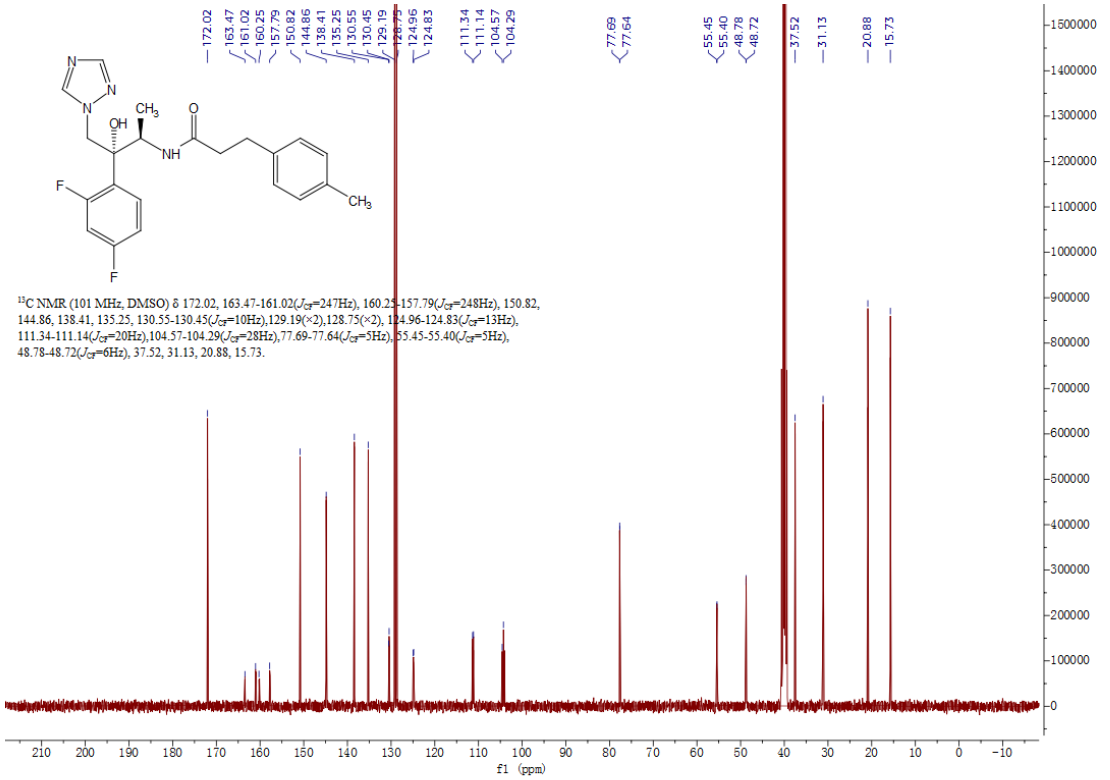

Figure S22. ^{13}C-NMR of A6

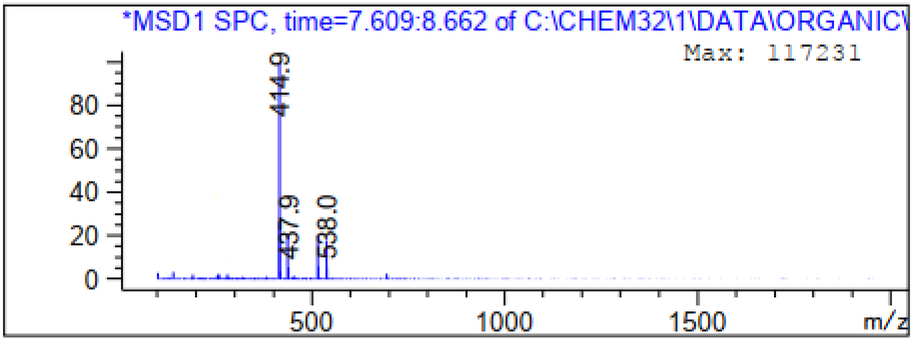

Figure S23. ESI-MS of A6

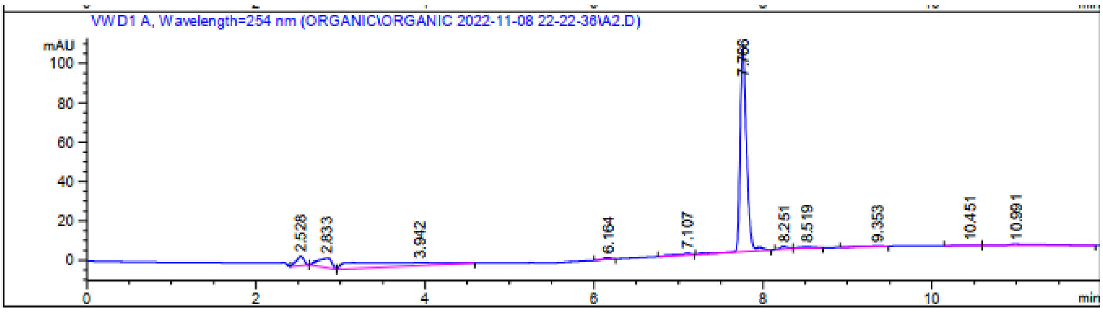

Figure S24. HPLC purity of A6

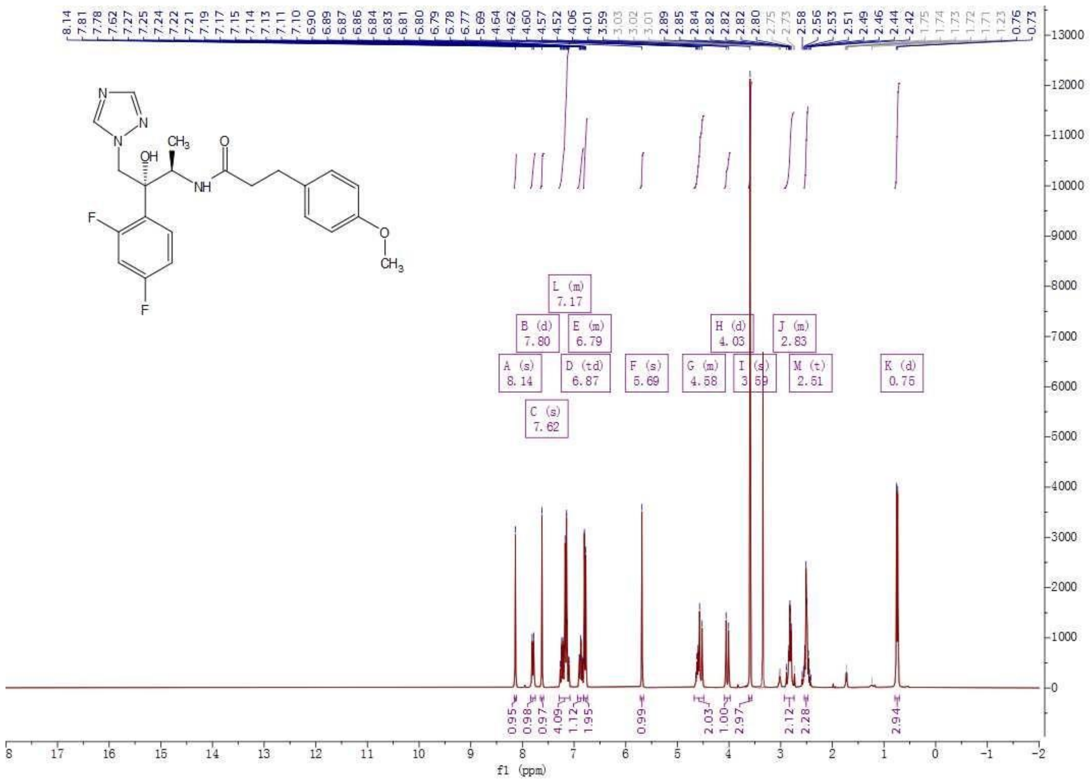

Figure S25. ^{1}H-NMR of A7

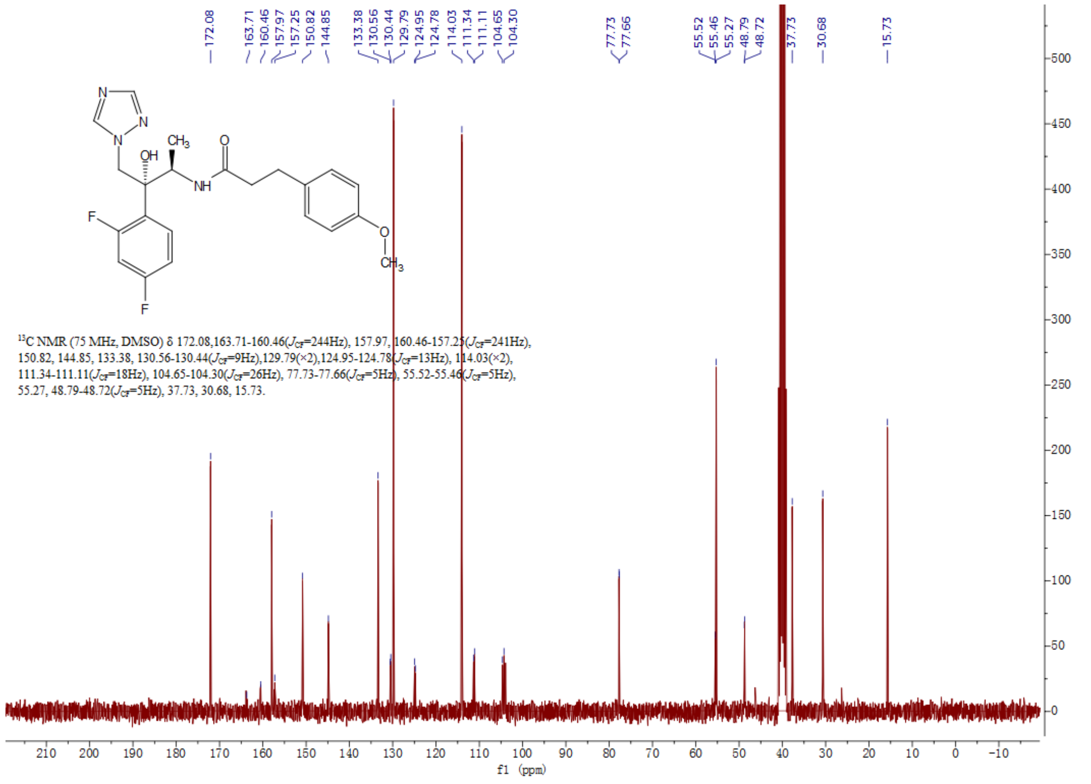

Figure S26. ^{13}C-NMR of A7

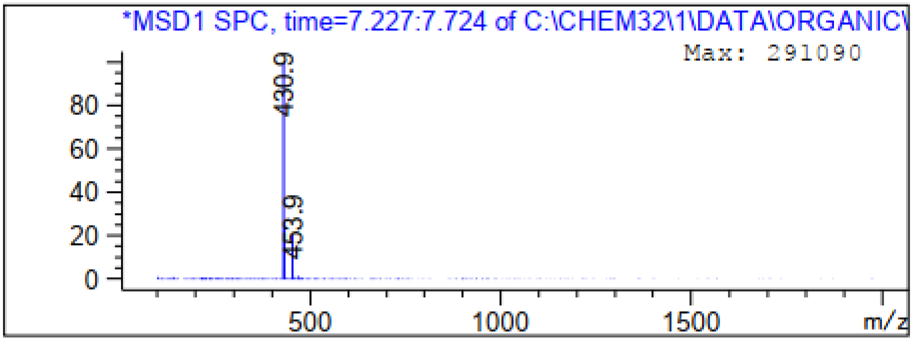

Figure S27. ESI-MS of A7

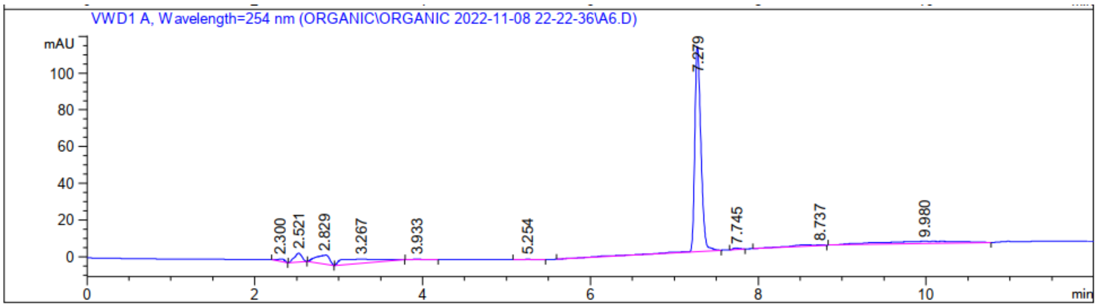

Figure S28. HPLC purity of A7

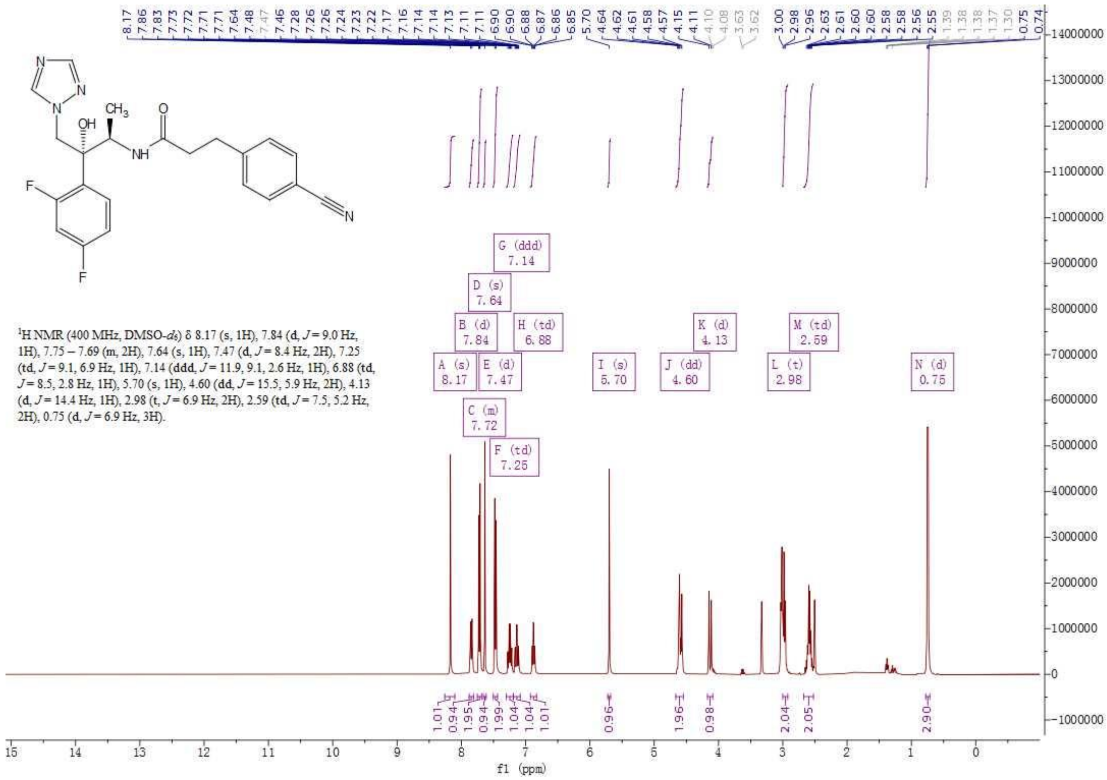

Figure S29. ^{1}H-NMR of A8

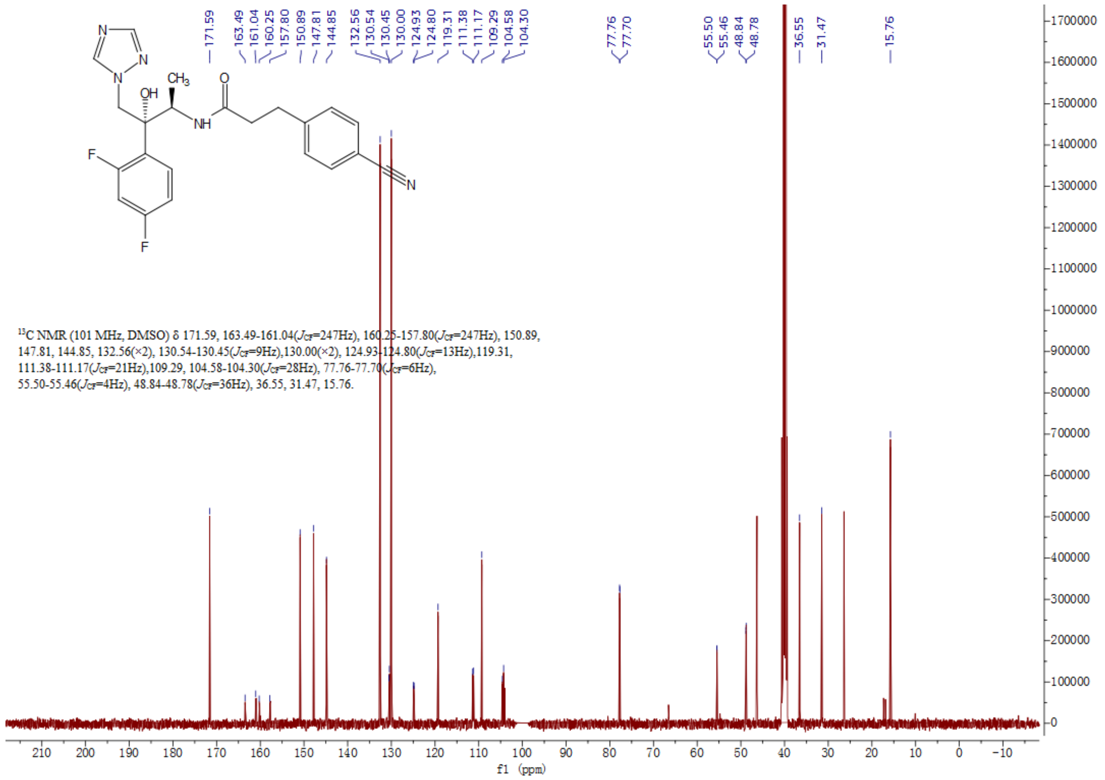

Figure S30. ^{13}C-NMR of A8

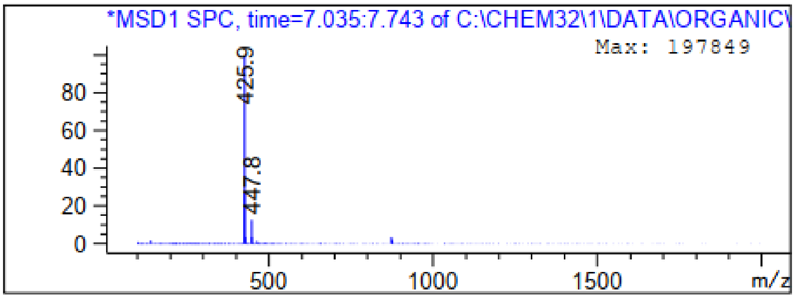

Figure S31. ESI-MS of A8

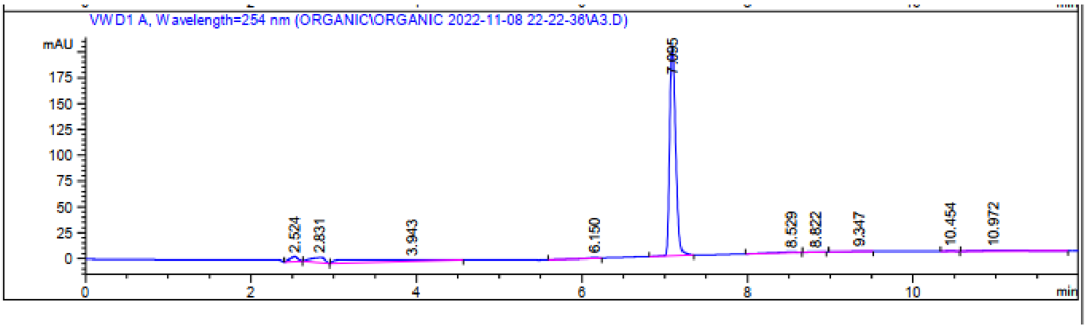

Figure S32. HPLC purity of A8

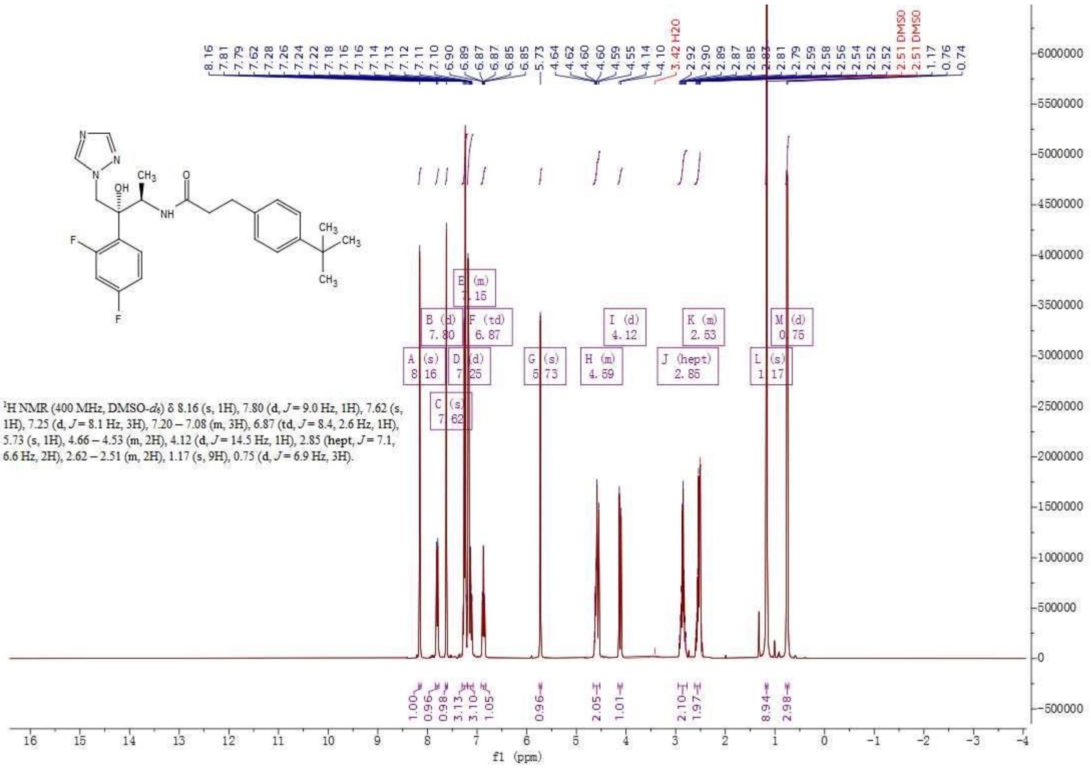

Figure S33. 1H-NMR of A9

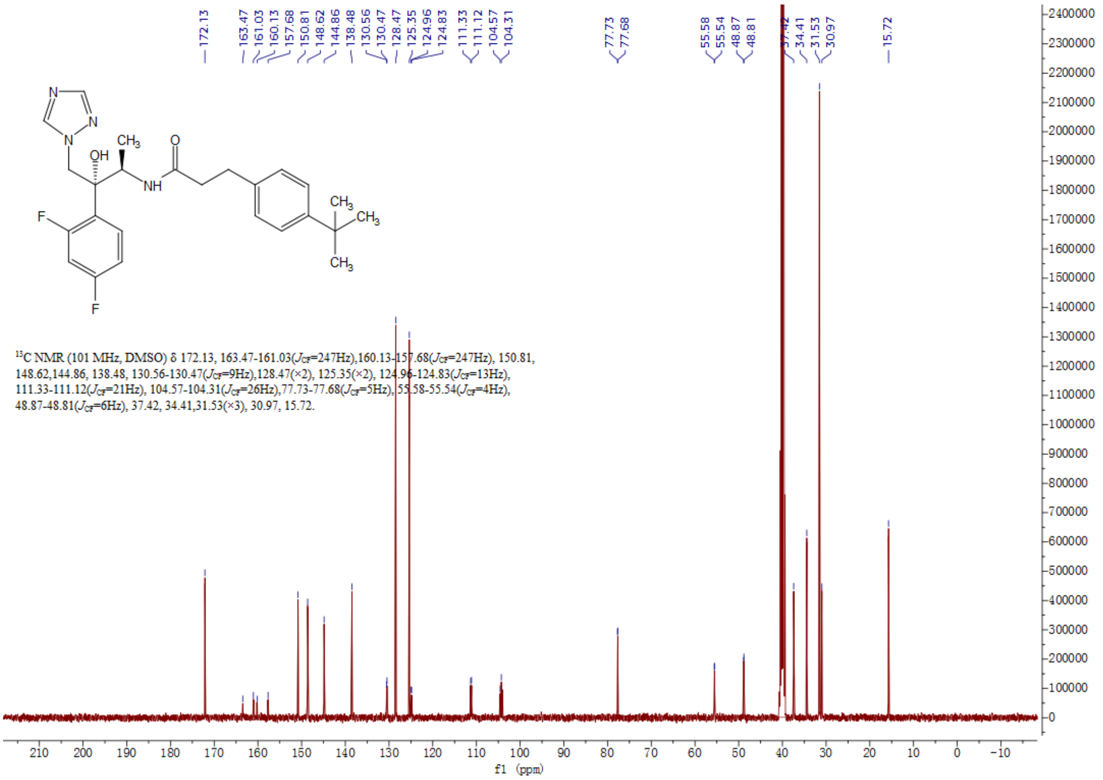

Figure 34S. 13C-NMR of A9

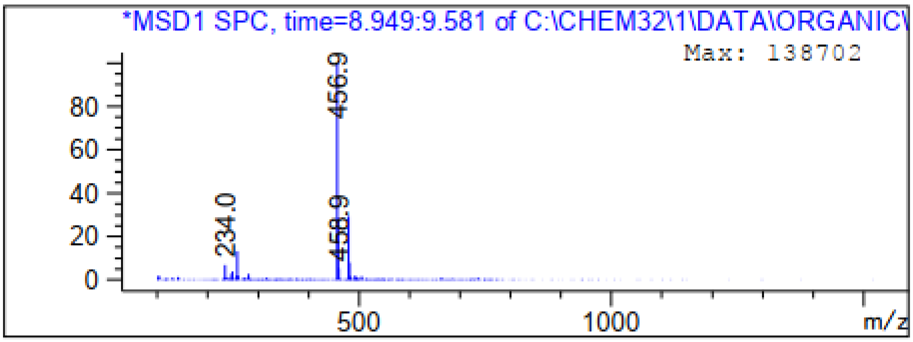

Figure S35. ESI-MS of A9

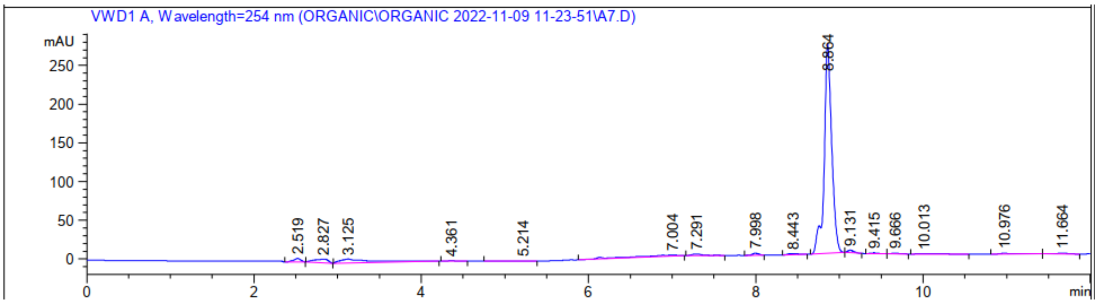

Figure S336. HPLC purity of A9

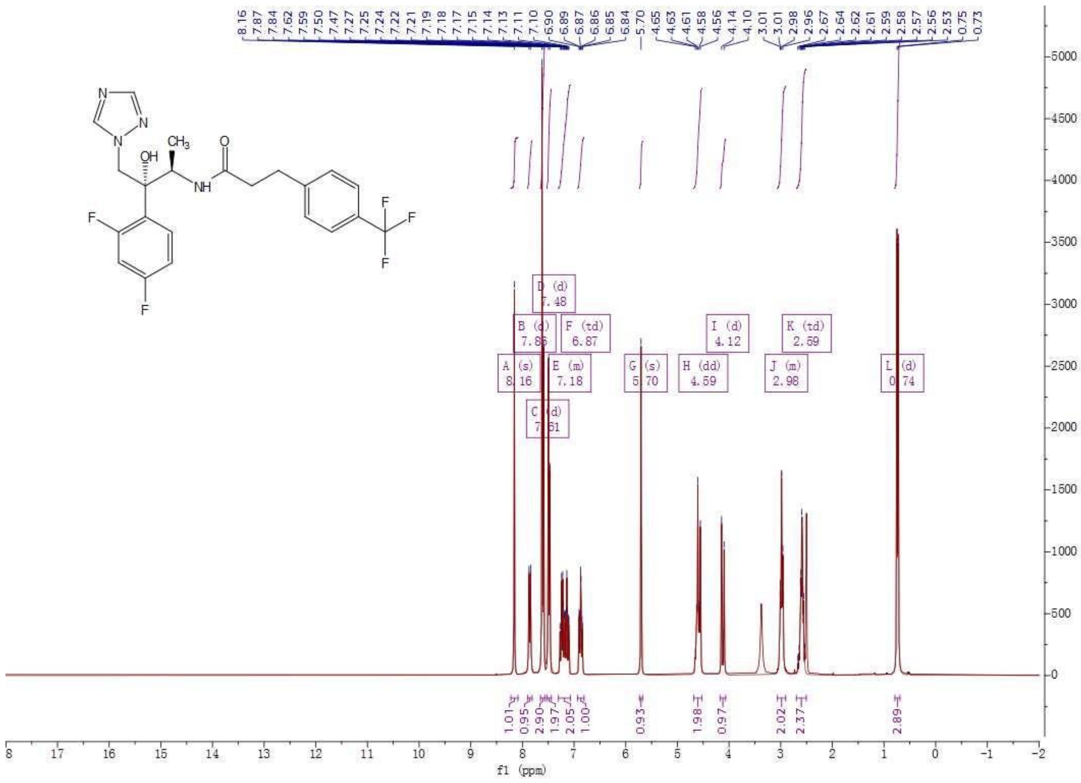

Figure S37. ^{1}H-NMR of A10

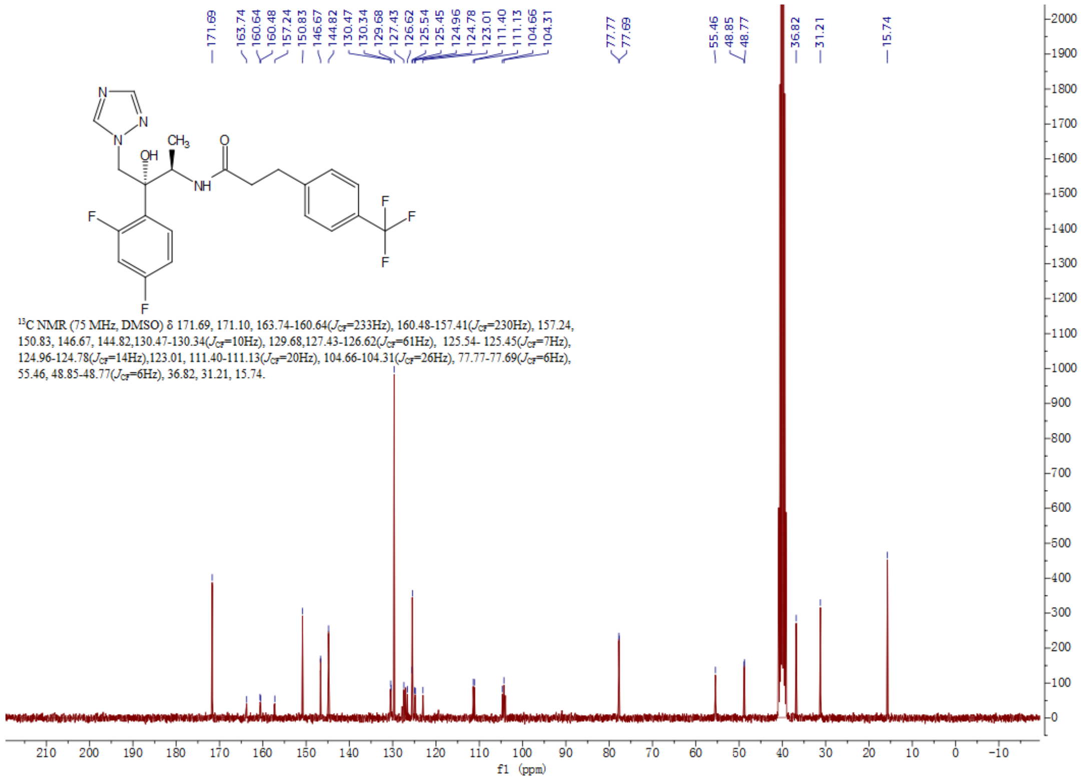

Figure S38. ^{13}C-NMR of A10

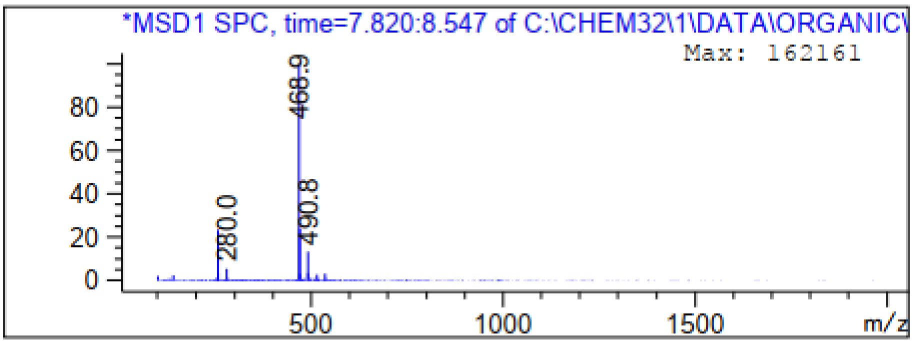

Figure S39. ESI-MS of A10

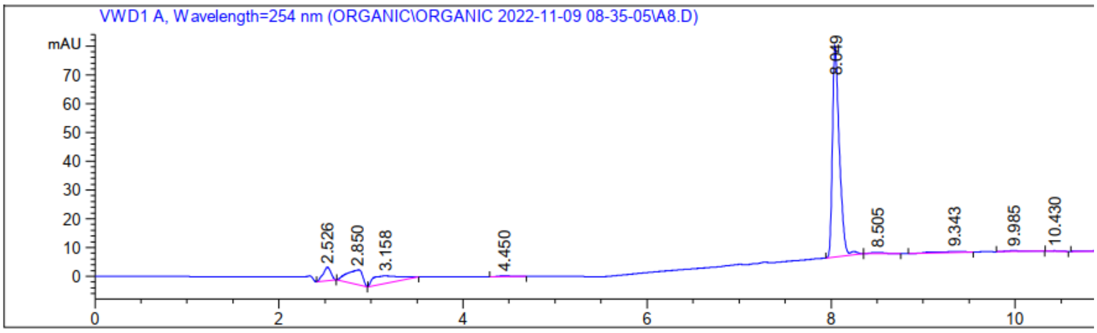

Figure S40. HPLC purity of A10

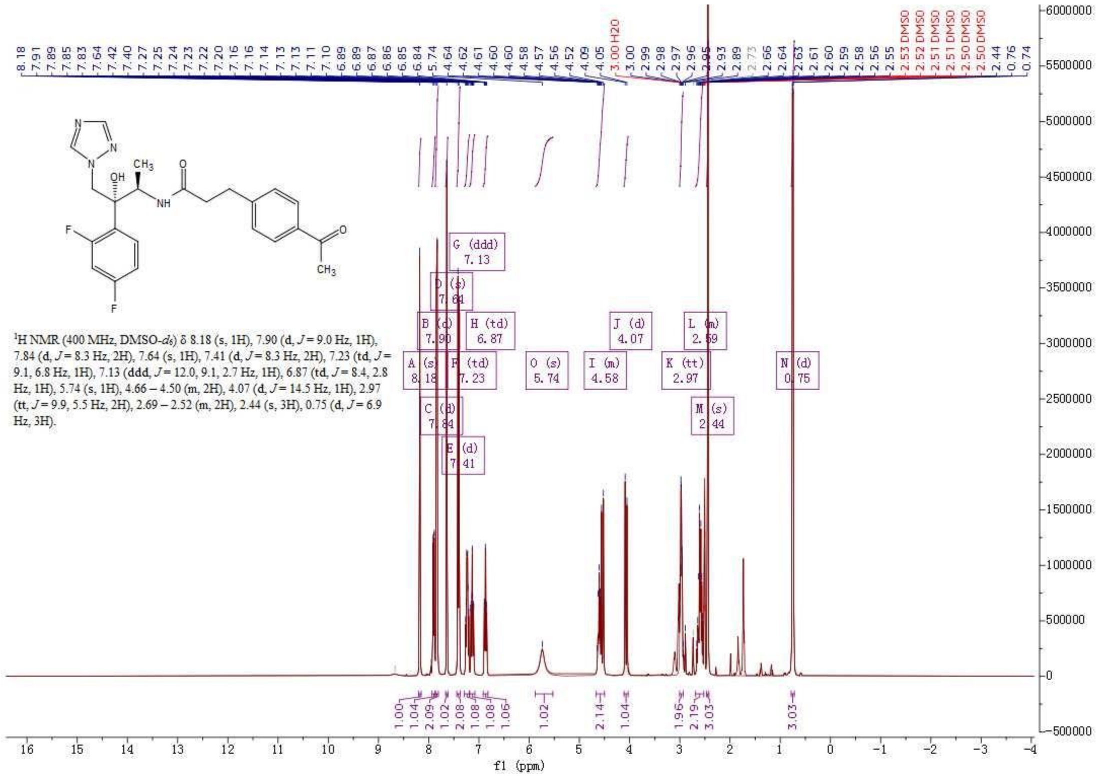

Figure S41. ^{1}H-NMR of A11

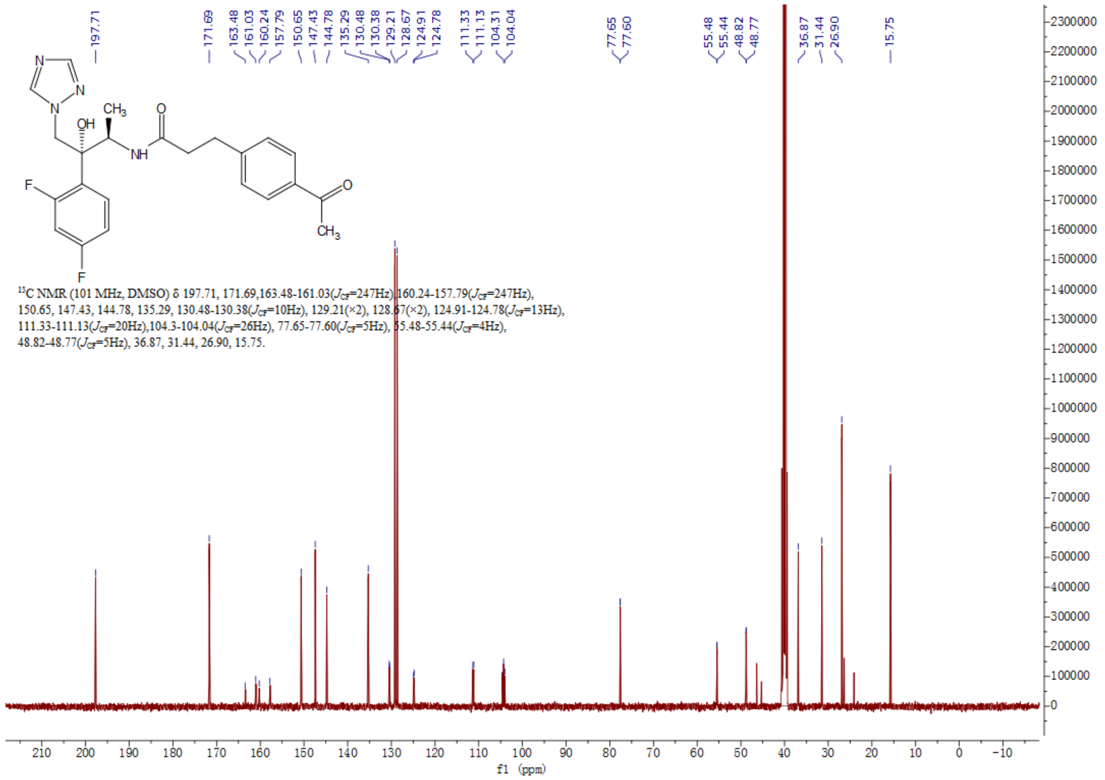

Figure S42. ^{13}C-NMR of A11

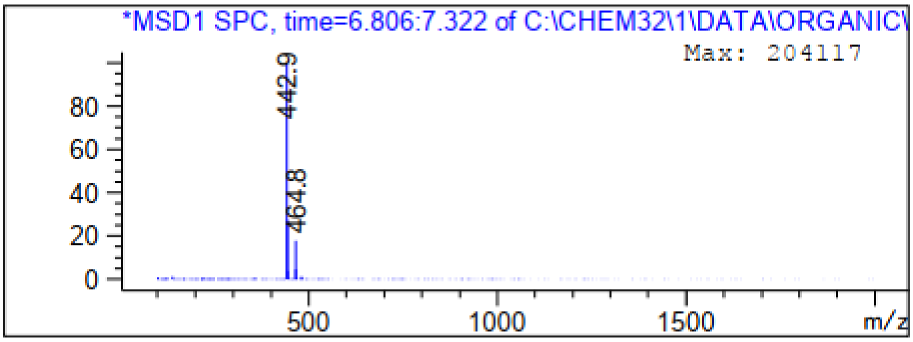

Figure S43. ESI-MS of A11

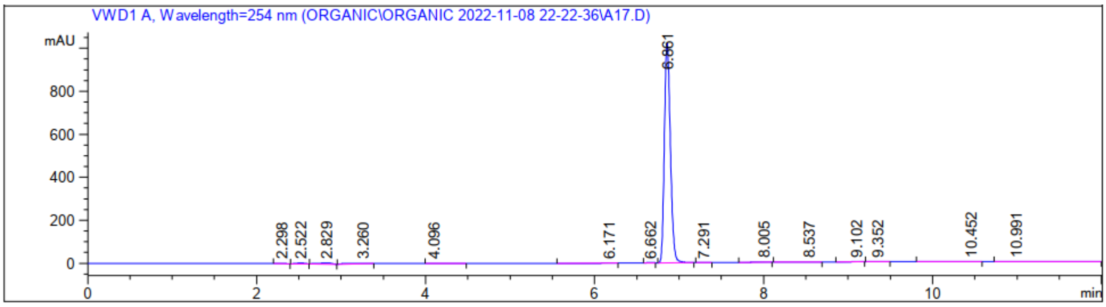

Figure S44. HPLC purity of A11

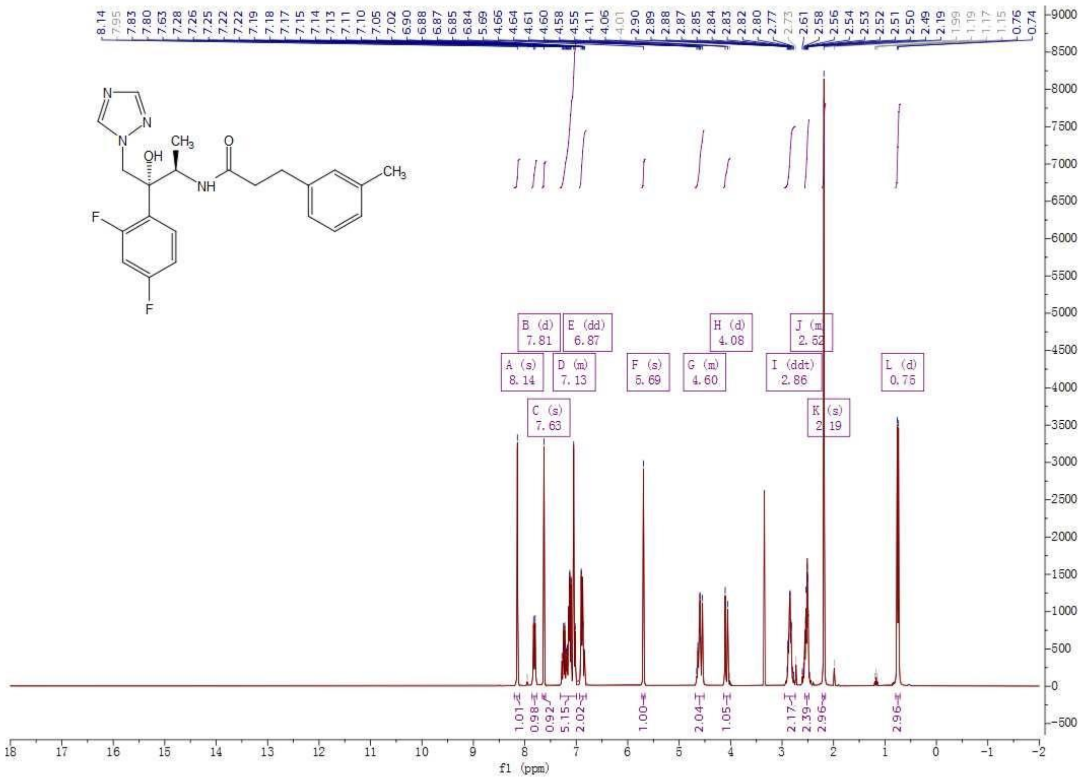

Figure S45. ^{1}H-NMR of A12

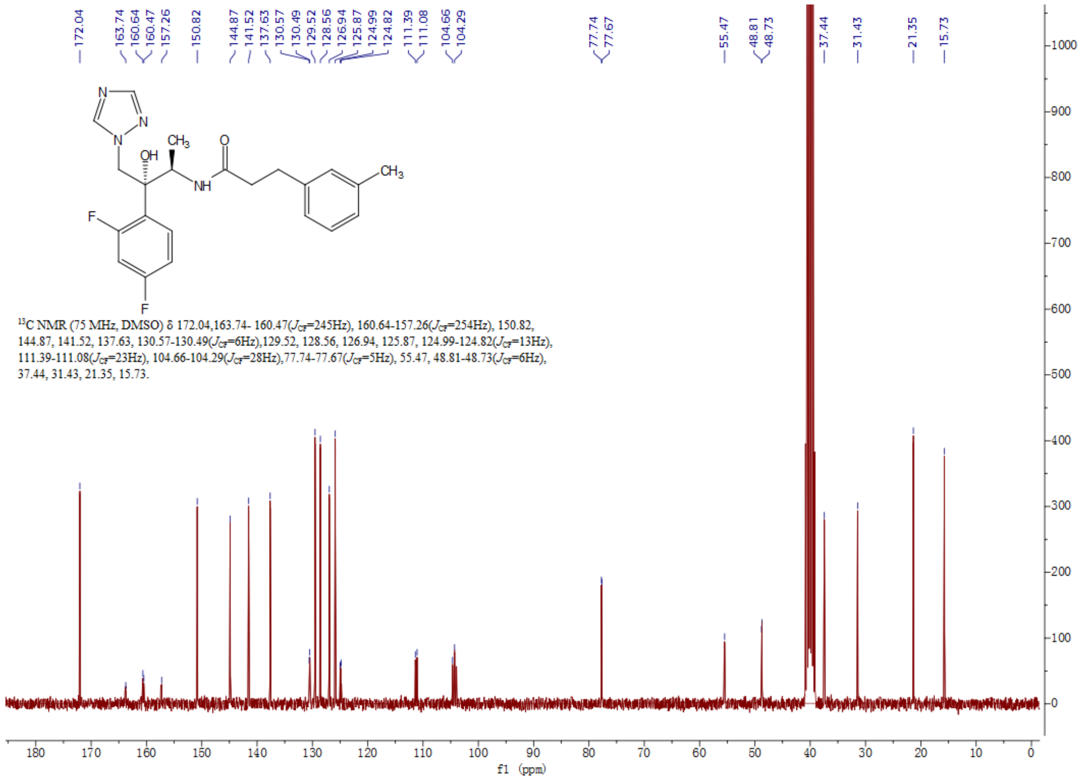

Figure S46. ^{13}C-NMR of A12

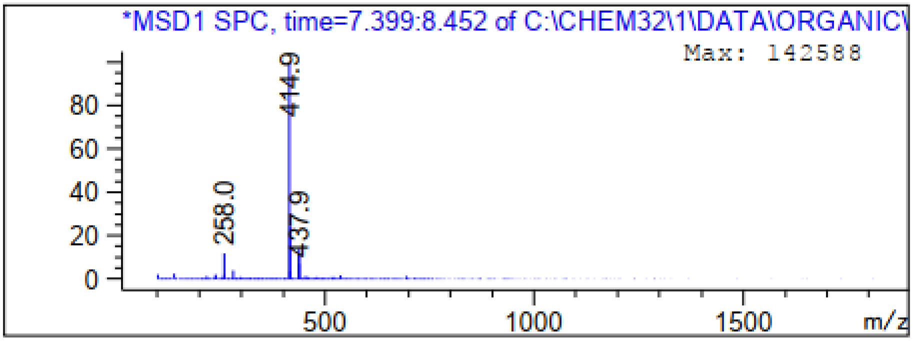

Figure S47. ESI-MS of A12

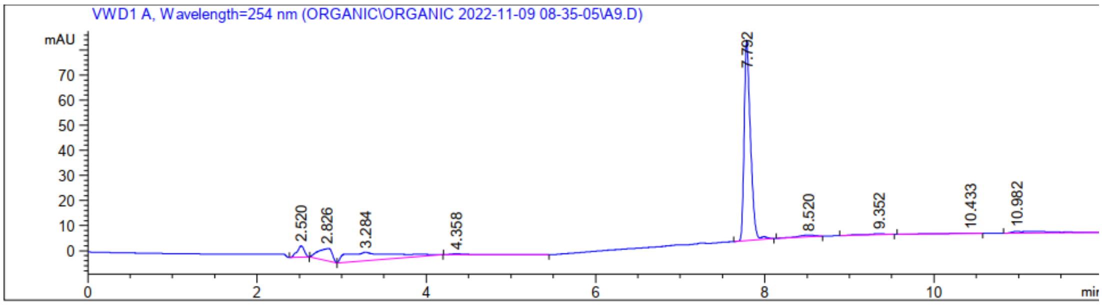

Figure S48. HPLC purity of A12

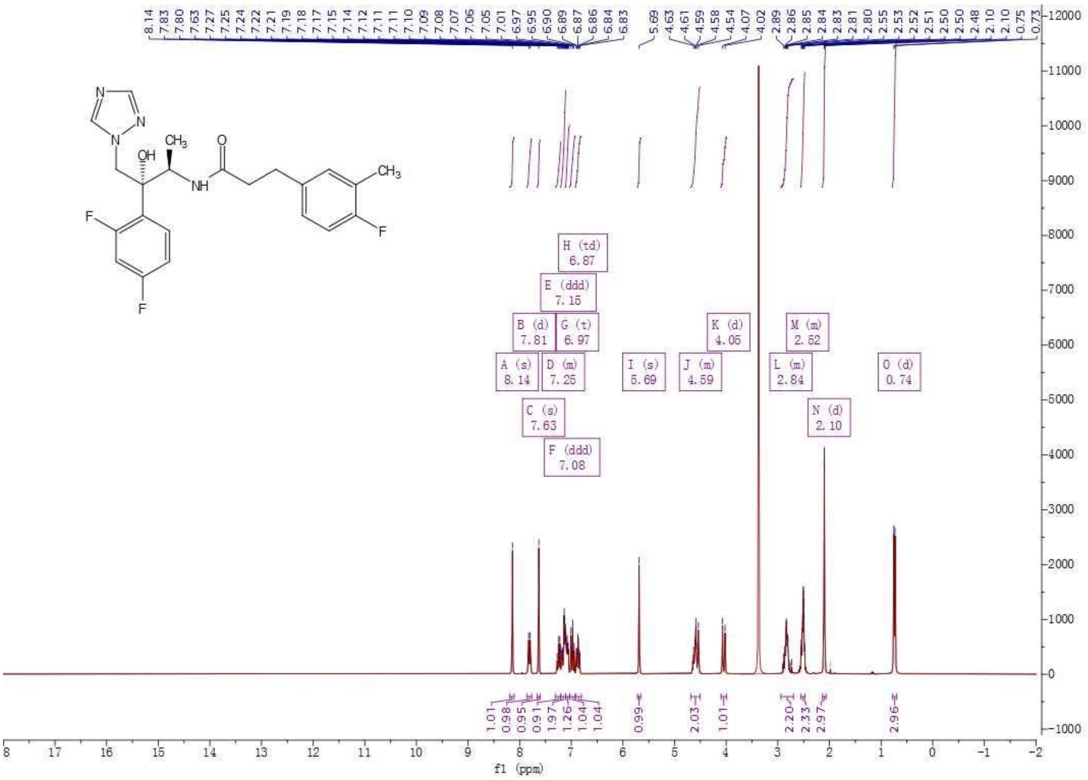

Figure S49. ^{1}H-NMR of A13

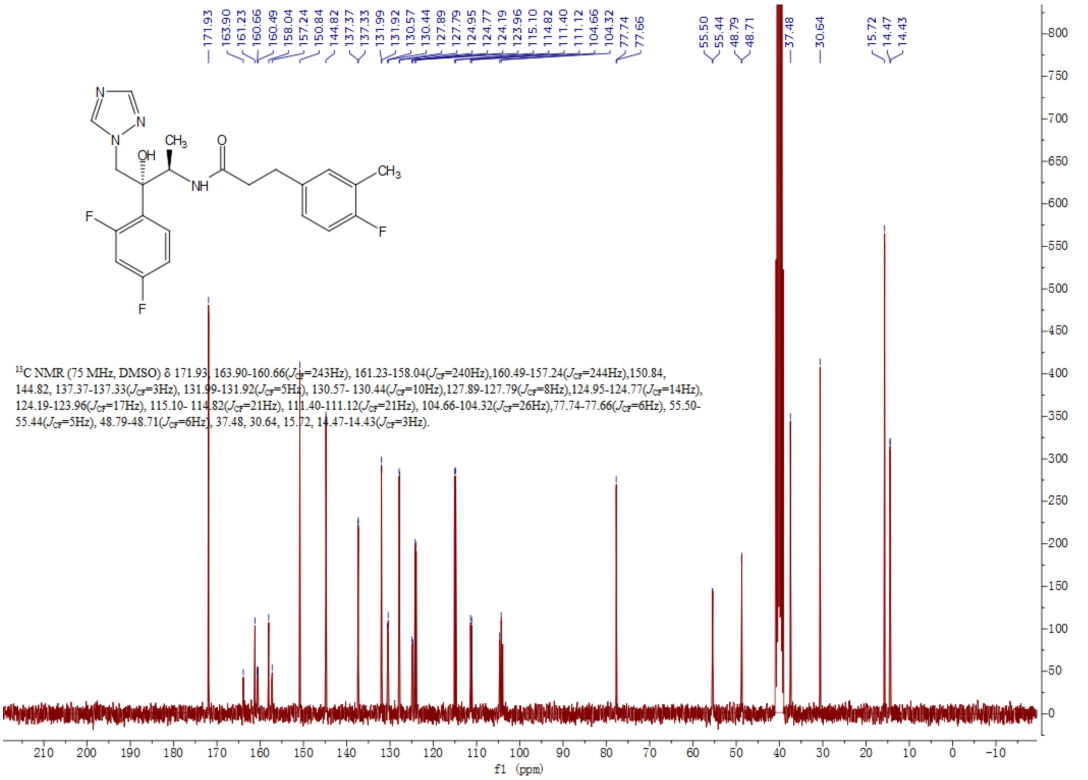

Figure S50. ^{13}C-NMR of A13

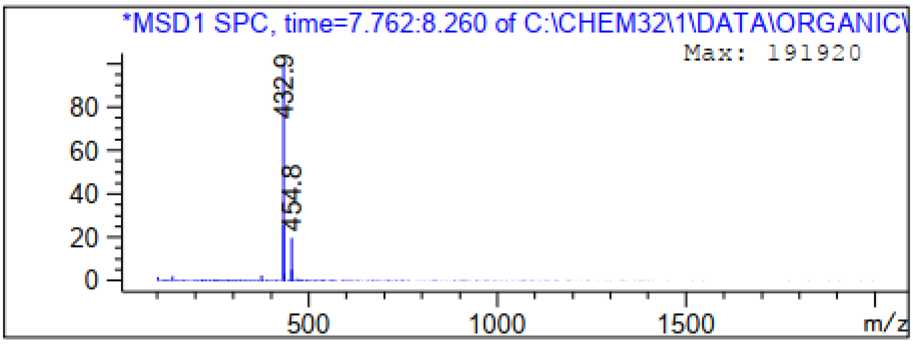

Figure S51. ESI-MS of A13

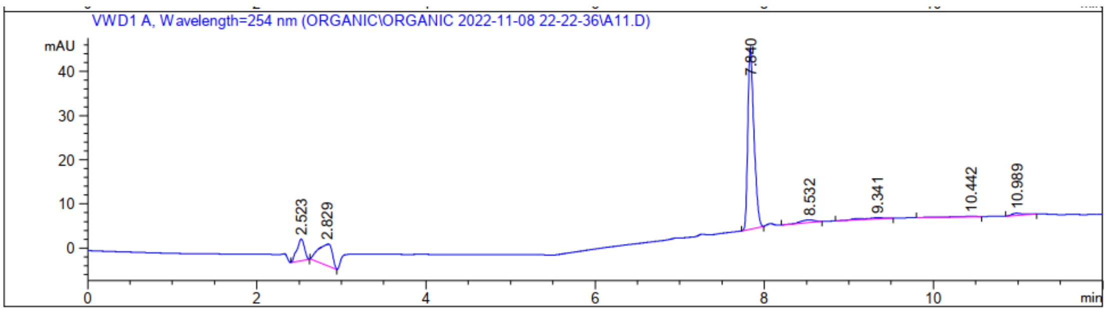

Figure S52. HPLC purity of A13

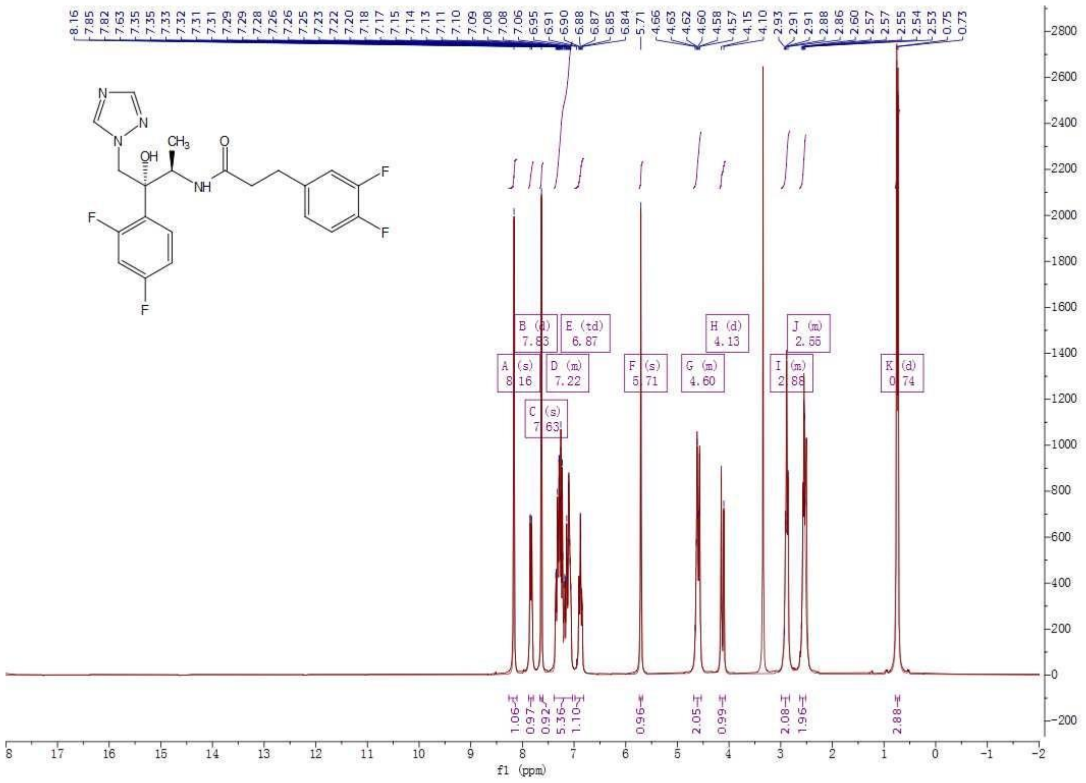

Figure S53. ^{1}H-NMR of A14

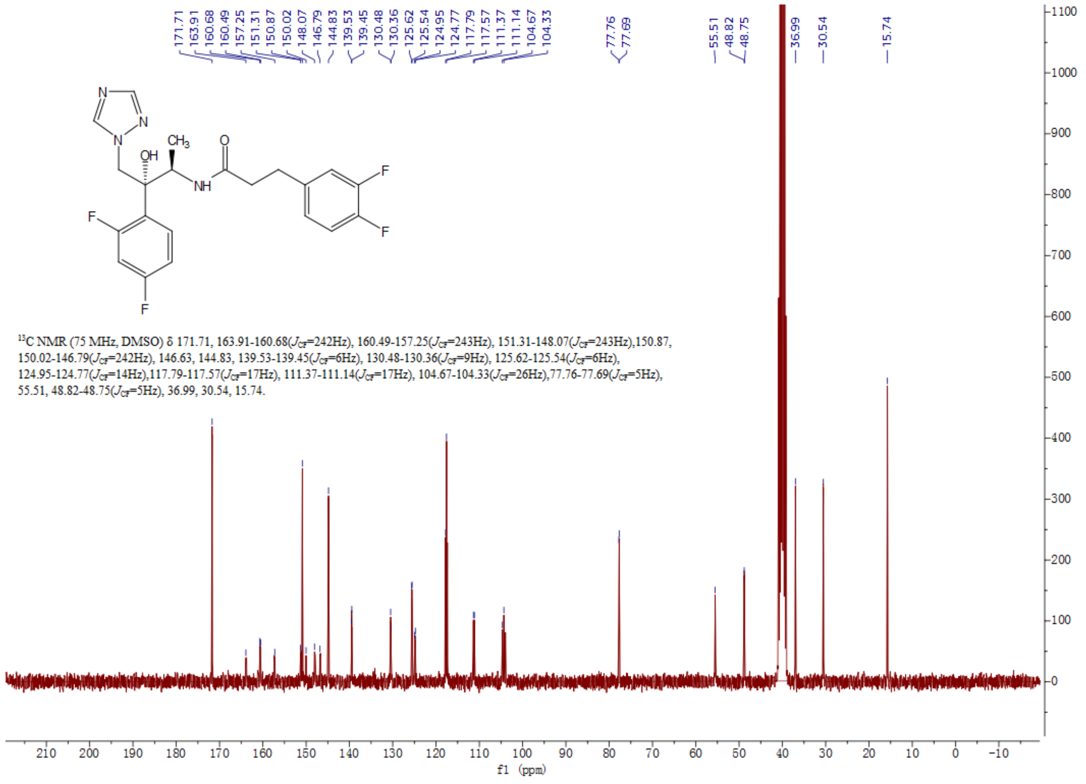

Figure S54. ^{13}C-NMR of A14

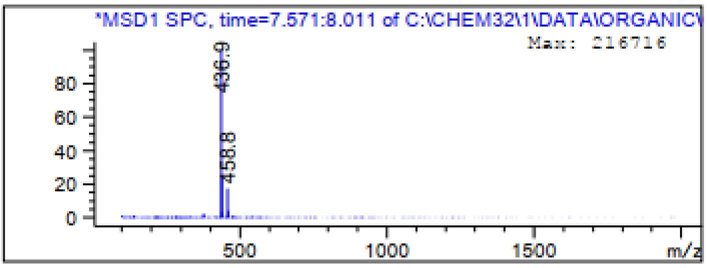

Figure S55. ESI-MS of A14

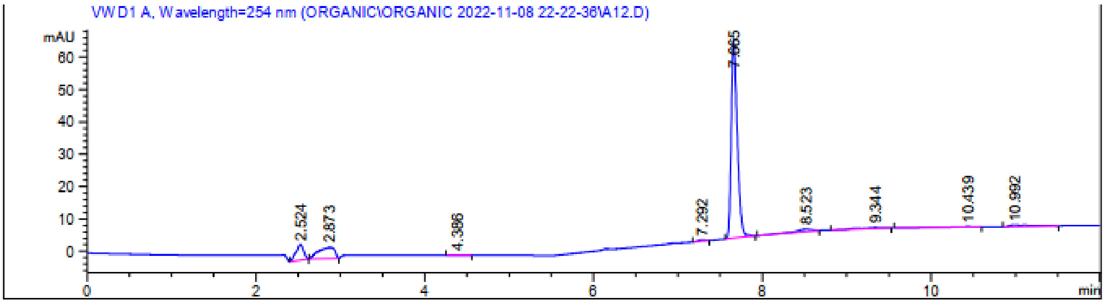

Figure S56. HPLC purity of A14

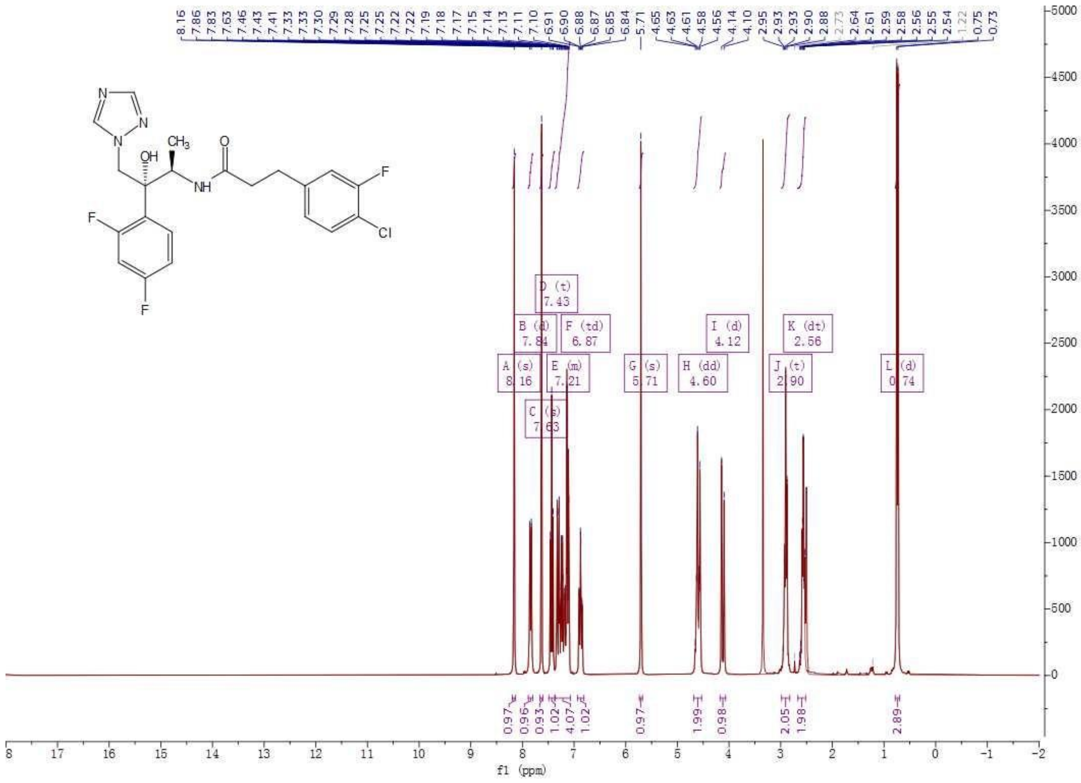

Figure S57. ^{1}H-NMR of A15

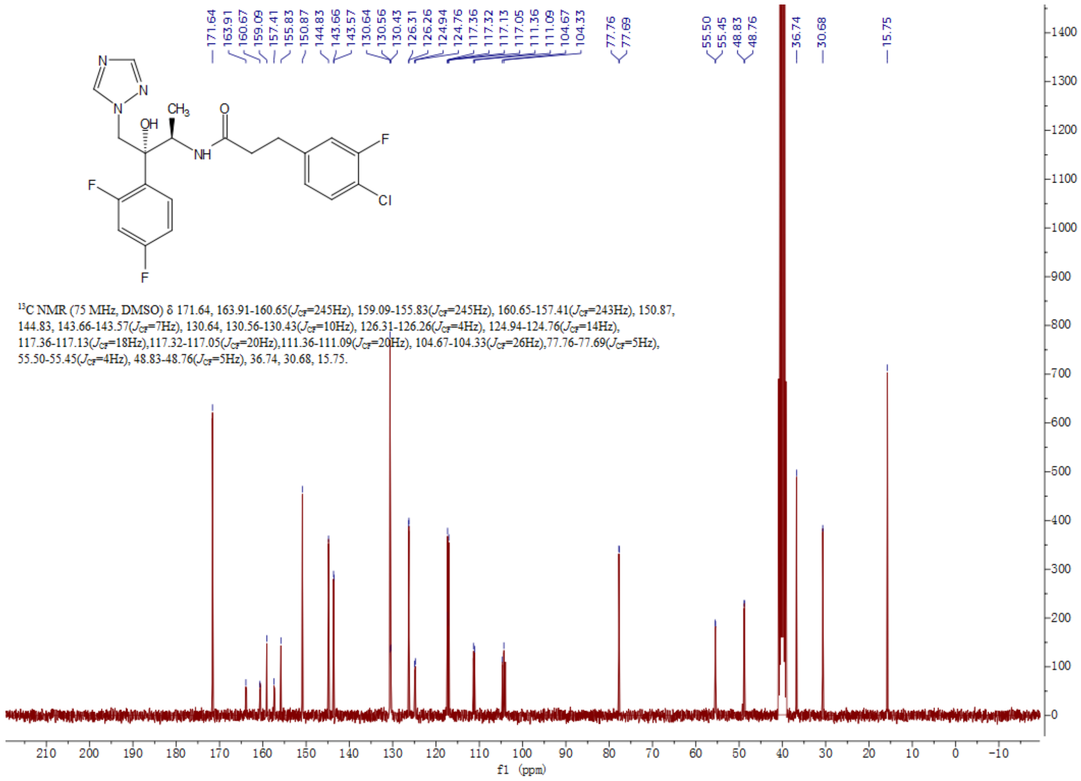

Figure S58. ^{13}C-NMR of A15

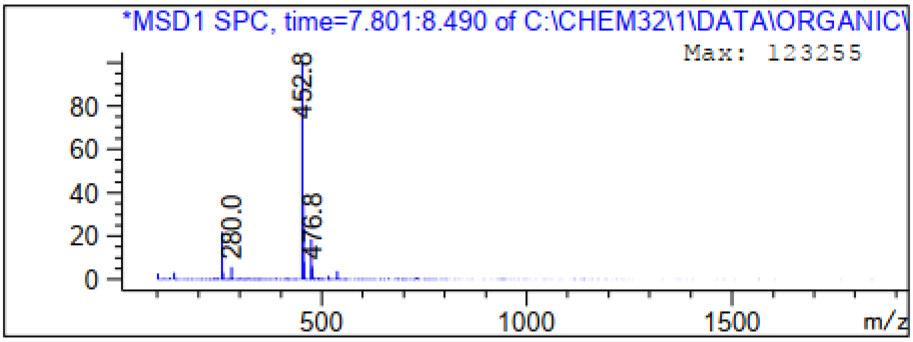

Figure S59. ESI-MS of A15

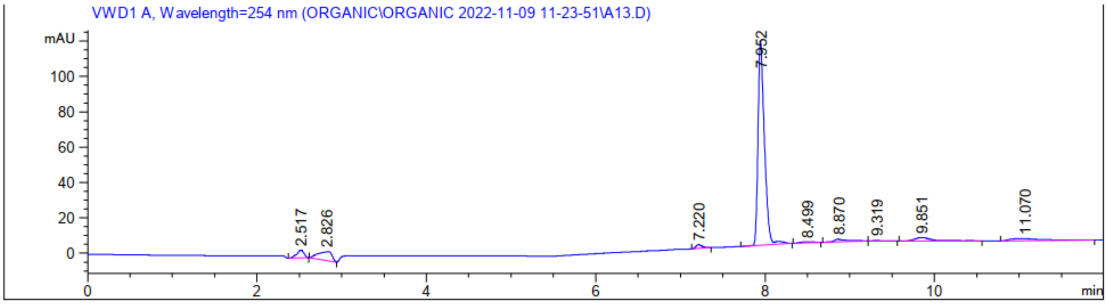

Figure S60. HPLC purity of A15

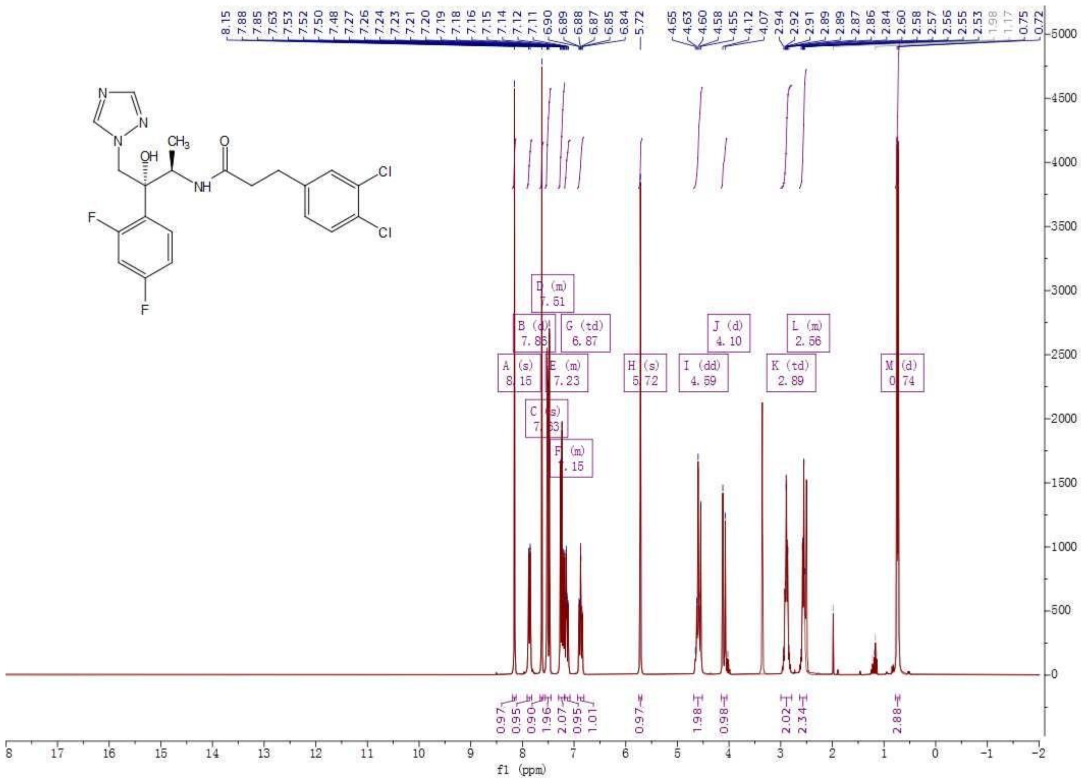

Figure S61. ^{1}H-NMR of A16

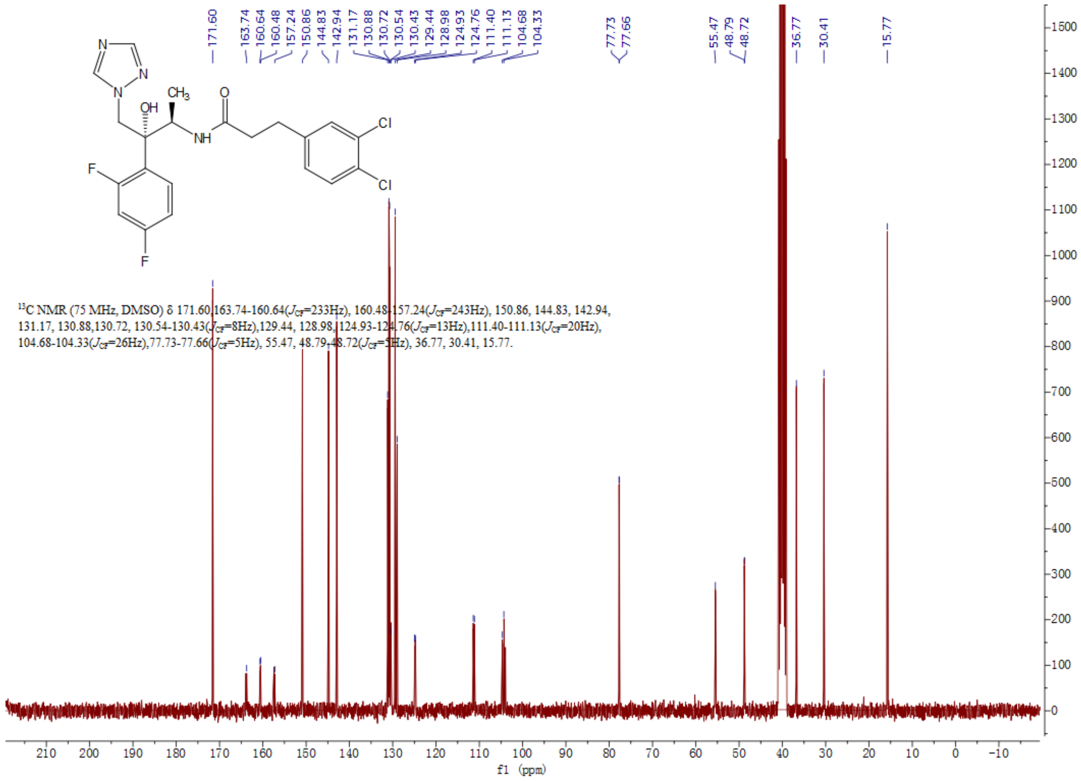

Figure S62. ^{13}C-NMR of A16

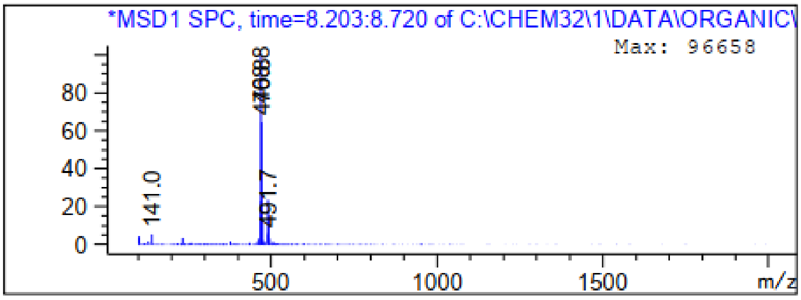

Figure S63. ESI-MS of A16

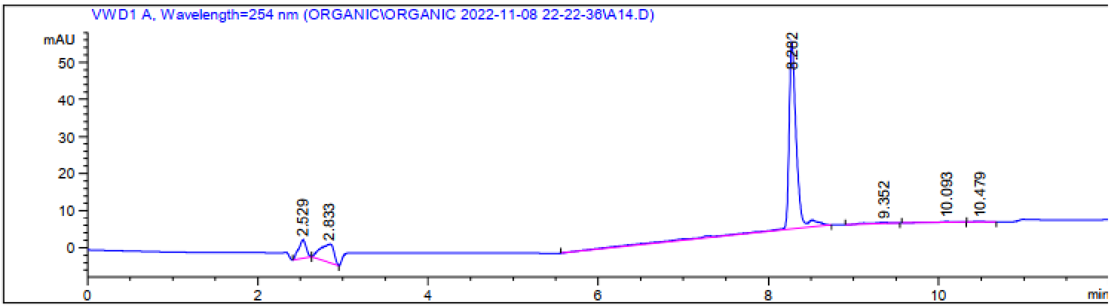

Figure S64. HPLC purity of A16

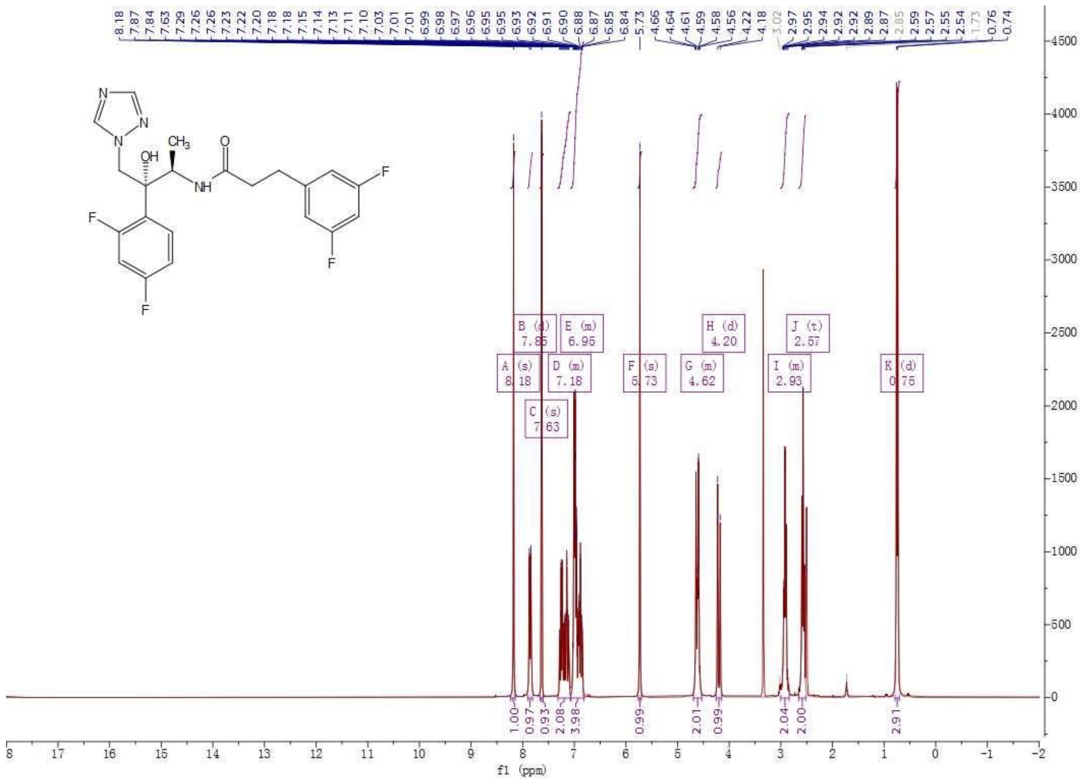

Figure S65. ^{1}H-NMR of A17

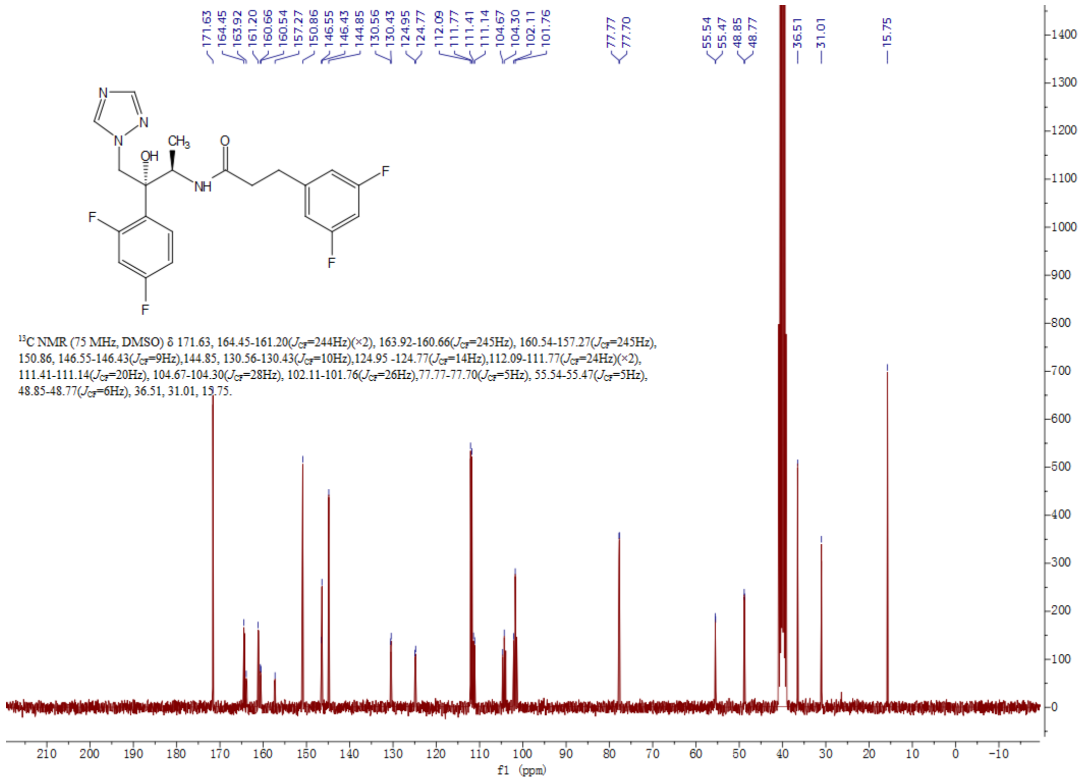

^{13}C NMR (75 MHz, DMSO) δ 171.63, 164.45-161.20(J_{CF}=244Hz)(×2), 163.92-160.66(J_{CF}=245Hz), 160.54-157.27(J_{CF}=245Hz), 150.86, 146.55-146.43(J_{CF}=9Hz),144.85, 130.56-130.43(J_{CF}=10Hz),124.95 -124.77(J_{CF}=14Hz),112.09-111.77(J_{CF}=24Hz)(×2), 111.41-111.14(J_{CF}=20Hz), 104.67-104.30(J_{CF}=28Hz), 102.11-101.76(J_{CF}=26Hz),77.77-77.70(J_{CF}=5Hz), 55.54-55.47(J_{CF}=5Hz), 48.85-48.77(J_{CF}=6Hz), 36.51, 31.01, 15.75.

Figure S66. ^{13}C-NMR of A17

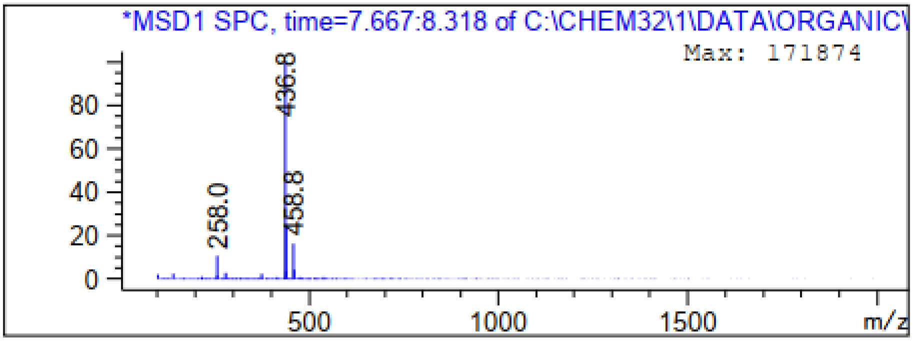

Figure S67. ESI-MS of A17

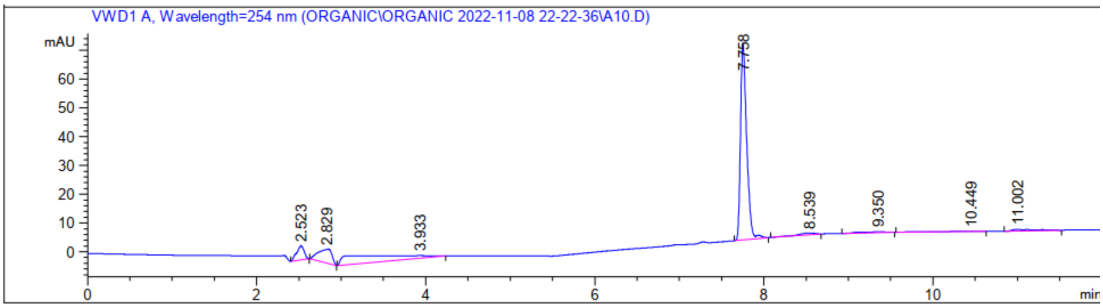

Figure S68. HPLC purity of A17

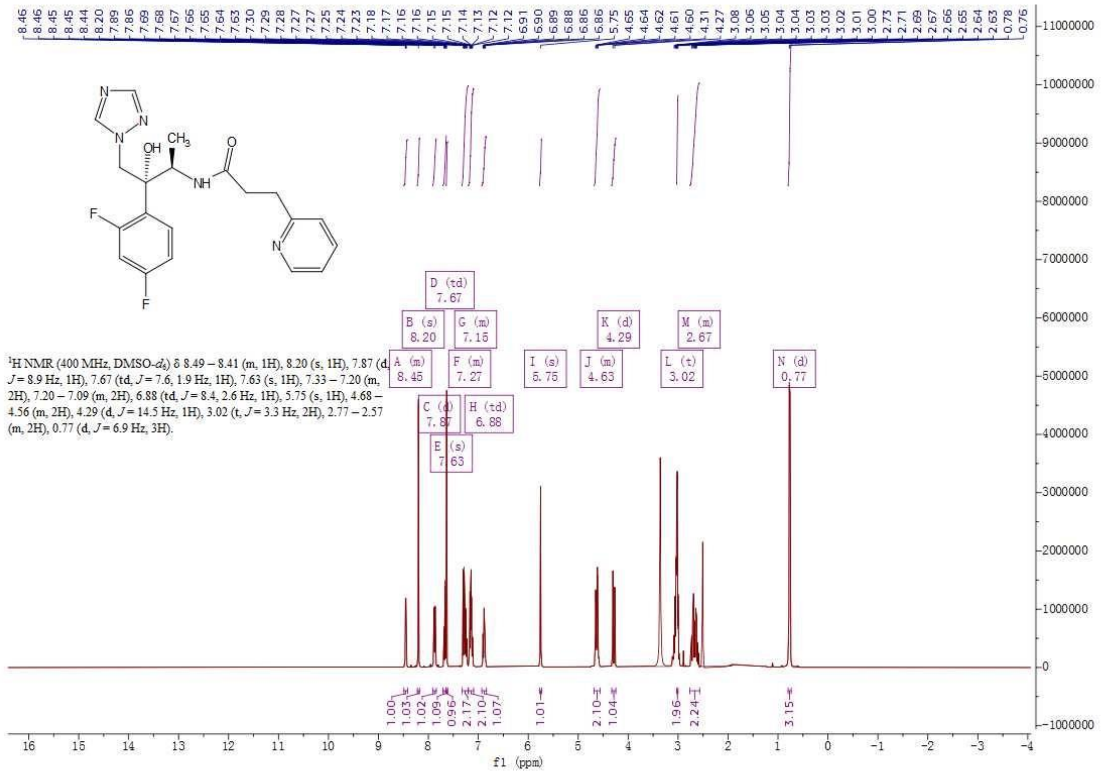

Figure S69. ^{1}H-NMR of A18

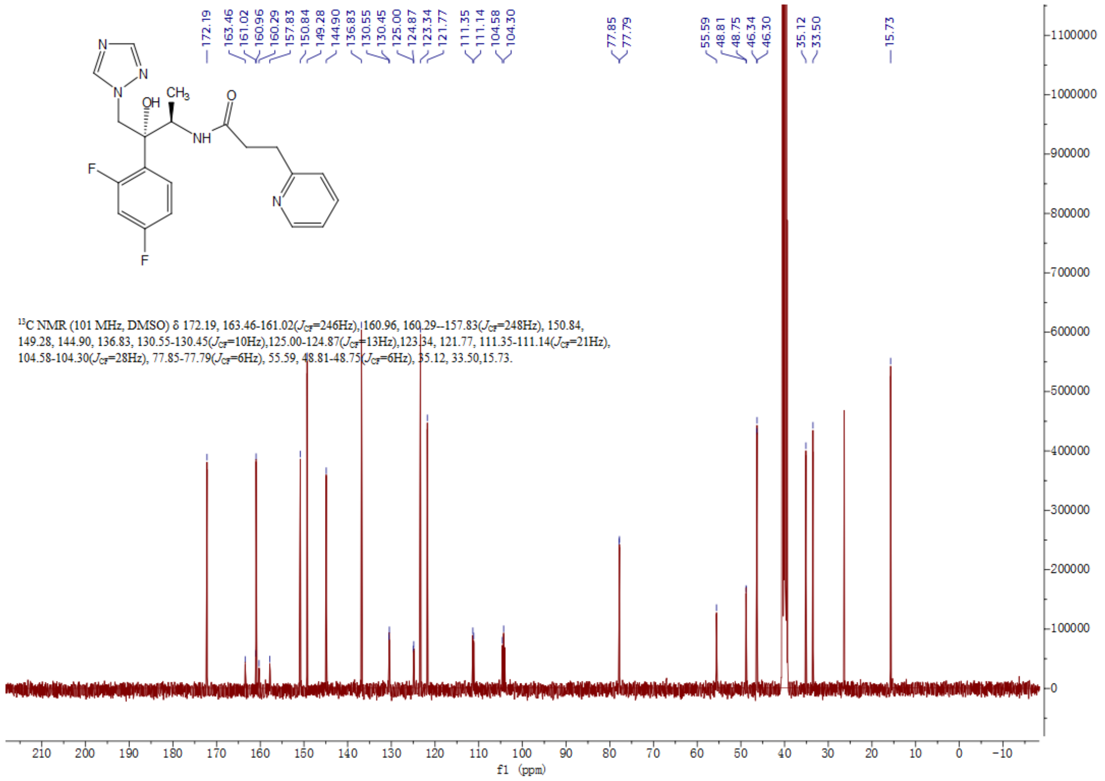

Figure S70. ^{13}C-NMR of A18

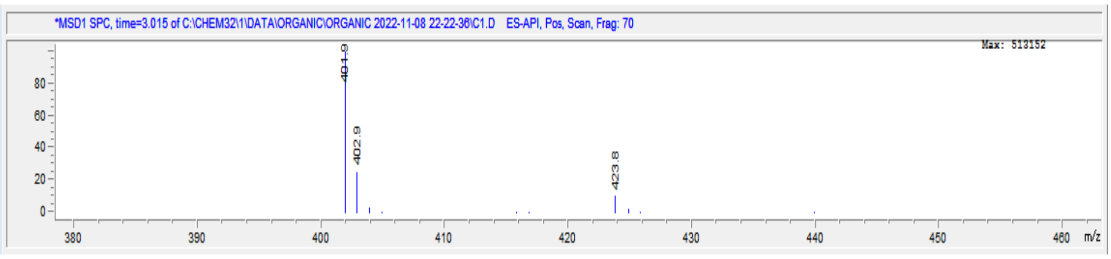

Figure S71. ESI-MS of A18

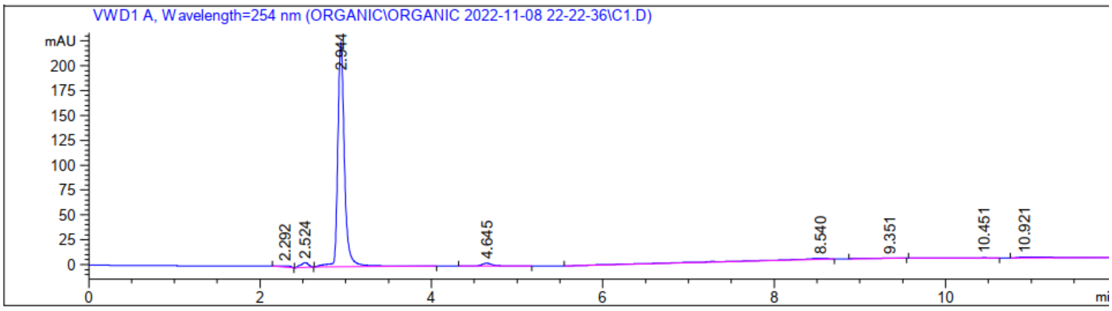

Figure S72. HPLC purity of A18

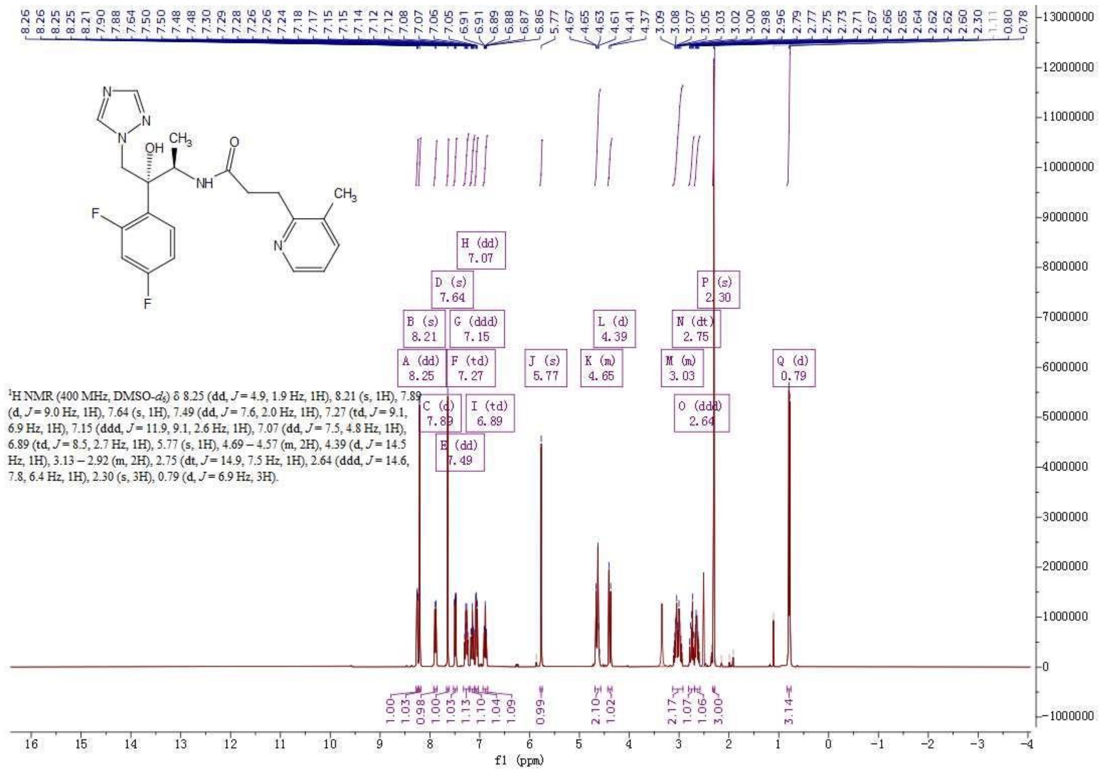

Figure S73. ^{1}H-NMR of A19

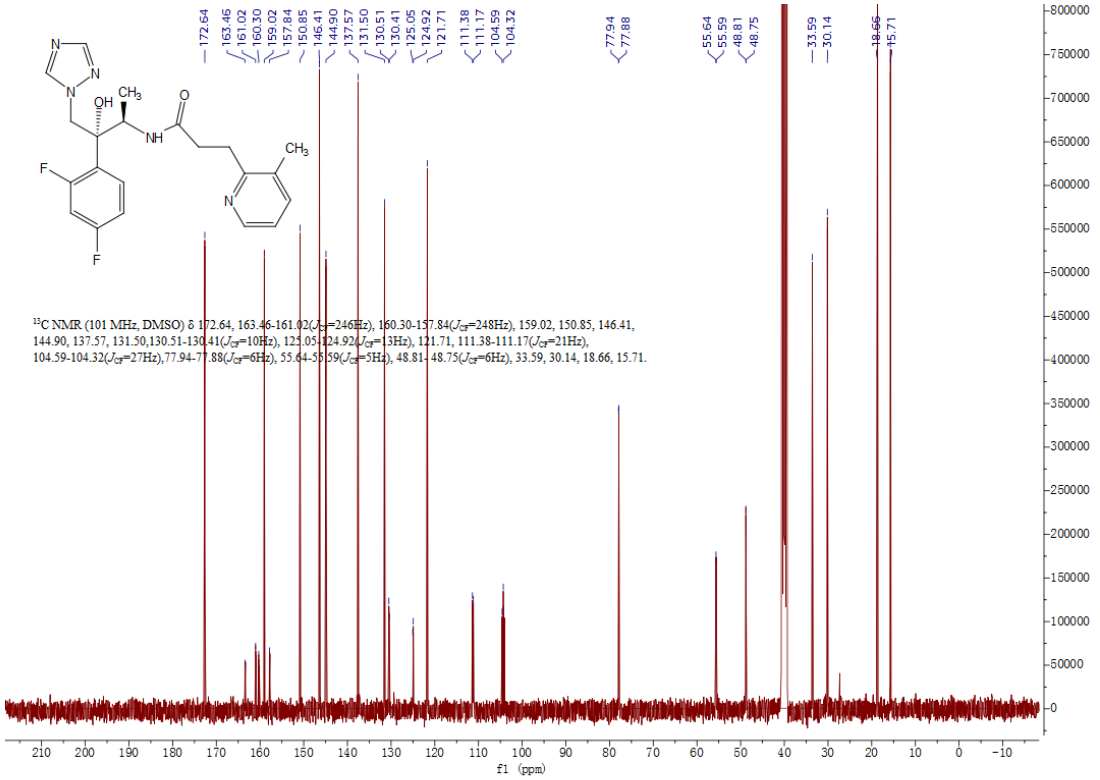

Figure S74. ^{13}C-NMR of A19

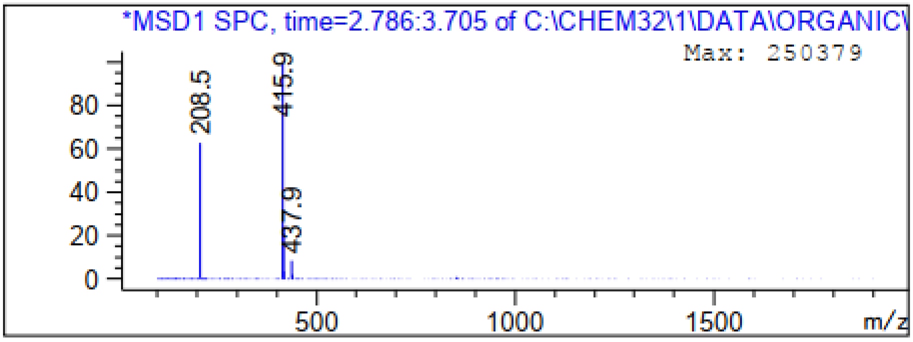

Figure S75. ESI-MS of A19

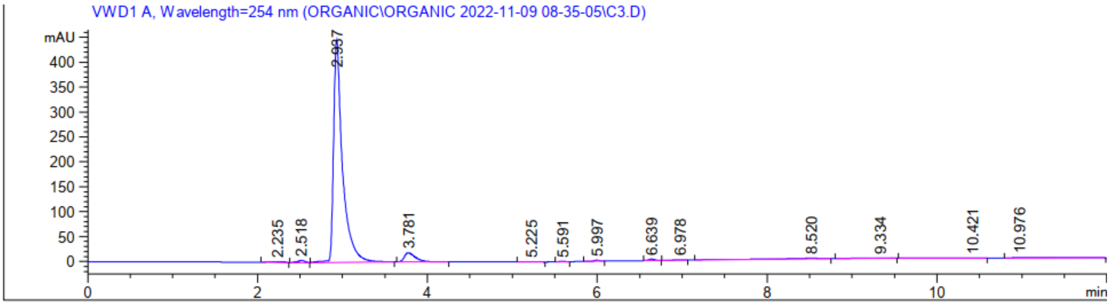

Figure S76. HPLC purity of A19

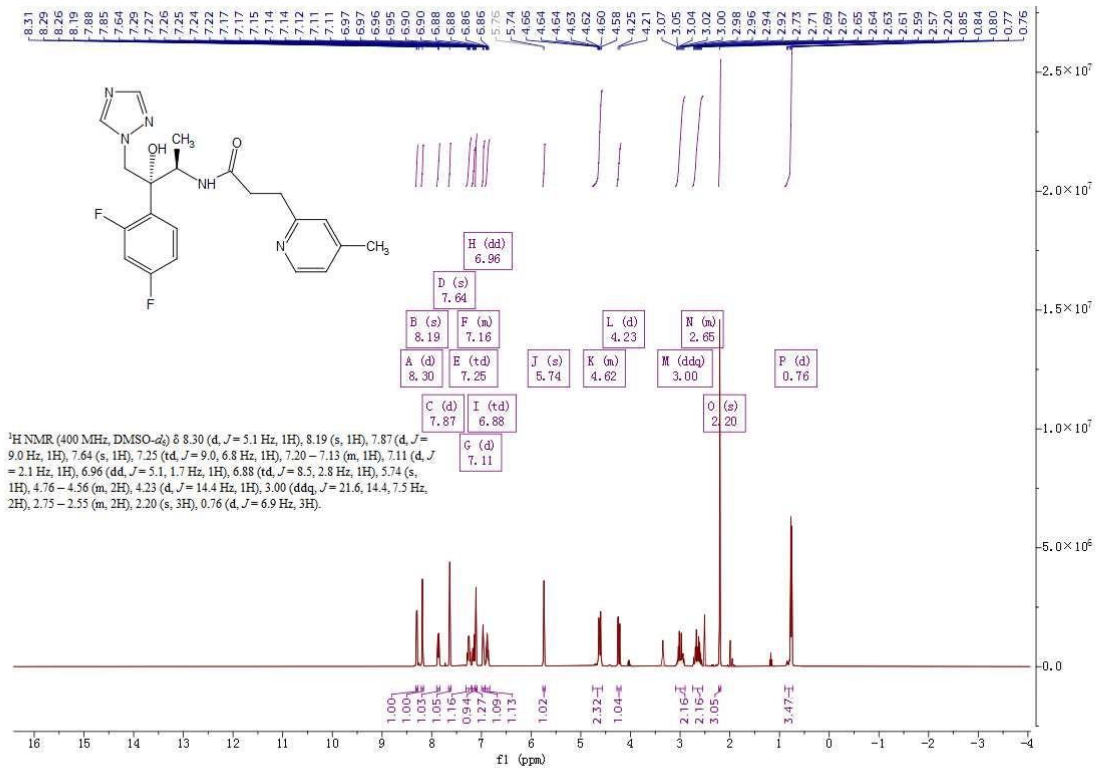

Figure S77. ^{1}H-NMR of A20

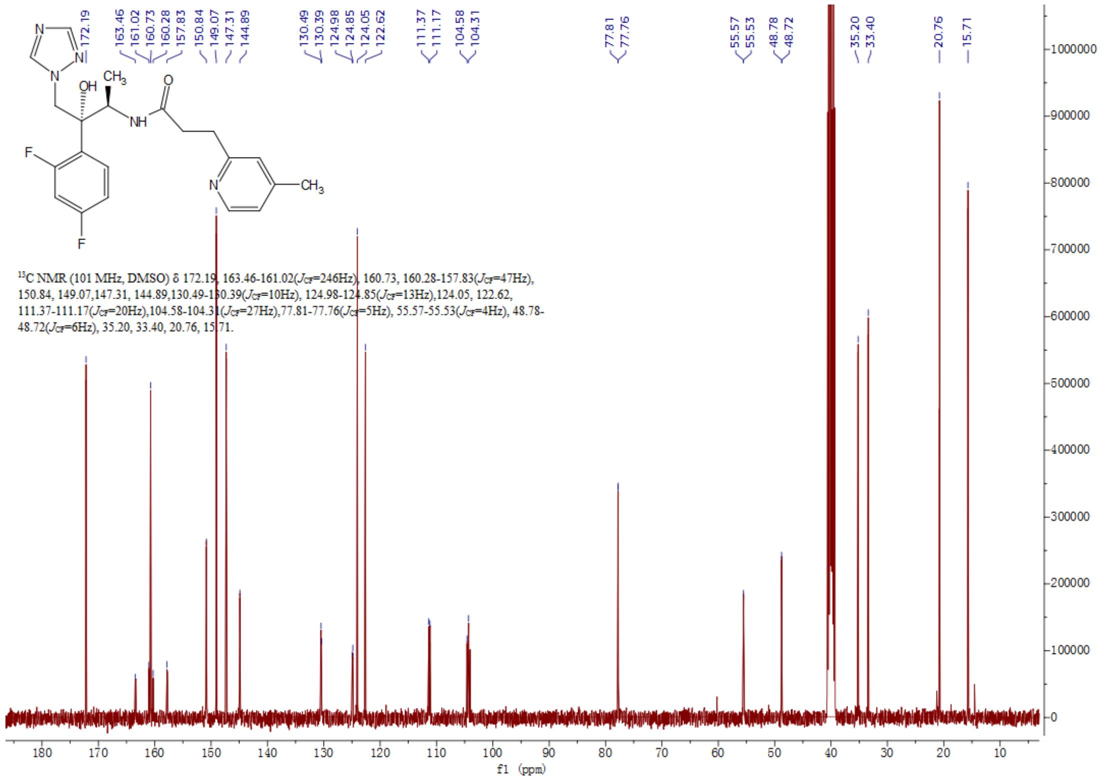

Figure S78. ^{13}C-NMR of A20

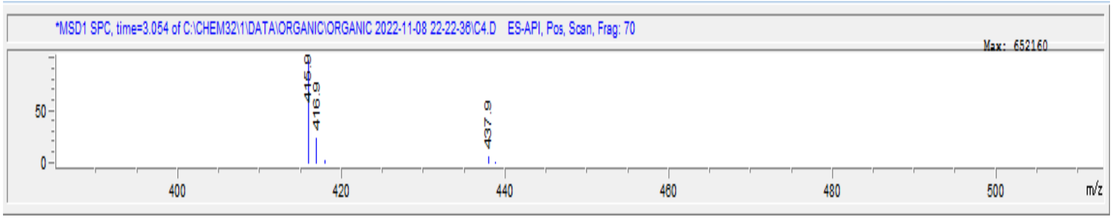

Figure S79. ESI-MS of A20

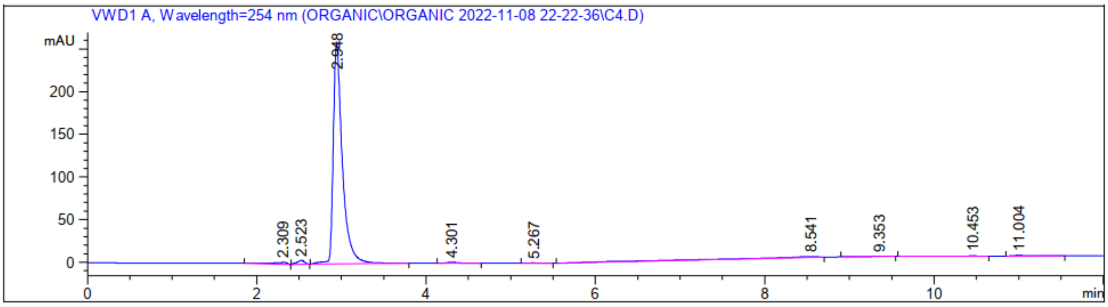

Figure S80. HPLC purity of A20

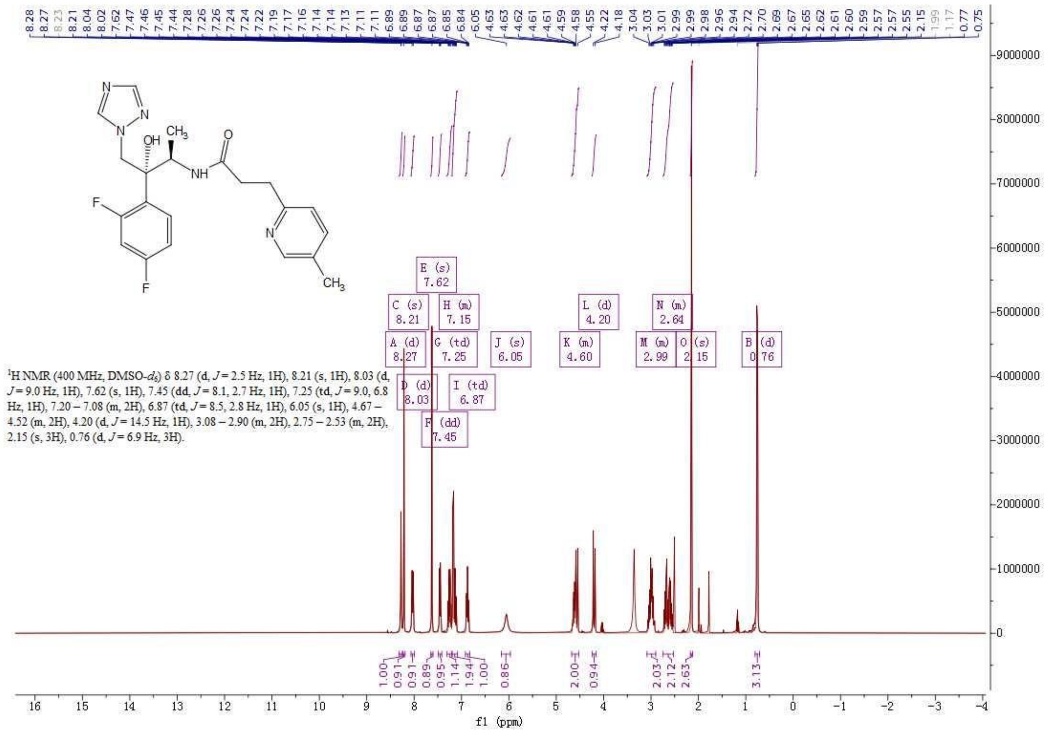

Figure S81. ¹H-NMR of A21

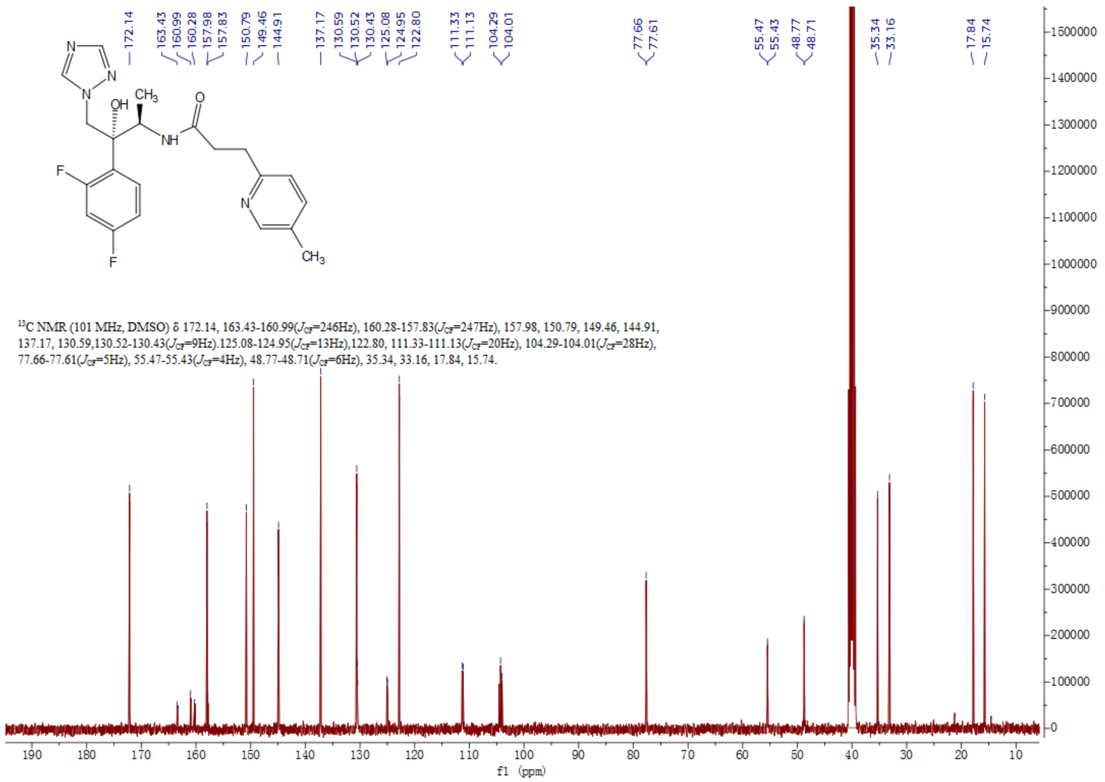

Figure S82. ¹³C-NMR of A21

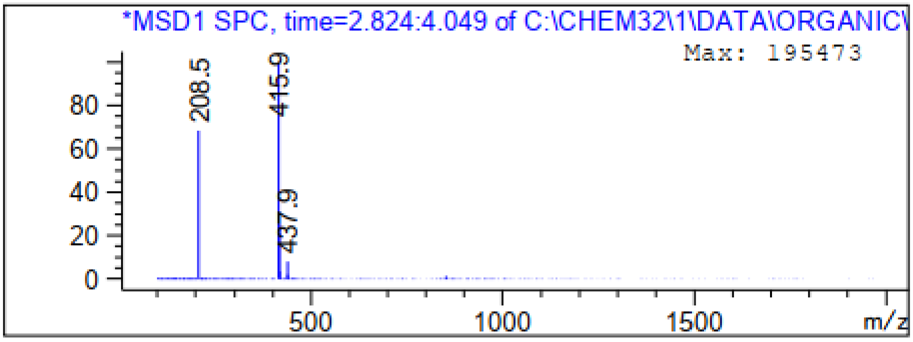

Figure S83. ESI-MS of A21

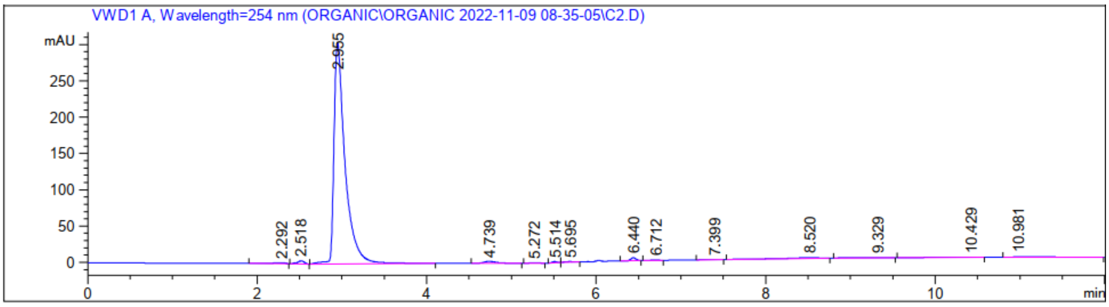

Figure S84. HPLC purity of A21

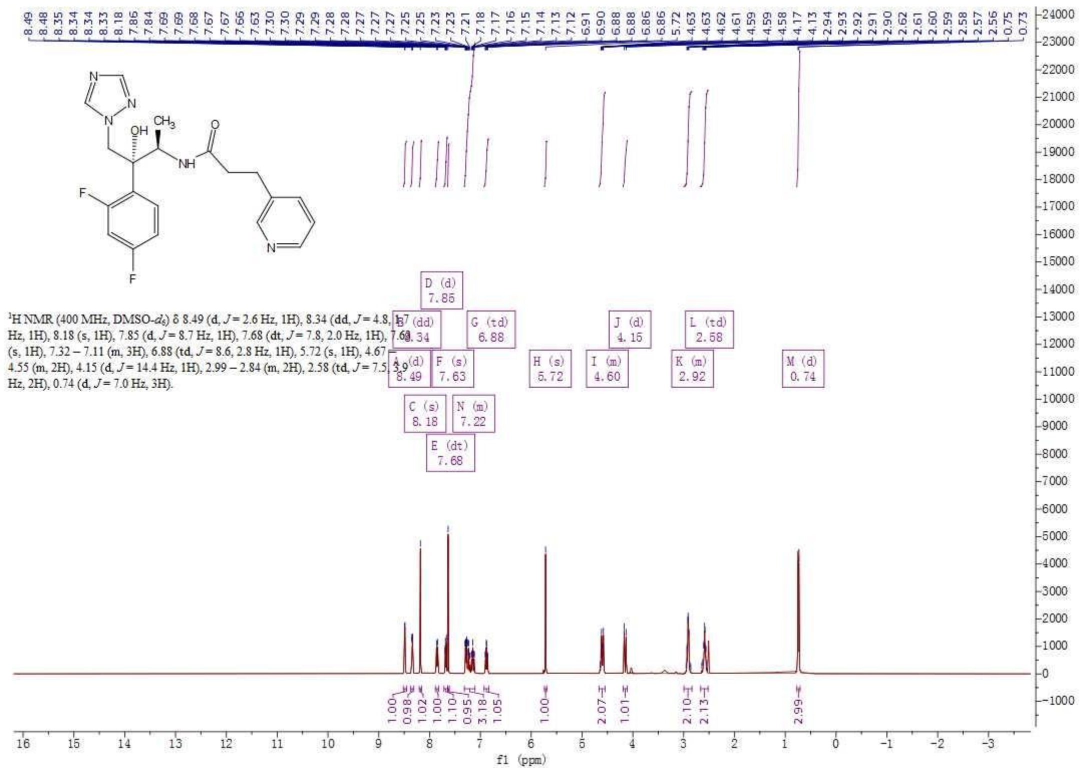

Figure S85. ^{1}H-NMR of A22

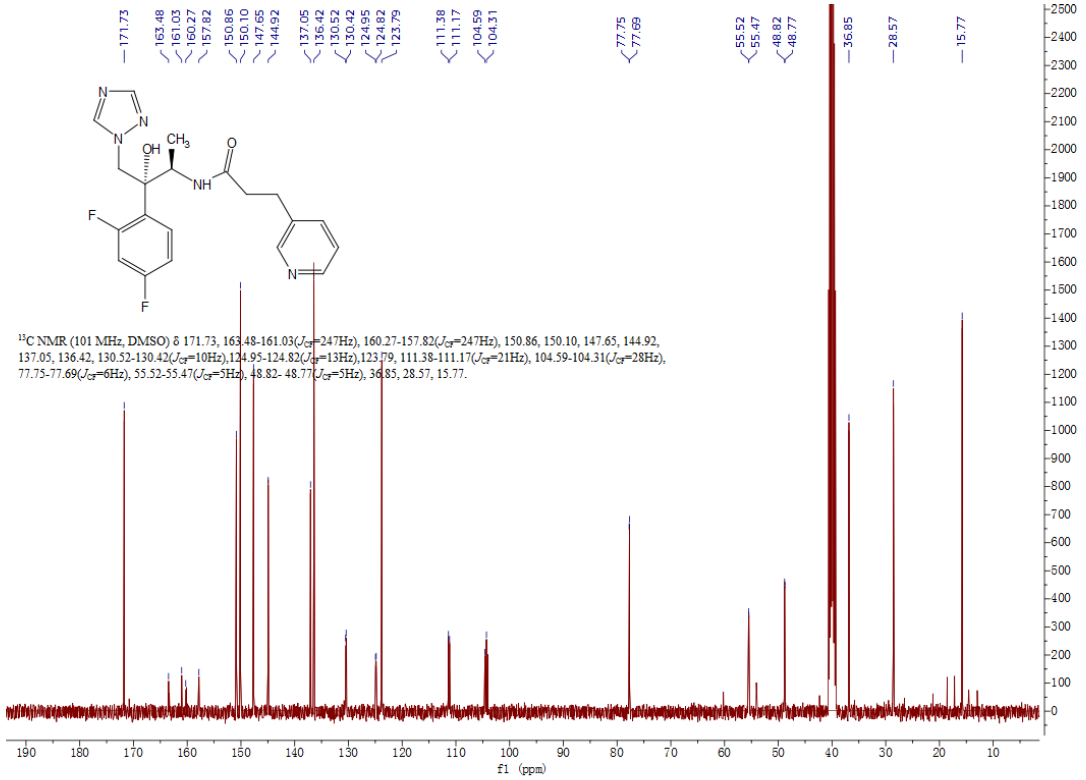

Figure S86. ^{13}C-NMR of A22

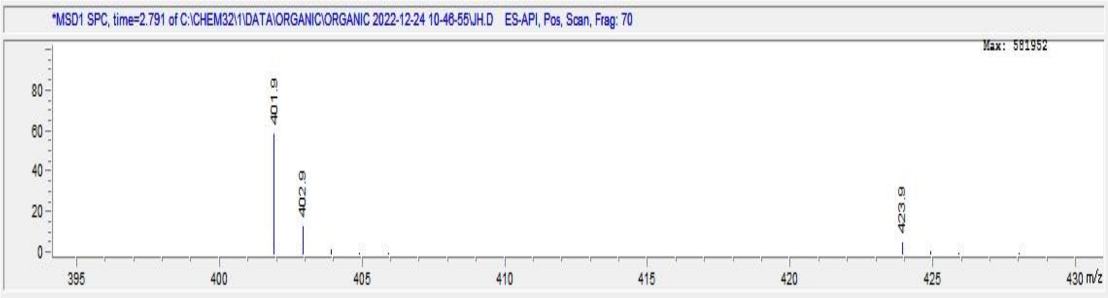

Figure S87. ESI-MS of A22

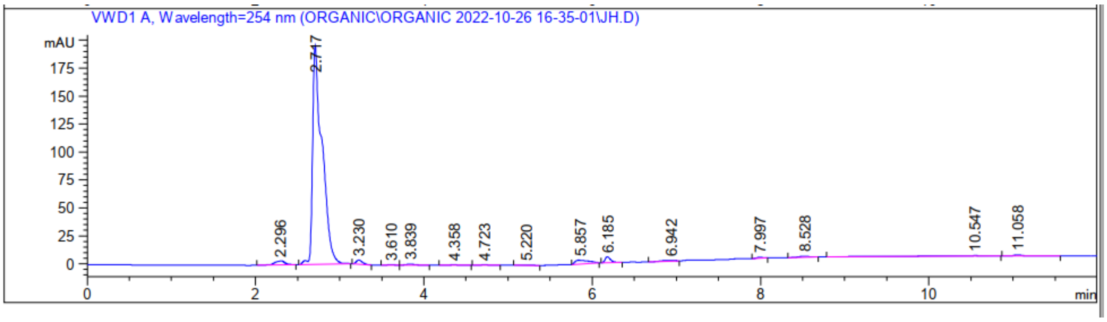

Figure S88. HPLC purity of A22

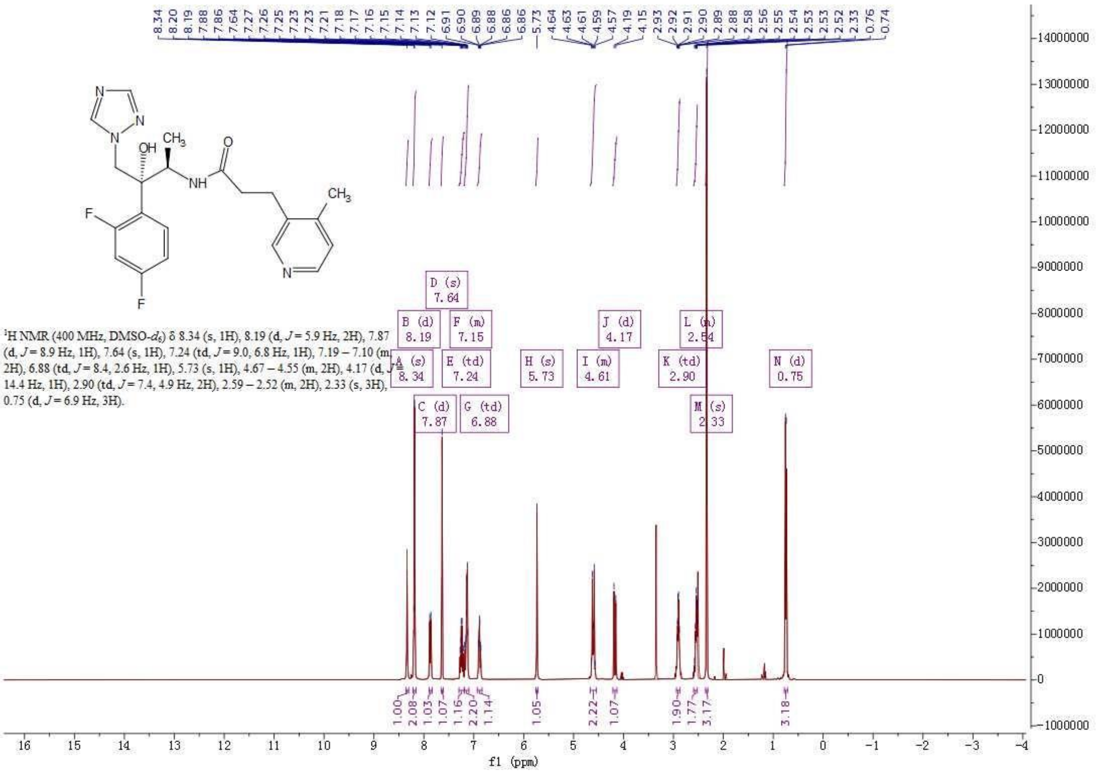

Figure S89. ¹H-NMR of A23

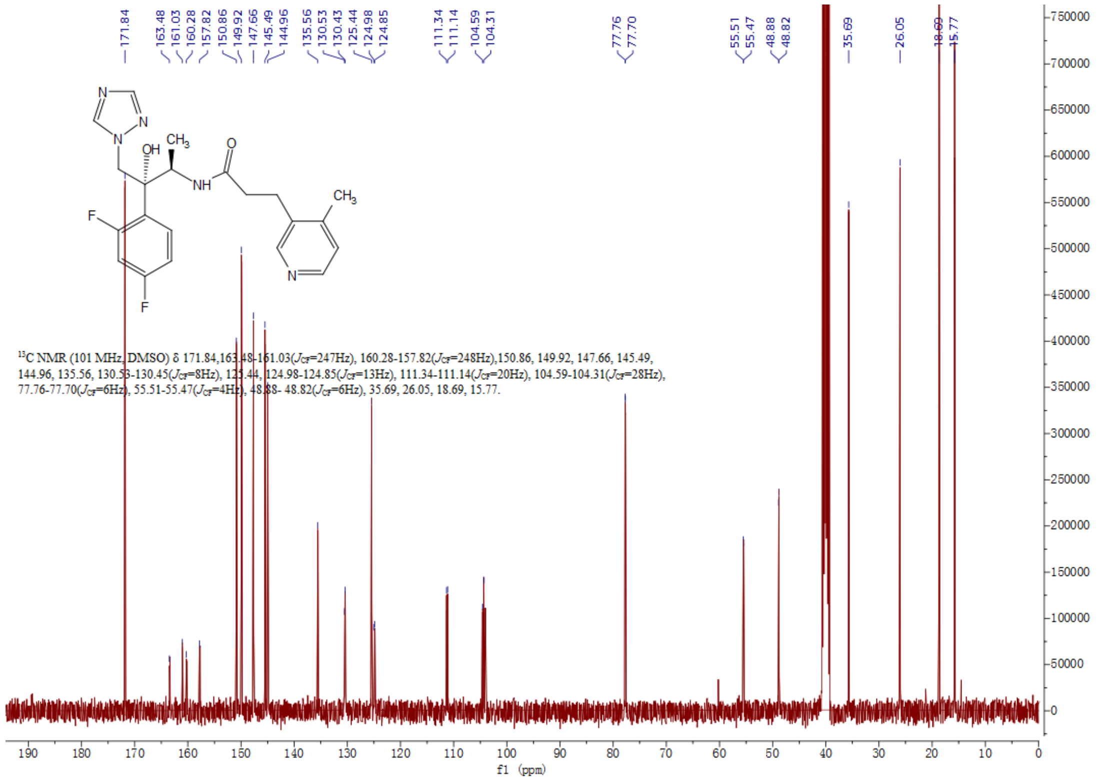

Figure S90. ¹³C-NMR of A23

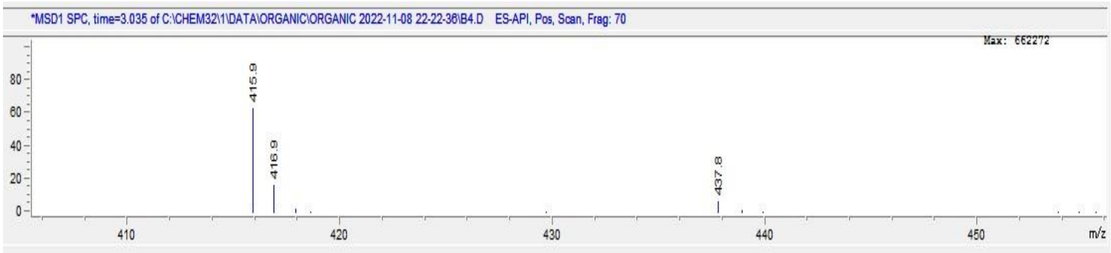

Figure S91. ESI-MS of A23

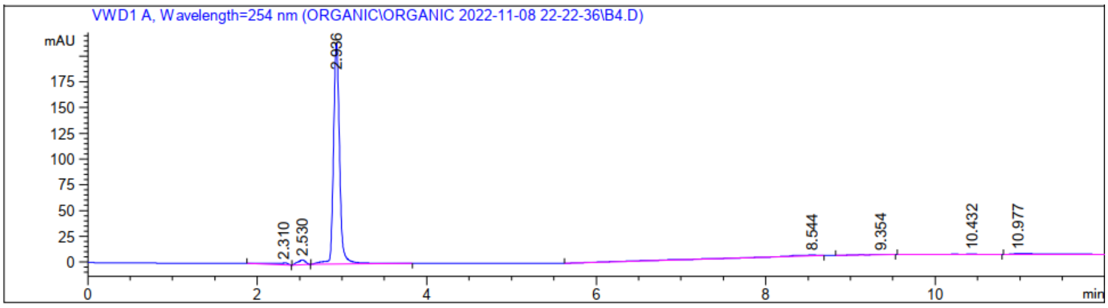

Figure S92. HPLC purity of A23

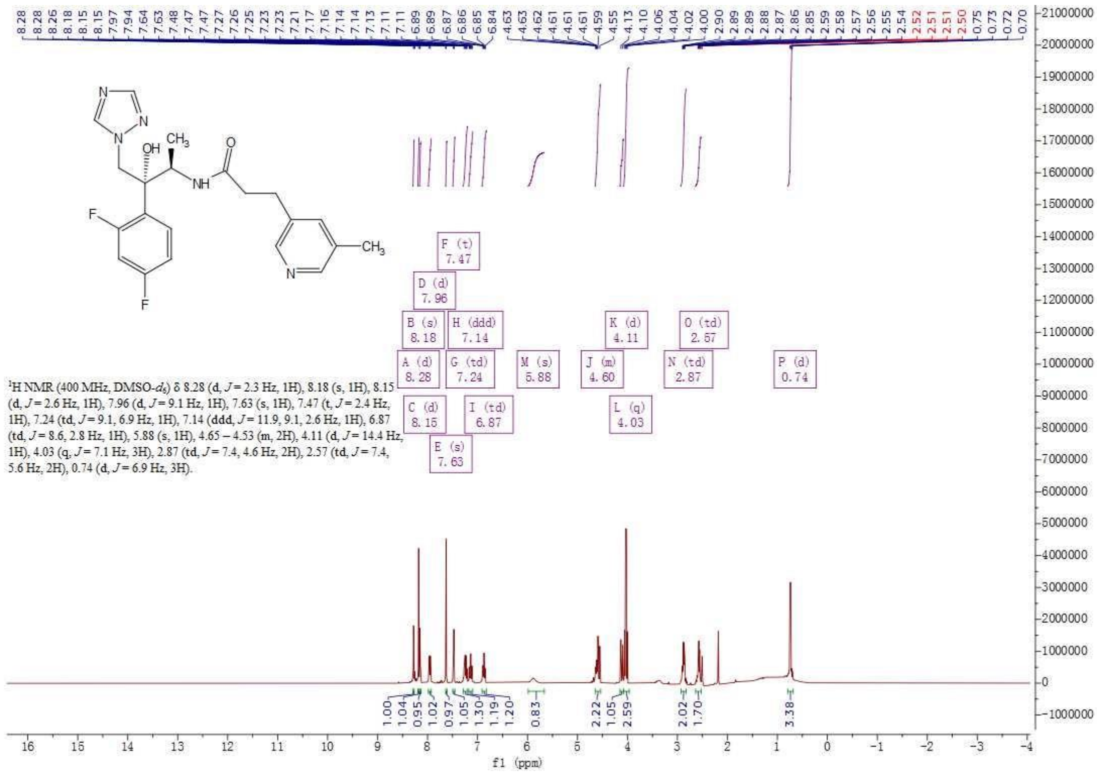

Figure S93. ^{1}H-NMR of A24

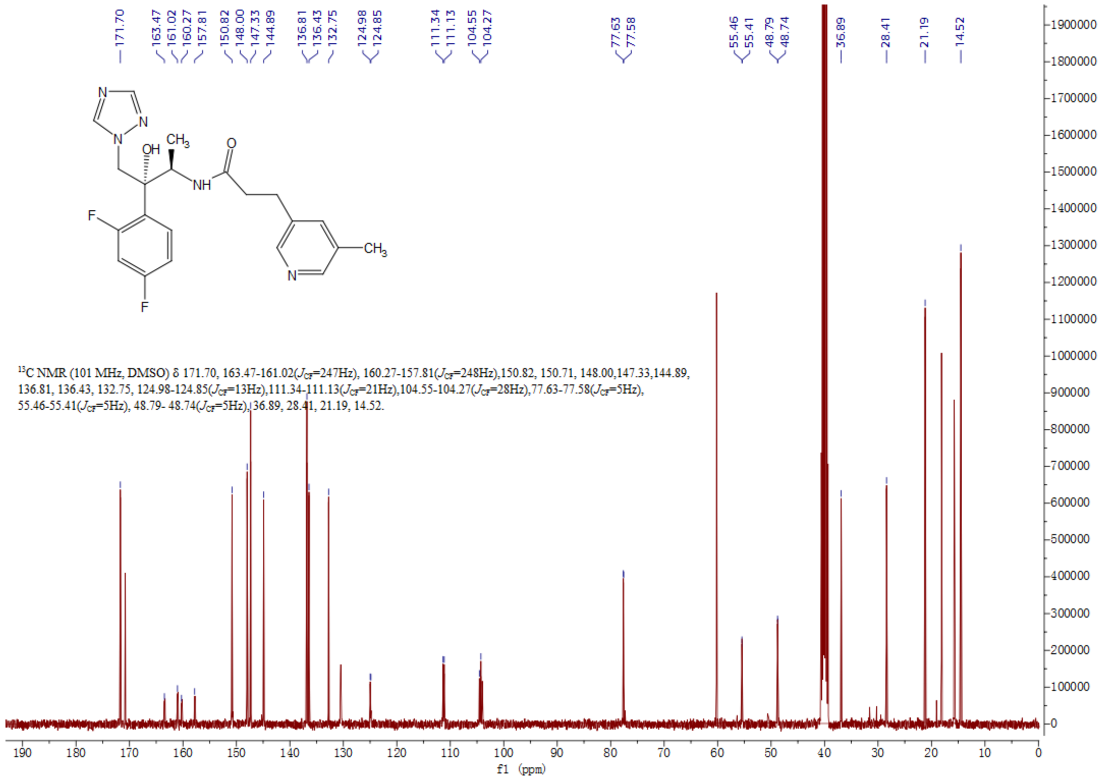

Figure S94. ^{13}C-NMR of A24

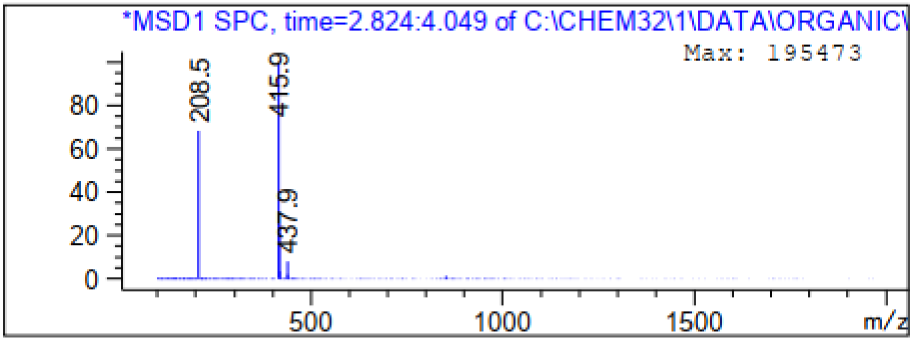

Figure S95. ESI-MS of A24

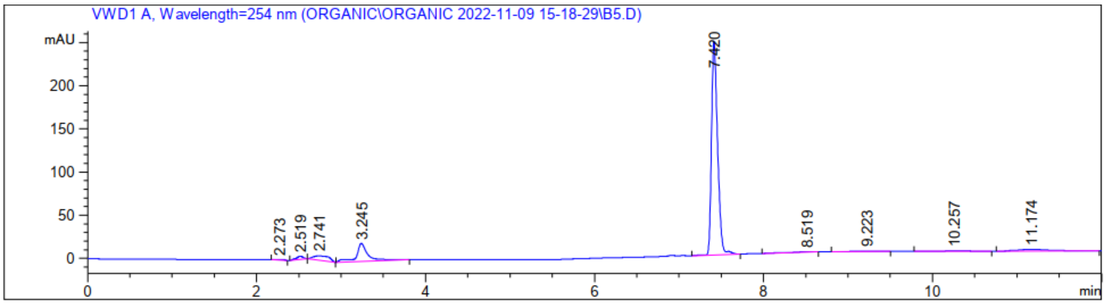

Figure S96. HPLC purity of A24

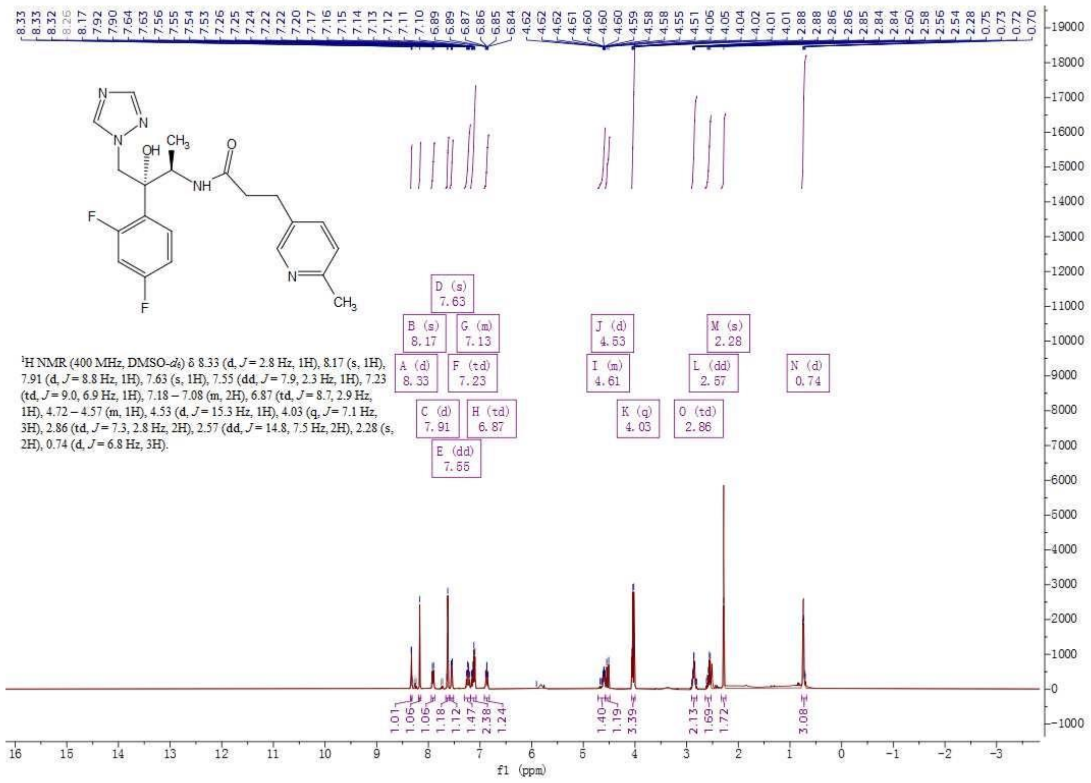

Figure S97. ^{1}H-NMR of A25

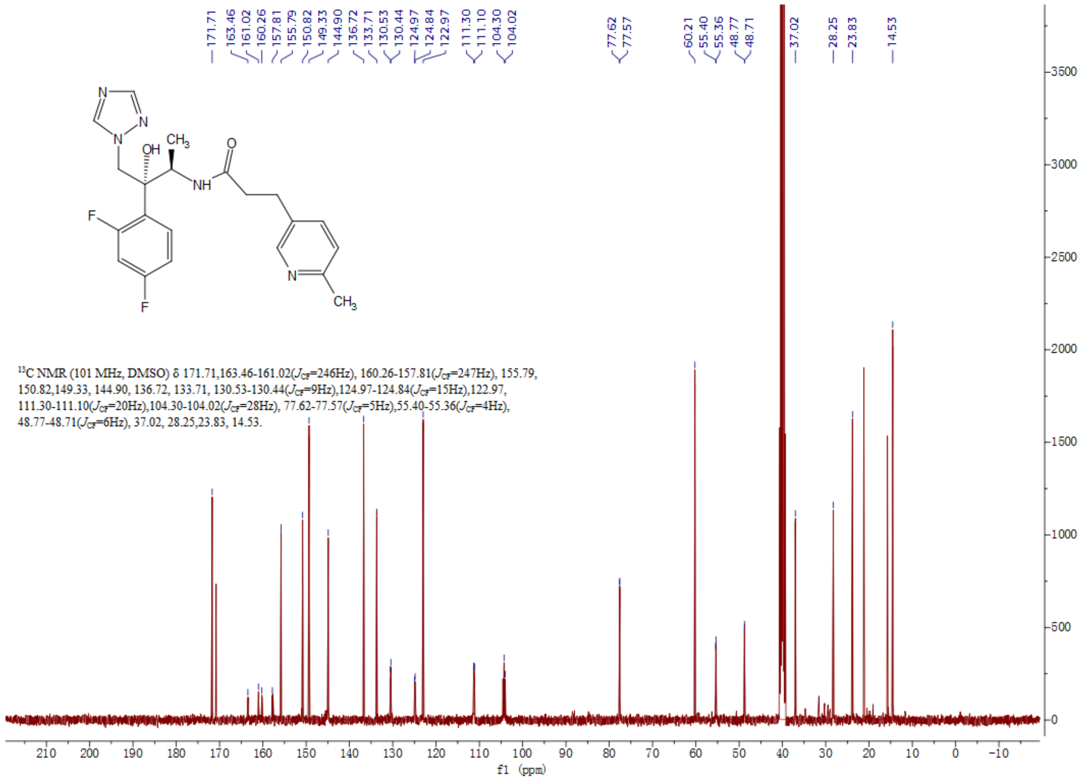

Figure S98. ^{13}C-NMR of A25

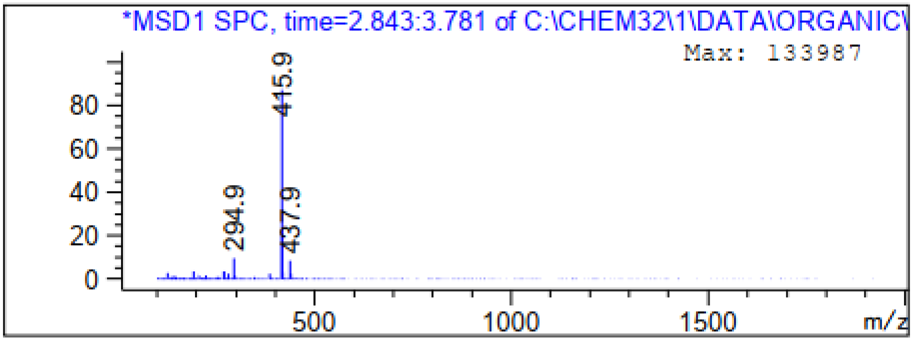

Figure S99. ESI-MS of A25

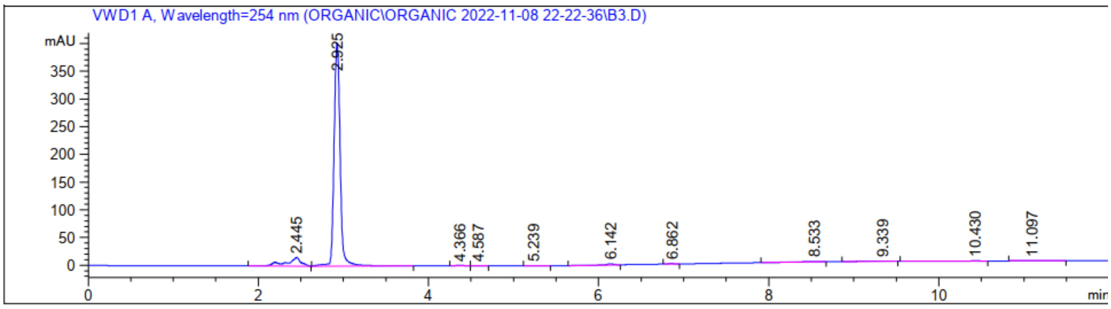

Figure S100. HPLC purity of A25

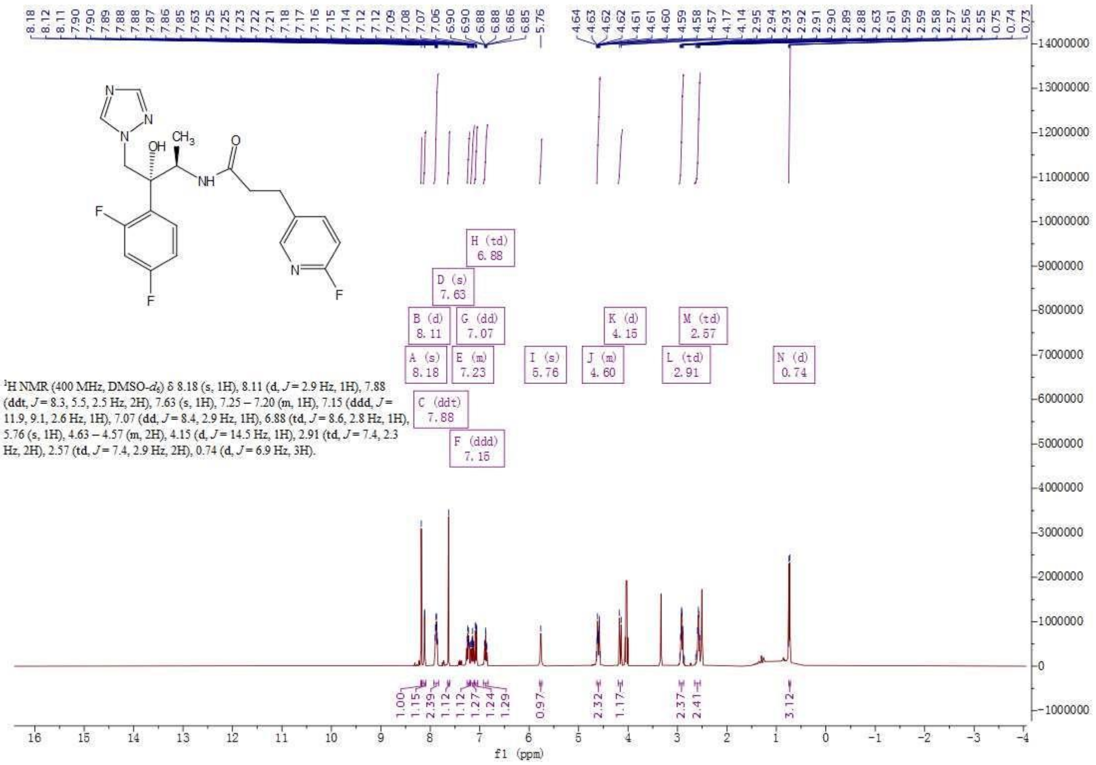

Figure S101. ^{1}**H-NMR of A26**

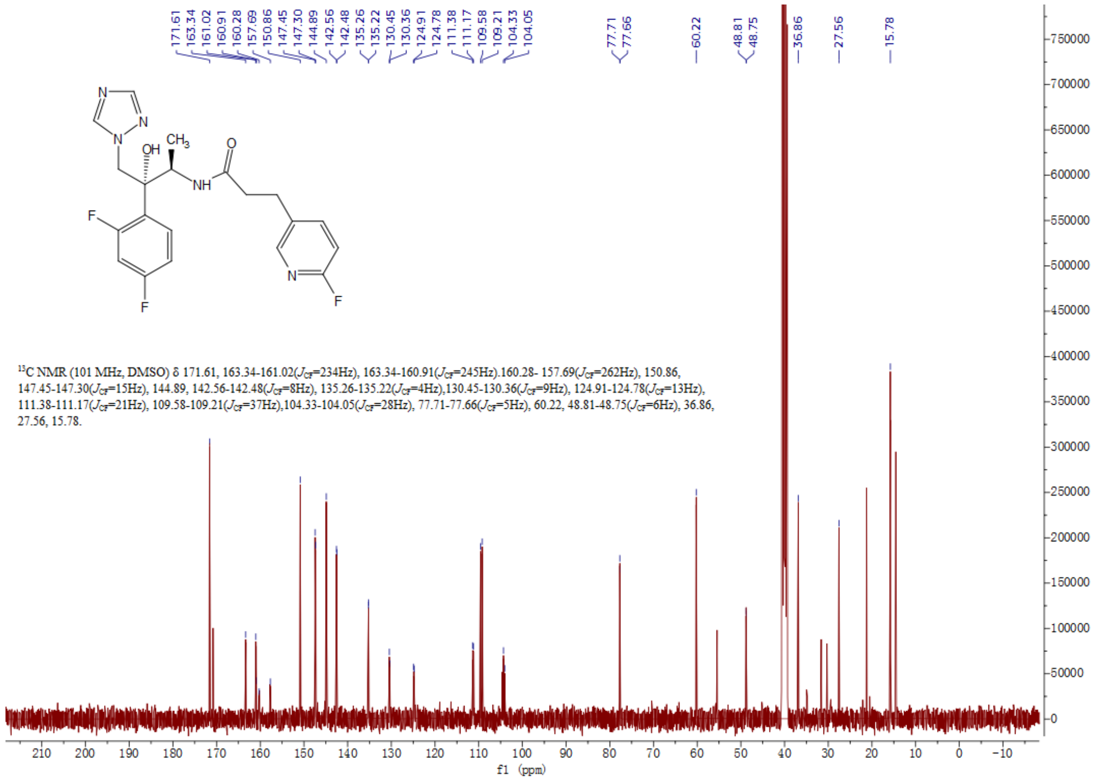

Figure S102. ^{13}**C-NMR of A26**

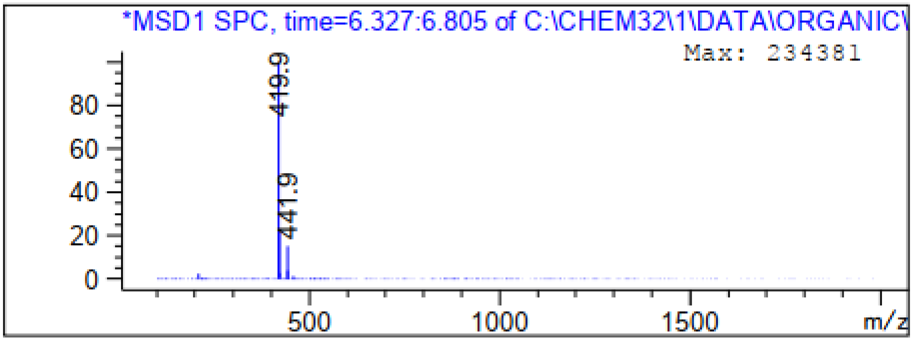

Figure S103. ESI-MS of A26

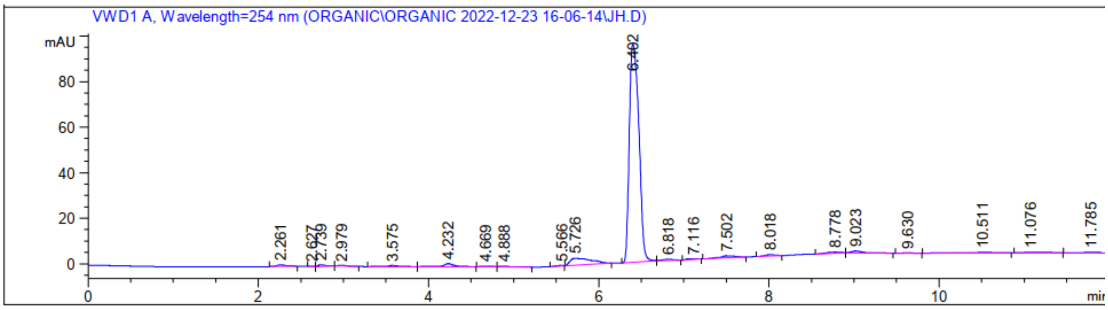

Figure S104. HPLC purity of A26

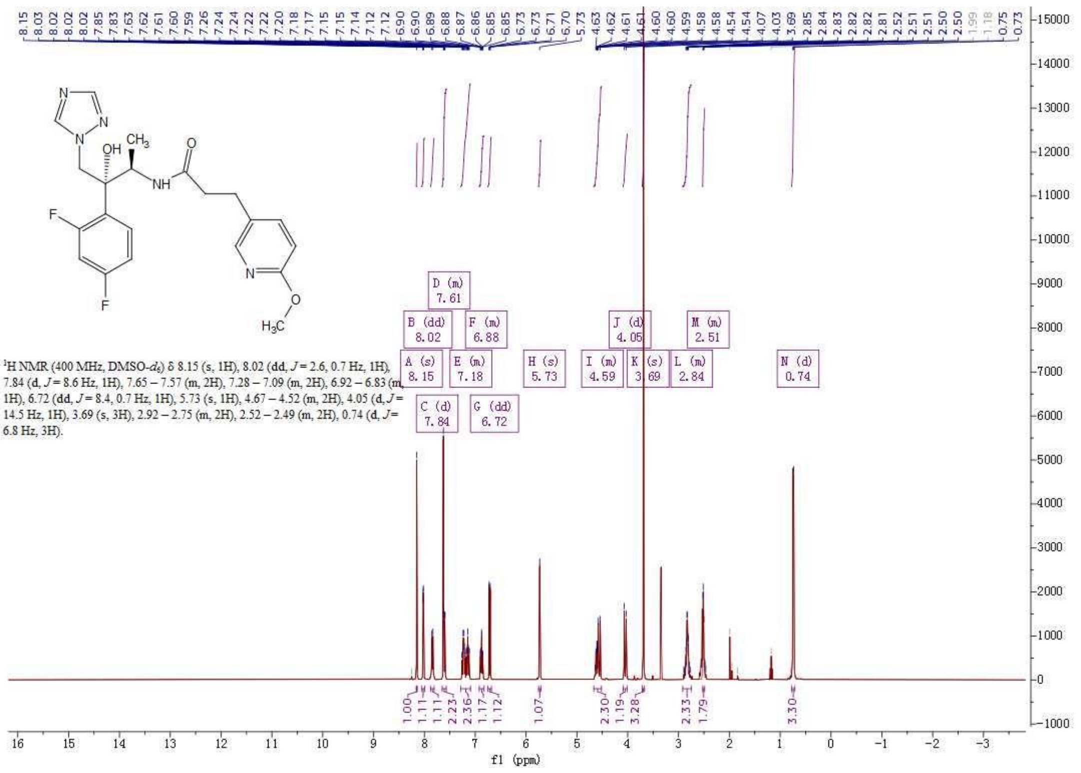

Figure S105. ^{1}H-NMR of A27

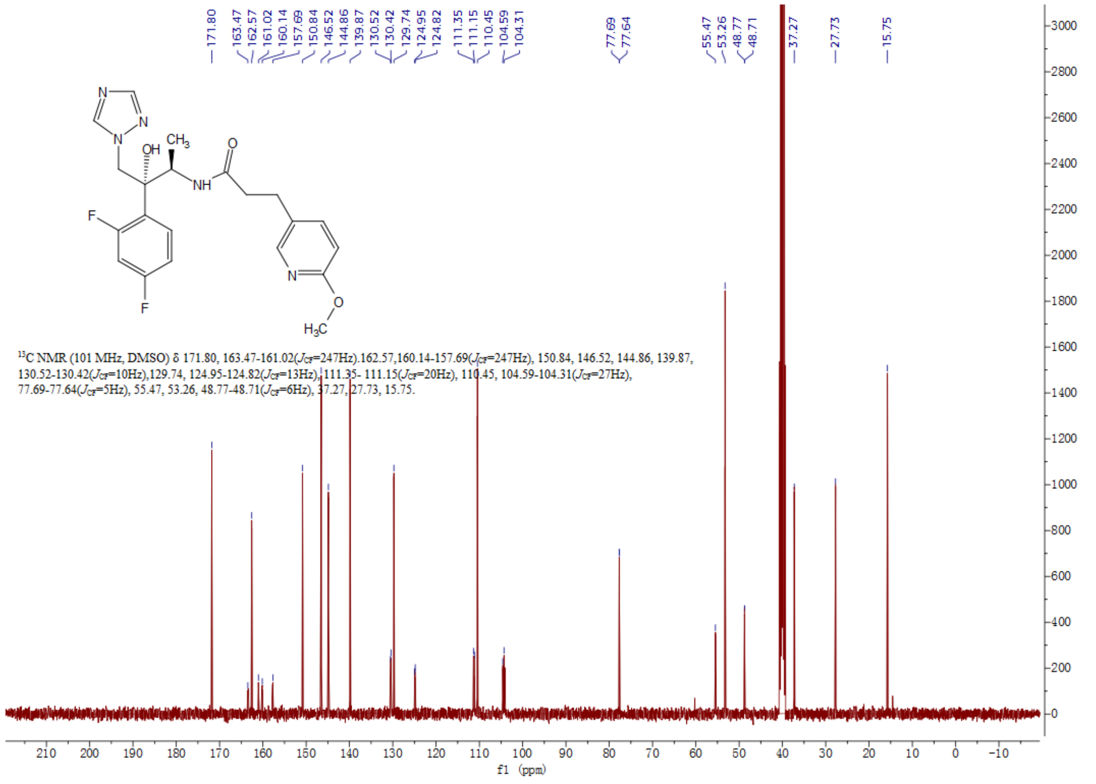

Figure S106. ^{13}C-NMR of A27

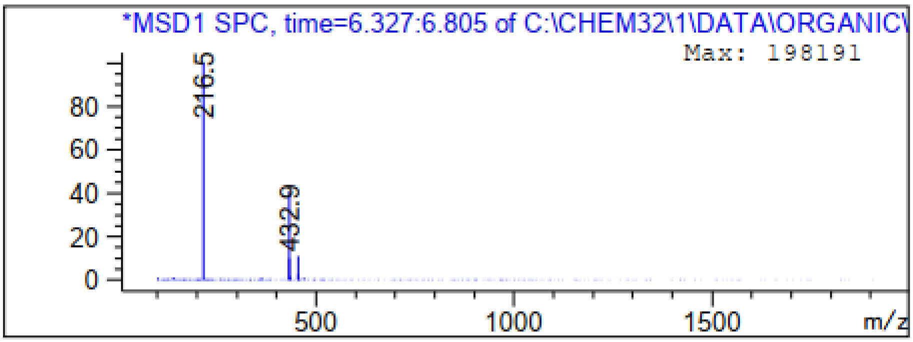

Figure S107. ESI-MS of A27

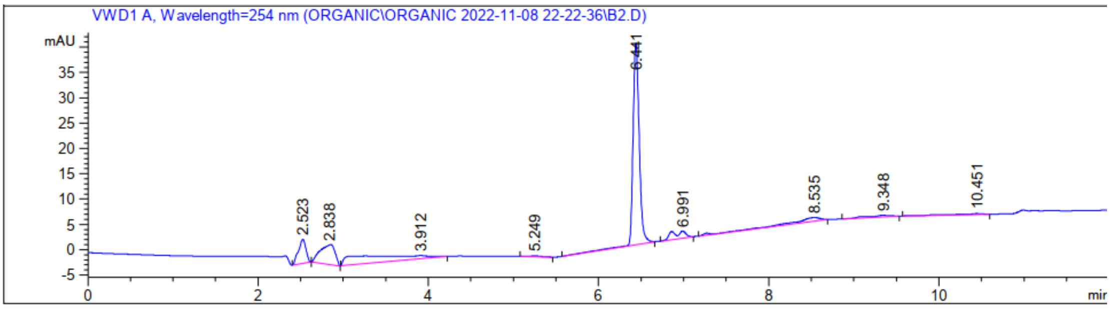

Figure S108. HPLC purity of A27
